# Visualization and quantitation of electronic communication pathways in a series of redox-active pillar[6]arene-based macrocycles

**DOI:** 10.1038/s42004-020-00363-4

**Published:** 2020-08-13

**Authors:** Mehdi Rashvand Avei, Sedigheh Etezadi, Burjor Captain, Angel E. Kaifer

**Affiliations:** Department of Chemistry, University of Miami, Coral Gables, FL 33124 USA

**Keywords:** Self-assembly, Synthetic chemistry methodology

## Abstract

While oxidized pillar[5]arenes with 1-5 benzoquinone units are known, very few examples of oxidized pillar[6]arenes have been reported. We describe here the synthesis, characterization and electrochemical behavior of a series of macrocyclic hosts prepared by the stepwise oxidation of 1,4-diethoxypillar[6]arene, resulting in high-yield and high-purity isolation of two constitutional isomers for each macrocycle, in which two, three or four 1,4-diethoxybenzene units are replaced by benzoquinone residues. A careful structural comparison with their counterparts in the pillar[5]arene framework indicates that the geometries of the macrocycles are better described as non-Euclidean hyperbolic hexagons and elliptic pentagons, respectively. A comprehensive computational study to determine anisotropic induced current density (ACID) allows us to visualize and quantify through-space and through-bond communication pathways along the macrocyclic belt. Experimental and simulated voltammetric data, as well as UV-vis spectra, of the new macrocycles afford insights into the various electronic communication pathways in these compounds.

## Abstract (web summary)

Partially oxidised pillar[n]arenes exhibit interesting redox behaviour, but the pillar[6]arenes are poorly characterised in this context. Here stepwise oxidation of 1,4-diethoxypillar[6]arene allows detailed study of the structural and electrochemical properties of molecules with 2, 3, and 4 oxidised units.

## Introduction

The pillar[n]arenes^1,2^ are a family of macrocycles, which have attracted considerable attention in the last 12 years. Research on pillar[n]arenes has reached a high level of maturity, with a good number of reviews covering the large body of research work on these macrocyclic hosts^3–11^. Perhaps one of the most important features of pillar[n]arenes is that they can be functionalized with ease in a variety of ways^12^. We are particularly interested in their oxidized forms, in which some of the 1,4-dialkoxybenzene (or hydroquinone) units in the macrocycle are replaced by benzoquinone residues. In 2015, we first reported the voltammetric behavior of pillar[5]quinone^13^, in which all five aromatic units are benzoquinone residues. This highly oxidized macrocycle exhibits an interesting cathodic voltammetric behavior, which was rationalized by the minimization of electrostatic repulsions between the anionic semiquinone residues resulting from electrochemical reduction. Later on, we investigated a series of pillar[5]arenes, in which variable levels of oxidation led to macrocycles containing anywhere from one to five benzoquinone residues^14^. The cathodic voltammetric behavior for all these compounds suggests the presence of through-space communication among the quinone units within the pentameric macrocyclic framework. This conclusion was confirmed by detailed computational studies^14^. Other groups have also reported electrochemical data on pillar[5]quinones^15,16^. However, expansion of the oxidized macrocycles to the pillar[6]arene framework has proven synthetically challenging, so there are only a few host-guest reports on a pillar[6]arene compound in which only one of the aromatic units is replaced by a quinone^17,18^. To the best of our knowledge, there are no reports on pillar[6]arenes containing two or more quinone residues.

Here, we report our recent synthetic and characterization work on different constitutional isomers of oxidized derivatives of pillar[6]arenes containing from 1 to 4-benzoquinone units, as well as the electrochemical properties of these compounds. Using anisotropic current-induced density theory (ACID), along with other density functional theory (DFT) computations, we identify distinct mechanisms for the electronic communication among quinone units within this family of compounds, taking place (through-bond and through-space) along the macrocyclic belt or through direct contact across the equatorial plane of the macrocycle.

## Results and discussion

### Synthesis

**Fig. 1:**
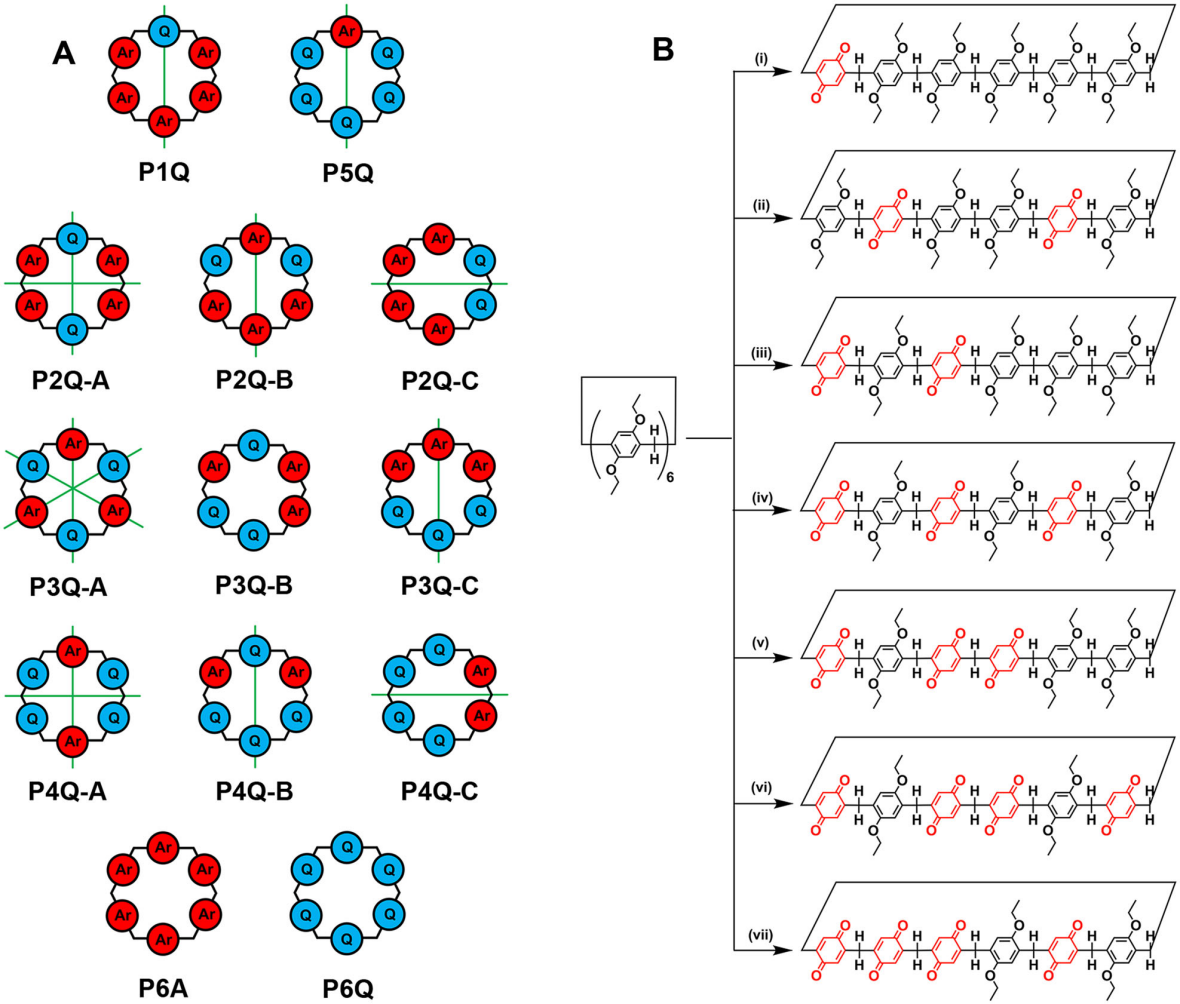
Structures of the partially oxidized pillar[6]quinones. **a** Schematic representation of oxidized derivatives of per-ethoxypillar[6]arene (P6A). The red circles labeled ‘Ar’ symbolize 1,4-diethoxybenzene units and the blue circles labeled ‘Q’ represent 1,4-benzoquinone units. The green lines represent C2 axes that are useful to determine the number of ^13^C or ^1^H NMR resonances for each macrocycle. **b** Structures and synthetic conditions used for the preparation of partially oxidized pillar[6]arenes. Reaction conditions: (i) 2.2, (ii) and (iii) 4.4, (iv), (v) 6.6, and (vi) and (vii) 11 equiv of (NH_4_)_2_[Ce(NO_3_)_6_] in 1:1 mixture of DCM:THF, 20 h, room temperature.

The oxidation of the known macrocycle 1,4-diethoxypillar[6]arene^19^ may lead to a number of compounds, which are represented symbolically in Fig. 1a. For simplicity, we have elected to designate these compounds as P*n*Q, in which *n* represents the number of aromatic units oxidized to benzoquinones. While P1Q, P5Q, and P6Q each exists as a single compound, P2Q, P3Q, and P4Q may each exist as three constitutional isomers, which we designate as A, B, and C (Fig. 1a). Under our experimental conditions we were able to prepare and isolate P1Q, P2Q-A, P2Q-B, P3Q-A, P3Q-B, P4Q-A, and P4Q-B. With the single exception of P1Q^17^, all of these compounds were previously unknown. In general terms, our results indicate that the accumulation of adjacent benzoquinone units in the macrocycle is not favored^20^. However, our yields for the isolated oxidized compounds (P1Q through P4Q) were satisfactory, ranging from a robust 73.2% for P1Q to just 18.5% for P4Q-B. While we could detect trace amounts of P5Q and P6Q in some of our reaction mixtures, we were unable to isolate these compounds in pure form. Not even a large excess of the oxidizing agent was enough to drive the preparation of P5Q or P6Q macrocycles in amounts large enough to make isolation possible. The specific structures of the compounds obtained and isolated in our experiments are given in Fig. 1b, as well as the experimental conditions used in their synthesis.

### ^1^H NMR analysis

**Fig. 2:**
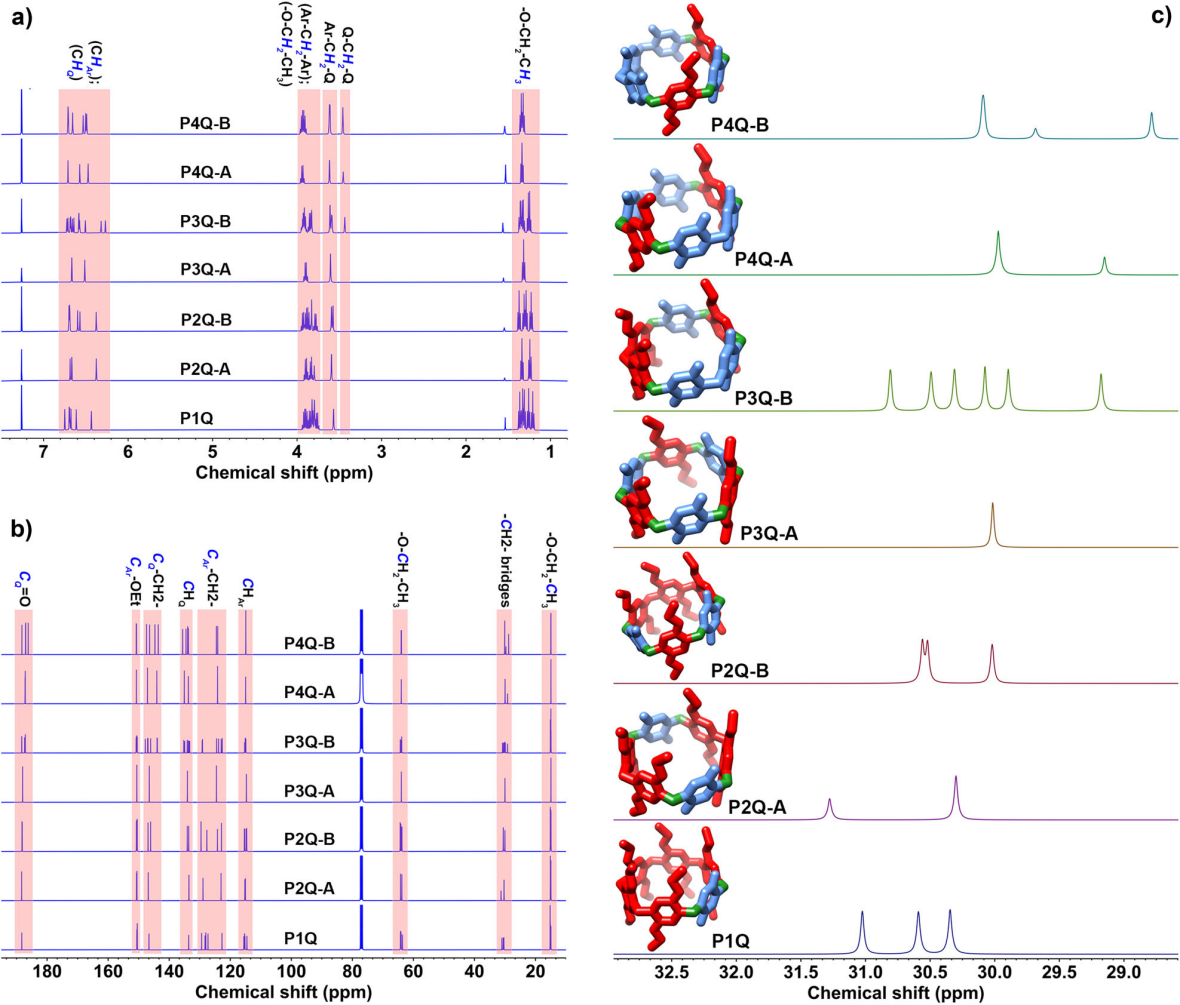
NMR spectra. **a** ^1^H NMR (500 MHz) and **b** ^13^C NMR (100 MHz) spectra regions of the partially oxidized pillar[6]arenes in CDCl_3_. **c** Partial ^13^C NMR spectra of the partially oxidized pillar[6]arenes showing the bridging methylene carbon peaks. Embedded presentation of each macrocycle shows the various methylene bridges in different colors as green = (Q–CH_2_–Ar), red = (Ar–CH_2_–Ar), and blue = (Q–CH_2_–Q).

Proton NMR spectra (Fig. 2a) of the macrocycles were analyzed to find the most revealing signals to ascertain the regio-selectivity of the macrocycles obtained from pillar[6]arene oxidation. In this regard, the different chemical environments possible for the methylene bridges, which can be located between two quinone units (Q–C**H**_**2**_–Q), between an aryl and a quinone unit (Ar–C**H**_**2**_–Q) or between two aryl units (Ar–C**H**_**2**_–Ar), should result in resonances in specific chemical shift regions. In spite of the overlap between the proton signals of Ar–C**H**_**2**_–Ar and those of the methylenes on the ethoxy chains attached to the aromatic units, the proton signals of the Ar–C**H**_**2**_–Q and Q–C**H**_**2**_–Q bridges afford a reliable criterium to distinguish among isomers. For instance, the ^1^H NMR spectrum of P1Q (Supplementary Fig. 2) shows a signal at 3.57 ppm (4H), which is attributed to the protons of two methylene bridges on either side of the existing quinone unit. Therefore, the presence of the proton signal for Ar–C**H**_**2**_–Q at a chemical shift of about 3.6 ppm is helpful to identify the various isomers. Additionally, the absence of the proton signal for Q–C**H**_**2**_–Q in the ^1^H NMR spectra of P2Q-A, P2Q-B, and P3Q-A (Supplementary Figs. 3–5) confirms that the quinone units are not adjacent in these macrocycles. On the contrary, the existence of the proton signal of Q–C**H**_**2**_–Q in the ^1^H NMR spectra of P3Q-B, P4Q-A, and P4Q-B (Supplementary Figs. 6–8) provides strong support to distinguish them from their corresponding isomers. The exact assignment of proton signals in the ^1^H NMR spectra of these macrocycles is rather complex for compounds with low symmetry and quite simple for the more symmetric compounds, such as P2Q-A, P3Q-A, and P4Q-A. For instance, the ^1^H NMR spectrum of P3Q-A (Supplementary Fig. 5) consists of two singlets above 6 ppm, corresponding to the aromatic protons on the benzoquinone (BQ) and 1,4-diethoxybenzene (1,4-DEB) residues, a quartet for the methylene protons on the ethoxy groups, a singlet for the methylenes connecting the aromatic units, and a triplet for the methyl protons. The spectrum of P2Q-A shows two signals of equal intensity for the methyl protons, because of the lack of C2 axes crossing over opposing aryl units. Similarly, there are two signals for the methylenes on the ethoxy groups and for the aromatic protons. On the other hand, the ^1^H NMR spectra of P3Q-A and P4Q-A, shows only one triplet for the methyl protons, in accordance with symmetry predictions, as shown in Fig. 1.

### ^13^C{^1^H} NMR analysis

**Fig. 3:**
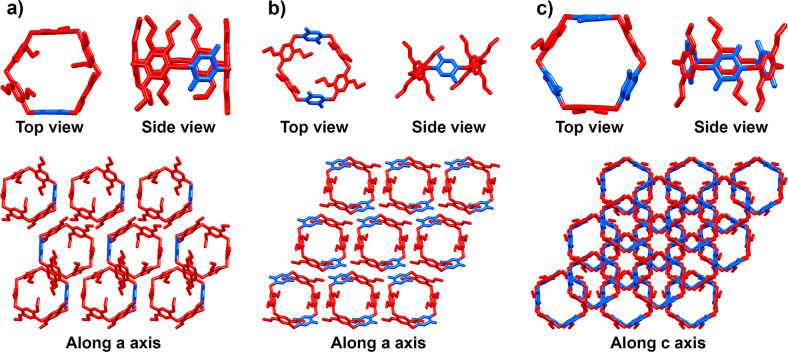
X-Ray crystal structures. Single crystal X-ray structure of **a** P1Q, **b** P2Q-A, and **c** P3Q-A. Hydrogen atoms and solvent molecules were removed for clarity. Blue and red units refer to electron-poor benzoquinone and electron-rich 1,4-diethoxybenzene species, respectively.

After recording the ^13^C NMR and DEPT spectra of the new macrocycles (Supplementary Figs. 9–15), we can define nine spectral regions corresponding to (i) methyl carbon atoms on the ethoxy chains (14–16 ppm), (ii) bridging methylene carbon atoms (28–32 ppm), (iii) methylene carbon atoms on the ethoxy chains (62–65 ppm), (iv) aryl carbon atoms having one hydrogen attached (**C**_Ar_H; 114–116 ppm), (v) aryl carbon atoms attached to bridging methylenes (**C**_Ar_–CH_2_; 122–130 ppm), (vi) quinone carbon atoms having one hydrogen attached (**C**_Q_H; 133–136 ppm), (vii) quinone carbon atoms attached to bridging methylenes (**C**_Q_–CH_2_; 143–149 ppm), (viii) aryl carbon atoms attached to ethoxy chains (**C**_Ar_–O-Et; 150–151 ppm), and (ix) carbonyl carbon atoms on quinone units (**C**_Q_ = O; 186–189 ppm) as shown in Fig. 2b. Among all these ^13^C NMR signals, the chemical shifts for the bridging methylene carbon atoms are very significant and allow us to determine the structure of any pillar[n]arene oxidation product. The ^13^C NMR spectra of P3Q-A shows a single peak for the bridging methylene carbons, while compounds P2Q-A and P4Q-A exhibit two peaks in the same spectral region. In fact, this region of the ^13^C NMR spectra clearly illustrates the symmetry or lack thereof in each of the macrocycles (see Fig. 3c). Notably, P3Q-B gives rise to six different signals for the connecting methylene carbons due to the complete absence of suitable symmetry elements.

Furthermore, signals with a chemical shift under 29.5 ppm are only observed for three of the compounds, P3Q-B, P4Q-A, and P4Q-B, which is consistent with the fact that these are the only macrocycles with methylenes connecting two quinone units (Q–CH_2_–Q). The single peak observed for P3Q-A provides the typical chemical shift for methylenes connecting a quinone and an aromatic unit (Q–CH_2_–Ar). Therefore, the signals corresponding to methylenes bridging two aromatic units (Ar–CH_2_–Ar) appear at higher chemical shifts in this region. These arguments can be equally applied to other regions of the ^13^C NMR spectra of these compounds (Supplementary Fig. 16). As indicated before, a similar analysis of the bridging –CH_2_– peaks in the ^1^H NMR spectra is hampered by the fact that these signals partially overlap with those of the –OCH_2_– methylenes on the ethoxy chains attached to the aromatic units. In spite of these complications, similar to those previously observed with related quinone-containing macrocycles based on the pillar[5]arene framework, we confirmed the structure of the newly prepared macrocycles based on the ^13^C NMR spectra and the integrations of the major peak groups on the ^1^H NMR spectra.

### X-ray crystal structures

We obtained single crystals of P1Q, P2Q-A, and P3Q-A suitable for single-crystal X-ray diffraction analysis (Supplementary Figs. 23–25). Crystals of P1Q and P3Q-A were grown by slow evaporation of a chloroform/dioxane solution at room temperature, while those of P2Q-A were obtained by slow evaporation of a dichloromethane/benzene solution at 7 °C. Figure 3 shows the individual structures of these molecules in the solid state and their packing arrangements to form layers. All three crystal structures included co-crystallization of solvent molecules (Supplementary Figs. 17–19), which were omitted, as well as the hydrogen atoms, from Fig. 3 and Supplementary Fig. 20. It is important to note that all the aromatic and quinone units in P1Q and P3Q-A are approximately oriented in directions parallel to the main molecular axis. As these molecules pack in a two-dimensional layer, there are numerous intermolecular quinone-aromatic contacts, which should contribute to stabilizing the assembly (Supplementary Figs. 21–22). In fact, both of these crystals show a strong red-orange color, which is consistent with the presence of charge transfer complexes in their lattices, since 1,4-diethoxypillar[6]arene is colorless. In contrast to this, P2Q-A shows a different arrangement, with two of the aromatic units and the two quinone residues aligned at around 45 degrees from the main molecular axis and the remaining two aromatic units oriented almost perpendicular to the rest of the units. This molecular arrangement affects the two-dimensional layer packing. In fact, the different intra- and intermolecular structure of P2Q-A is indeed related to the presence of numerous benzene molecules in the crystal lattice, resulting from the crystallization conditions, and leading to π-π stacking contacts with both the quinone and the 1,4-diethoxybenzene units of the macrocycle, which explain the orange-red color of this crystal. In contrast, both P1Q and P3Q-A crystallize with chloroform molecules, which tend to occupy the macrocyclic cavities instead, fostering the roughly parallel alignment of all the aromatic and benzoquinone units. The resulting structural differences are apparent in Supplementary Figs. 17–19.

**Fig. 4:**
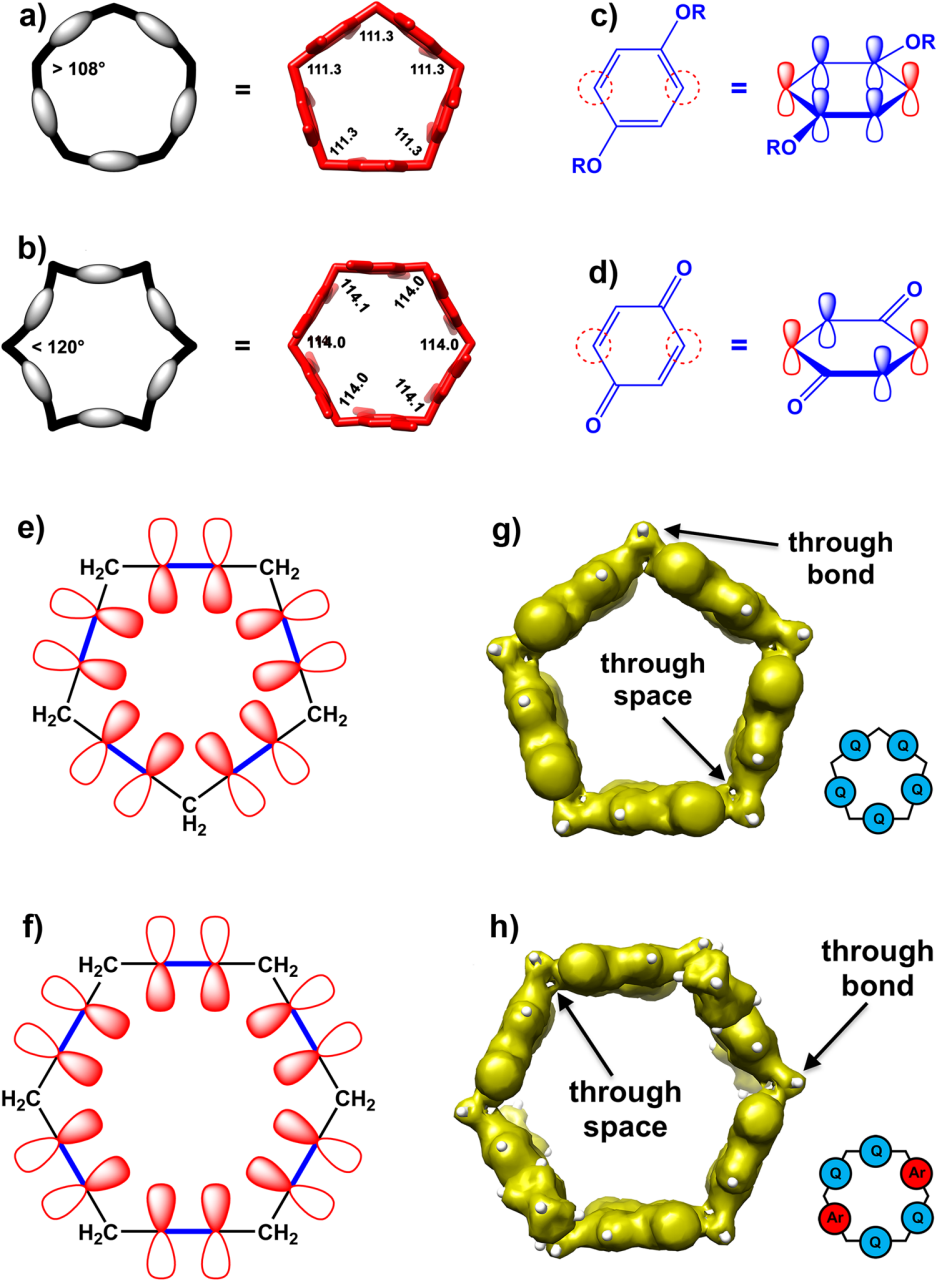
Non-Euclidean pillar[n]arene shapes and p orbital mixing leading to ACID plots. Schematic representation of **a** a non-Euclidean elliptic P’5 A and **b** a non-Euclidean hyperbolic P6A depicting the interior angles of their corresponding optimized structures on the right panel. (Compounds optimized at the M062X/6–31 G + (d,P) level in the presence of solvent (CH_2_Cl_2_). The bright ovals on their sides are representative of bent benzene rings). **c** 1,4-dialkoxybenzene in which R represents a carbon chain and **d** benzoquinone monomers and their corresponding 2p atomic orbitals corresponding to endocyclic π bonds; the ones in red show their 2p orbitals located at the 2 and 5 positions. **e** and **f** depict the orientation of the p orbitals of either the quinone or the aromatic unit’s carbon atoms on the equatorial belt in pillar[5]arene and Pillar[6]arene compounds, respectively, and also their oxidized derivatives. ACID plots of **g** P’5Q and **h** P4Q-A compounds at an iso-surface value of 0.025. The ACID boundary surface indicates strong through-bond interactions and weaker through-space interactions.

### Structural comparison of the pillar[5]arene and the pillar[6]arene frameworks

The results presented in this work and our previous work on oxidized pillar[5]arenes open significant questions on the structural and electronic differences between these two related, but geometrically different macrocyclic frameworks. Indeed, pentagonal and hexagonal forms differ in the value of their interior angles, 108° and 120° respectively. On the other hand, the sp^3^ hybridization of the bridging methylenes in pillar[n]arenes would favor a bonding angle of 109.28°, which may result in variable degrees of angle strain in pillar[5]arenes and pillar[6]arenes. In order to quantitate the angle strain we analyzed the structures of a number of these compounds using DFT calculations [at the M062X/6–31 G + (d,p) level of theory]. We compared our energy minimized DFT structures to X-ray structures, where available, and found that both structures were essentially identical, excluding some deviations stemming from the freely rotating methoxy or ethoxy groups (Supplementary Figs. 26–27 and Supplementary Table 1). These structural similarities afford a good level of confidence in our computational results. The interior angles measured in our DFT computed structures are shown in Supplementary Figs. 28 and 29. Notice that we use the designation P’*n*Q for the pillar[5]arene macrocycles to differentiate them from P*n*Q, i.e., the pillar[6]arene macrocycles. In both cases, *n* refers to the number of quinone units. For regular Euclidean pentagons and hexagons, the sum of the interior angles are 540° and 720°, respectively, whereas these values are 556.5° and 684.1°, for P’5 A and P6A, respectively, according to our computational results. The computed angular sum values are 16.5° higher and 35.9° lower for P’5 A and P6A, respectively. As a result of this structural stress, P’5 A and P6A attempt to mitigate the angle strain by distributing it over their sides and vertices. While close to the angle of a regular pentagon (108°), the bond angle of the sp^3^ hybridized bridging methylenes (109.28°) is further away from that of a regular hexagon (120°). These macrocyclic structures need to distribute some of the tension over their sides, composed of either quinone or aromatic units, resulting in non-Euclidean geometries and subsequent bending of the quinone or aromatic units, as shown in Fig. 4a, b (see Supplementary Figs. 28–29 regarding interior angles of other constitutional isomers).

Additionally, the total angular strains can be measured as the difference between the total angular sums in the computed structures and the corresponding values expected if the bridging methylenes were under no strain at all (5 × 109.28° in P’5 A and 6 × 109.28° in P6A). Using this definition, we obtained values of +10.1° and +28.4° for P’5 A and P6A, respectively, or +2.0° and +4.7° per bridging methylene. Notice that as quinone units replace aromatic groups in P’5 A, the overall angular strain decreases, stemming from the lower rigidity of the non-aromatic-quinone units, which can adjust better to fit the pentagonal assembly. The same trend is observed with the hexagonal macrocycles, although P6Q, with and interior angle of 112.4°, retains a larger overall angular strain than P’5Q, with an interior angle of 109.8°, which is quite close to the theoretical sp^3^ angle value. In general, the lower angular strain observed in the oxidized macrocycles results from the higher rigidity of the aromatic units compared to the quinone residues. The larger angular strain present in the pillar[6]arene macrocycles has been proposed as an important reason for their less efficient synthesis, and may also be responsible for the poor stability of the fully oxidized P6Q macrocycle, which has not been isolated yet, while the low angular strain prevalent in P’5Q facilitates its preparation and isolation^19,21^.

### Anisotropic current-induced density study

Pillar[5]arene and pillar[6]arene compounds and their oxidized derivatives are members of the paracyclophane family because of the linkage of the aromatic and benzoquinone rings through their 2 and 5 positions, as shown in Fig. 4c and d. Moreover, the p orbitals on the carbon atoms of the aromatic and benzoquinone units are also depicted in the same Figures. The p orbitals of the carbon atoms located at the 2 and 5 positions are intentionally shown in red (Fig. 4c, d). This plain representation of the p orbitals is helpful to better understand the through-space electronic communication among the constituent units of these macrocycles. The wider interior angle of the pillar[6]arene framework as compared to that of the pillar[5]arene framework lowers the extent of overlap between the neighboring p orbitals (Fig. 4e and f). Anisotropic Current-Induced Density (ACID) theory is an exhaustive interpretation of the same concept, providing an accurate quantification and visualization of electronic communication pathways.

To investigate the electronic delocalization in both pillar[5]arene and pillar[6]arene macrocyclic frameworks, we used ACID as a general method^22^. The ACID can be interpreted as a representation of delocalized electrons and can be used to assess any type of conjugation in any kind of state (ground state, excited state, transition state, etc.) of a given molecular system. By computing and displaying ACID iso-surfaces, we can control the sensitivity of the method and also quantify conjugation effects, as we visualize weak conjugation by using small iso-surface values. To provide a measurement of the extent of conjugation between different molecular components, we need to find the critical iso-surface value (CIV) at which the topology of the ACID boundary surface breaks into independent enveloping surfaces. As the CIV between two atoms or groups decreases, their conjugation becomes weaker (Supplementary Table 2). The ACID plots of P4Q-A and P’5Q compounds are shown in Fig. 4g, h. They show the presence of strong through-bond conjugation (ACID iso-surface includes the bridging methylenes) and weaker through-space interactions (ACID iso-surface connects adjacent benzene or quinone rings). Since these two ways of electronic communication (through-bond and through-space) between the constituent units of the macrocycle take place around the periphery or macrocyclic belt of the compound, we refer to it as “peripheral communication”.

All surveyed compounds fall in either pentagonal or hexagonal geometries and no significant structural changes can be expected by just replacing the aromatic units by non-aromatic ones in these frameworks. However, the changes in angle strain of the corresponding compounds, essentially originating from the presence of aromatic units, and also the impact of the angle strain, though subtle, on the degree of electronic communication are meticulously determined by the quantifying parameter, CIV, as defined in ACID theory. From the schematic representation of equatorial p orbitals (Fig. 4e and f), it is evident that the non-Euclidean geometries of pillar[5]arene and pillar[6]arene compounds also affect the extent of overlap of these p orbitals.

### Electronic absorption spectra

**Fig. 5:**
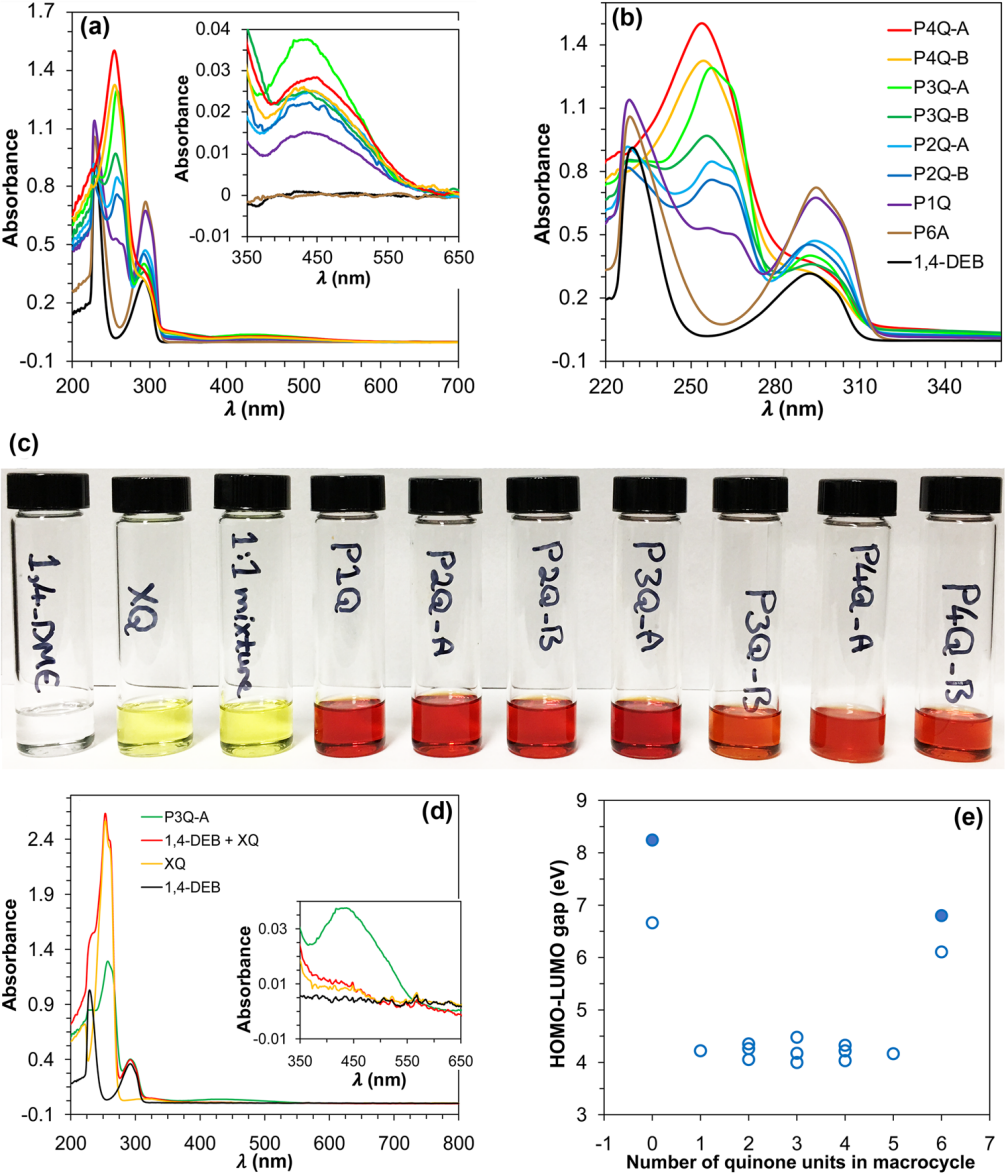
Electronic absorption spectra. **a**, **b**, **d** UV-vis spectra of the oxidized derivatives of P6A in CH_2_Cl_2_ solution. The concentration of macrocycle solutions is about 25 μM and the concentration of 1,4-DEB and *p*-xyloquinone (XQ) as well as a mixture of 1,4-DEB and XQ solutions is about 0.1 mM. **c** From left to right, 0.1 mM CH_2_Cl_2_ solutions of 1,4-DEB, XQ, a 1:1 mixture of 1,4-DEB and XQ, P1Q, P2Q-A, P2Q-B, P3Q-A, P3Q-B, P4Q-A, and P4Q-B, respectively. **e** Plot of the value of HOMO-LUMO gap of the corresponding compounds versus the number of quinone units present in the compounds (Calculated at M062X/6–31 G + (d,p) level including CH_2_Cl_2_ as an implicit solvent. The open circles are related to the macrocycles containing different number of quinone units. The filled circles correspond to 1,4-DEB and BQ representing fully reduced and fully oxidized monomers, respectively).

The UV-Vis spectra of the oxidized macrocycles in dichloromethane solution (Fig. 5a, b) show that as the number of quinone units increases, the intensity of the absorption in the 240-260 nm region rises, whereas the absorption at 290–300 nm decreases, which clearly establishes that the former absorption corresponds to the quinone units, while the latter is associated to the aromatic groups. Low intensity charge transfer absorptions were observed in the 350–650 nm region for partially oxidized macrocycles. P3Q-A, with alternate quinone and aromatic units, shows the most intense charge transfer absorption. From a statistical standpoint, P3Q-A possesses an equal number of aromatic and quinone units, providing the maximum level of charge transfer interactions either inter- or intramolecularly. We carried out control experiments to determine the nature of the charge transfer interaction.

In strong contrast with the colors of CH_2_Cl_2_ solutions of the surveyed macrocycles, CH_2_Cl_2_ solutions of pure *p*-xyloquinone (XQ) and an equimolar mixture of 1,4-DEB + XQ are the same (yellow), qualitatively confirming the lack of intermolecular charge transfer interactions (Fig. 5c). Additionally, the UV-vis spectrum of the equimolar mixture of 1,4-DEB + XQ does not show any charge transfer absorption band, again indicating that the charge transfer absorption of the compounds under study results from purely intramolecular interactions (Fig. 5d). As mentioned above, macrocycles containing various combinations of aromatic and quinone units are capable of intramolecular charge transfer interactions, stemming from the complementary interactions between the HOMO of 1,4-diethoxybenzene and the LUMO of benzoquinone. Figure 4e and f provides a clear visualization of such interactions between quinone and aromatic units, as electron-poor and electron-rich components, respectively. Since the source of charge transfer in these compounds is purely intramolecular, we define “the number of aromatic-quinone proximities” as a parameter that shows the number of methylene linkers connecting an aromatic and a quinone unit. P3Q-A and P1Q-A have six and two aromatic-quinone proximities, respectively, which account for their maximum and minimum charge transfer absorptions (Supplementary Table 4). As for other compounds having the same numbers of aromatic-quinone proximities, the greater the angle strain, the further their corresponding structures shrink inwardly, providing greater HOMO-LUMO overlaps. This interpretation accounts for the relative charge transfer intensities in P4Q-A, P4Q-B, P3Q-B, P2Q-A, and P2Q-B (inset of Fig. 5a and Supplementary Table 4). The low intensity is fully expected, given the low macrocycle concentration (25 μM) in these experiments and the essentially intramolecular nature of these charge transfer interactions.

Comparing the changes in energy and geometry with respect to a reference system without conjugation is the simplest method to estimate the extent of conjugation in a system^23^. As shown in Fig. 5e and Supplementary Table 5, we calculated the value of HOMO-LUMO gap of the macrocycles using DFT methods. The calculations support the presence of a strong conjugation throughout the macrocycles, stressing the existence of the peripheral pathway—as shown by the ACID calculations—between the π bonds of the constituent units, which result in a diminished HOMO-LUMO gap compared with those of monomeric 1,4-diethylbenzene and benzoquinone. In complementary compound pairs (P’5 A and P’5Q, P’1Q and P’4Q, P’2Q-A and P’3Q-A, P’2Q-B and P’3Q-B, P6A and P6Q, P1Q and P5Q, P2Q-A and P4Q-A, P2Q-B and P4Q-B, and P2Q-C and P4Q-C), as shown in Fig. 1 and Supplementary Fig. 30, the HOMO-LUMO gap values hover around the same value, resulting from the utilization of canonical molecular orbital method, which is tightly adapted to the symmetry of starting materials. The conjugation in the macrocycles supports the presence of intramolecular charge transfer as well. Performing the same calculations on partially oxidized derivatives of pillar[5]arene compounds (shown in Supplementary Fig. 30) confirms the same trend in the obtained results as shown in Supplementary Fig. 31 and also Supplementary Table 5.

Clearly, the replacement of aromatic units by benzoquinone groups shifts the corresponding absorption maxima to shorter wavelengths, identical to those of the isolated aromatic compounds (Supplementary Table 3). Although the measured wavelength shifts are small, they are fully consistent with reported observations on the spectroscopic properties of cyclophanes^24^, where bending forces on the aromatic units are known to shift the absorption maxima to longer wavelengths. In our case, these data support the reduction of angular strain in pillar[n]arenes as the quinone units gradually replace the aromatic groups.

### Electrochemistry

In general terms, the electrochemical behavior of quinones is well understood^25^. In non-protic solvents, these compounds undergo two consecutive one-electron reductions. The first reduction process leads to an anion radical (Eq. 1) and the second reduction yields a dianion (Eq. 2). The first electron transfer process is typically reversible, while the second reduction is quasi-reversible and much more sensitive to the presence of protic impurities (water, alcohols, etc.) because quinone dianions are efficient hydrogen bonding acceptors^26^.

1 $${\mathrm{Q}} + e^ - \rightleftharpoons {\mathrm{Q}}^{ \bullet - }$$

2 $${\mathrm{Q}}^{ \bullet - } + e^ - \rightleftharpoons {\mathrm{Q}}^{2 - }$$

**Fig. 6:**
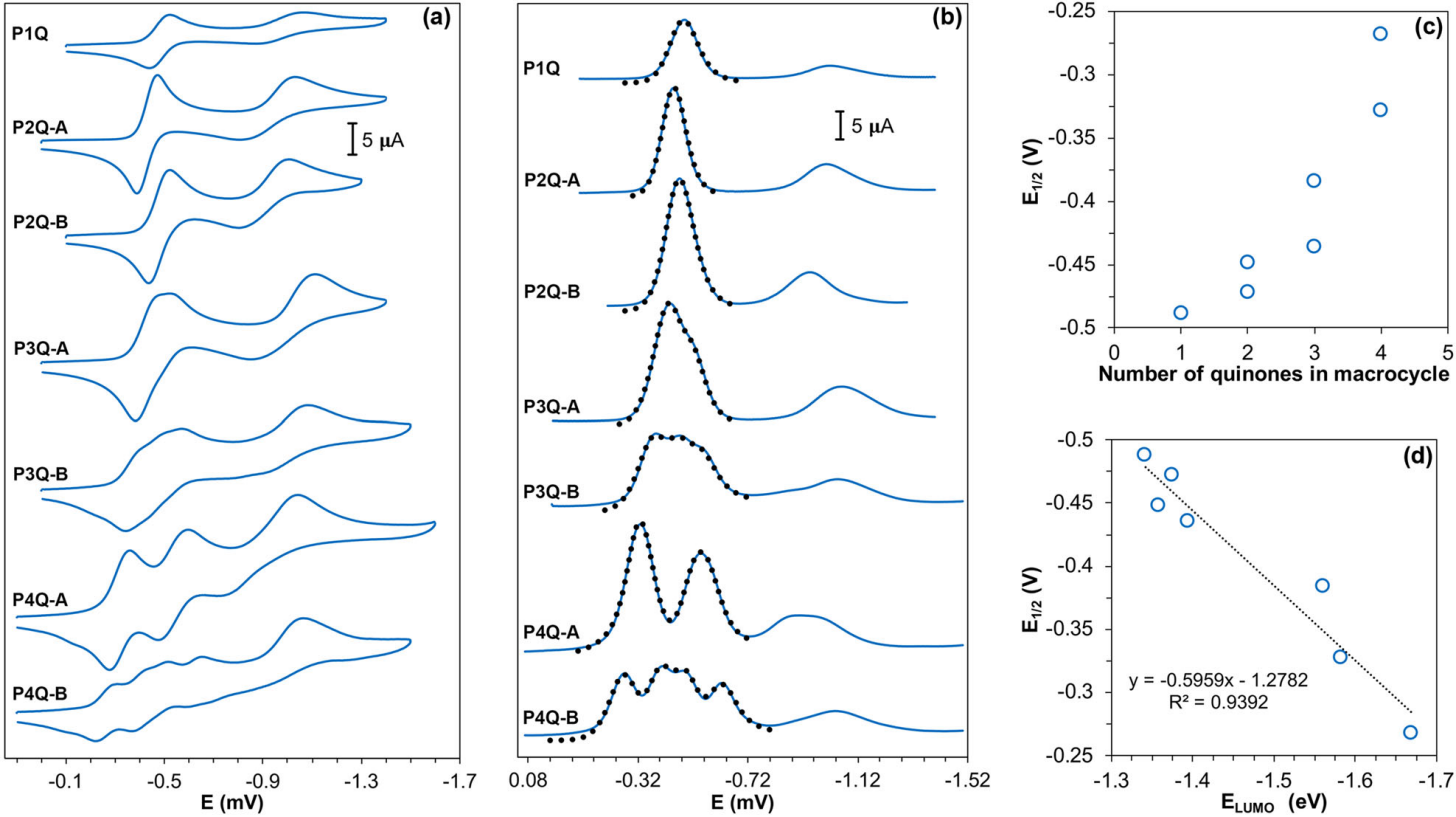
Voltammetric results. **a** Cyclic voltammograms (CV) and **b** square wave voltammograms (SWV) of 0.25 mM oxidized derivatives of perethylated-pillar[6]arene using a glassy carbon working electrode (0.07 cm^2^) in 0.1 M CH_2_Cl_2_ solution of TBAP at 100 and 60 mV/s, respectively. **c** First reduction potential (V vs Ag/AgCl) in dichloromethane solution for the quinone-containing pillar[6]arenes as a function of the number of quinone residues in the macrocycle and **d** the corresponding LUMO energies obtained at ωB97X level.

A preliminary assessment shows that the electrochemical behavior of the single quinone macrocycle P1Q follows the same reduction pattern in dichloromethane solution (see Fig. 6a), showing half-wave potentials of −0.47 and −1.02 V vs Ag/AgCl, for each of the one-electron reduction processes. As is commonly observed with many quinones in cyclic voltammetric (CV) experiments, the set of waves corresponding to the second reduction is relatively broader and the peak-to-peak potential splitting $( {{\Delta}E_p})$ is larger than that observed for the first reduction process. This is partially explained by the slower rate of heterogeneous electron transfer for the Q^−^/Q^2−^ redox couple compared to the Q/Q^−^ redox couple. Here, we will focus our attention on the first quinone reduction processes, since the second reduction processes are more affected by a number of factors that typically make these waves broader and less intense than those corresponding to the first reduction, and, thus, less amenable to quantitative study.

The simple electrochemical behavior of P1Q may become more complicated in the macrocycles containing two or more quinone units because of the possibility of electronic communication between them. However, the cathodic voltammetric behavior of P2Q-A shows (Fig. 6a) a CV pattern similar to that of P1Q, indicating that the level of communication between the two quinone units is not significant. The voltammogram shows a single set of waves in the potential region corresponding to the Q/Q^−^ reduction, as would be expected for two independent, but identical quinone units. Therefore, the first set of waves corresponds to the uptake of two electrons by P2Q-A to form a dianion. The CV behavior for P2Q-B is almost identical to that of P2Q-A, suggesting that as long as the two quinone units are separated by at least one aromatic, 1,4-diethoxybenzene residue no significant communication between the quinones takes place. In contrast to this, the CV of P3Q-A shows some splitting in the set of waves corresponding to the first reduction. Clearly, uptake of the first two electrons takes place at similar potentials, but the uptake of the third electron is affected by the charges created by the uptake of the first two electrons, leading to a shoulder in the reduction wave. Similarly, the CV behavior of P3Q-B, P4Q-A, and P4Q-B also shows wave splitting in the potential region corresponding to the first reduction wave.

Both P2Q-B and P3Q-A have quinone units separated by one aryl unit, bringing into question the level of communication between them. In order to conclusively define the reversibility of the electrochemical processes, we convoluted the CVs of the macrocycles using semi-integral techniques. The results (Supplementary Figs. 32C–38C) indicate that the corresponding electron transfer reactions are best described as quasi-reversible. Additionally, we also applied the same method to CVs previously obtained with the oxidized derivatives of pillar[5]arene, and we reached the same conclusion, that is, their electrochemical reduction processes are also in the quasi-reversible regime. It is also noteworthy that the study of the electrochemical behavior of oxidized pillar[5]arene derivatives in both dichloromethane and acetonitrile solutions confirmed that solvent effects are negligible. In the case of pillar[6]arene derivatives, their low solubility in acetonitrile limited our work to dichloromethane solutions.

To better evaluate the electrochemical behavior of the compounds, however, we employed square wave voltammetry (SWV), a technique which has higher sensitivity than cyclic voltammetry (CV) and also generates a well-defined faradaic response. In order to determine the half-wave potentials associated with the first reduction wave in each of the quinone-containing macrocycles, we resorted to the digital simulation of square wave voltammetric data. By optimizing the fitting of the simulated voltammograms to the experimental data we can obtain the half-wave potentials and heterogeneous electron transfer rate constants for each of the individual one-electron reduction processes detected in the Q/Q^-^ potential region. The experimental and optimized simulated results are shown in Fig. 6b and the corresponding reduction potentials are given in Table 1. Similar half-wave potentials were obtained by taking the first derivative of the SWV plots (see Supplementary Figs. 32B–38B for illustrative examples). Additional parameters obtained from the fitting of the digital simulations to the experimental SWV data are given in Table 1.

**Table 1:** Electrochemical data.

Compound	D	E_1_^0^	E_2_^0^	E_3_^0^	E_4_^0^	k_1_	k_2_	k_3_	k_4_	α_1_	α_2_	α_3_	α_4_
P1Q	4.02	−0.464	–	–	–	0.90	–	–	–	0.345	–	–	–
P2Q-A	2.26	−0.418	−0.480	–	–	1.05	0.82	–	–	0.708	0.172	–	–
P2Q-B	5.50	−0.425	−0.504	–	–	0.98	0.66	–	–	0.692	0.205	–	–
P3Q-A	2.91	−0.364	−0.442	−0.514	–	0.54	1.93	1.09	–	0.681	0.157	0.193	–
P3Q-B	8.35	−0.322	−0.486	−0.568	–	0.40	0.50	0.44	–	0.639	0.133	0.153	–
P4Q-A	7.50	−0.261	−0.320	−0.526	−0.591	0.11	1.38	0.76	0.67	0.497	0.484	0.136	0.159
P4Q-B	4.70	−0.228	−0.412	−0.501	−0.638	0.50	0.80	0.78	0.54	0.644	0.100	0.110	0.175

Diffusion coefficients (×10^−5^ cm^2^/s), half-wave potentials (V vs Ag/AgCl), and heterogenous rate constants (×10^−2^ cm/s) for the one-electron reductions steps of the oxidized derivatives of per-ethoxypillar[6]arene obtained from digital simulations of the SWV data in Fig. 6b.

The two Q/Q^−^ half-wave potentials for P2Q-A are separated by 62 mV, while the difference increases slightly to 79 mV in P2Q-B, probably reflecting the closer proximity of the two quinone units in the latter macrocycle. In P3Q-A, the first two reductions are 78 mV apart, while the separation between the third and second reduction processes is 72 mV. Clearly, these values reveal a small degree of electronic communication between these quinone residues in all cases. In contrast, the two first reduction processes in P3Q-B are separated by 164 mV, clearly showing a larger extent of communication between the corresponding quinone units. P4Q-A constitutes an interesting case, as the first two half-wave potentials are relatively close (59 mV of separation) and the same is true for the last two half-wave potentials (65 mV apart). In other words, the four reduction steps are grouped in two main waves. The first two-electron wave corresponds to the first electron uptake by each of the two quinone pairs and the second wave corresponds to the second electron uptake by each quinone pair, leading to the formation of a tetraanionic macrocycle, [P4Q-A]^4−^. In P4Q-B the grouping of three adjacent quinones leads to the observation of four partially resolved waves, each corresponding to the uptake of one electron by a quinone unit.

As discussed earlier, ACID calculations illustrate various types of peripheral electronic communication, which may take place via through-bond and through-space mechanisms. As the number of quinone units in P6A derivatives increases, the interior angle becomes smaller and the minimum CIV value corresponding to through-space communication becomes greater, pointing to stronger communication and subsequently a more resolved CV. Regarding P3Q-A and P2Q-B, both possess the same size linker, one aryl unit, connecting the nonadjacent quinones to each other, but the bond angles in P2Q-B (shown in Supplementary Fig. 29 and Supplementary Table 4) are greater than those in P3Q-A, weakening the through-space communication and consequently yielding a more poorly resolved CV. Additionally, the observed half-wave potentials for the P*n*Q macrocycles are more negative than those measured with unsubstituted benzoquinone under the same conditions. This difference is attributed to the electron donating effect, occurring through peripheral pathways in the macrocycle, from the aromatic units connected via methylenes to the benzoquinone units.

In addition to peripheral (along the macrocyclic belt) mechanisms, we can visualize another potential communication mechanism resulting from the rotation of the aromatic or quinone units around the macrocyclic belt. In the solution phase, these macrocycles exhibit conformational flexibility, as the aromatic and quinone units can rotate around the methylene-methylene axis connecting them to the macrocycle. Indeed, the rotational freedom may be at least partially hampered by unfavorable steric interactions between the methoxy or ethoxy groups attached to neighboring 1,4-dimethoxybenzene (1,4-DMB) or 1,4-diethoxybenzene (1,4-DEB) units, respectively, but the rotation of the quinone units should be unhindered. We refer to this communication method as “equatorial communication,” since the neighboring units should meet each other through their carbonyl oxygen heads, bearing variable charge densities, depending on their states of reduction, and able to interact and even exchange electrons.

**Fig. 7:**
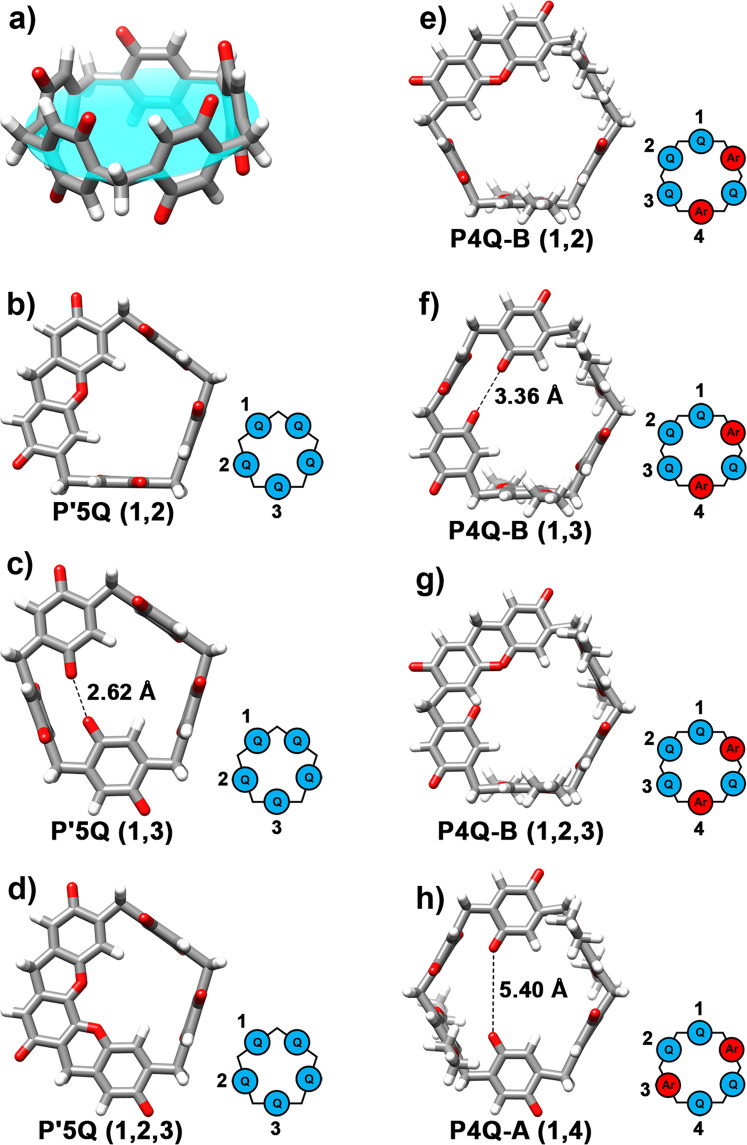
Rotating quinone effects. **a** Equatorial plane (in light blue) of P’5Q. **b**–**d** Conformations of maximum approach between rotating quinone units in P’5Q. **e**–**h** Conformations of maximum approach between rotating quinone units in P4Q-A.

Figure 7a shows the equatorial plane (in blue) of P’5Q in which the quinone units can approach via terminal oxygens as they rotate, enabling them to exchange electrons. As shown in Fig. 7h, communication through these equatorial pathways is spatially implausible for compounds possessing non-neighboring quinone units, which can only exchange electrons via peripheral pathways. It is important to point out that equatorial communication is important for adjacent quinone units in a macrocycle, (as shown in Fig. 7b, d, e, g) whereas the relevance of this mechanism between two nonadjacent quinone units is still at best minor, as shown in Fig. 7c, f.

To further validate the various electronic communication mechanisms, either peripheral or equatorial, we decided to carry out DFT computational studies on the macrocycles and their various reduced states during the course of their multi-electronic reduction processes. Following the findings in our previous work^14^, we used the *ωB*97*X* functional, which not only includes a suitable trade-off between a reduction of the self-interaction error and a partial conservation of non-dynamical correlation contributions, but also produces the least amount of spin contamination for the structures optimized at this level. As we did in our previous study, the wave function stability results for the macrocycles at their neutral state proves the stability under the closed shell (singlet state) regime, while the corresponding reduced states are stable at their highest spin multiplicity using an unrestricted approach.

A general trend that is clearly demonstrated by our electrochemical data is that the reduction potentials shift to less negative values as the number of quinone units in the macrocycle increases. This phenomenon is clearly the result of the increasing electron acceptor character (i.e., electron affinity) that the macrocycles acquire as the ratio of quinone-to-aromatic units increases. Figure 6c shows this trend clearly in graphical form. Considering the negative value of the LUMO energy as the electron affinity parameter defined by Koopman’s theorem allows us to estimate the extent of decrease in the LUMO energy of the surveyed macrocycles as the number of electron-deficient quinones increases. Our computational results nicely confirm that the electron affinity of the macrocyclic compounds increases as electron-poor quinone units replace the electron-rich aromatic units. Figure 6d shows the plot of the first half-wave potential of the macrocycles versus the LUMO energy of their neutral forms yielding the correlation coefficient value of 0.94 obtained at *ωB*97*X* level.

**Fig. 8:**
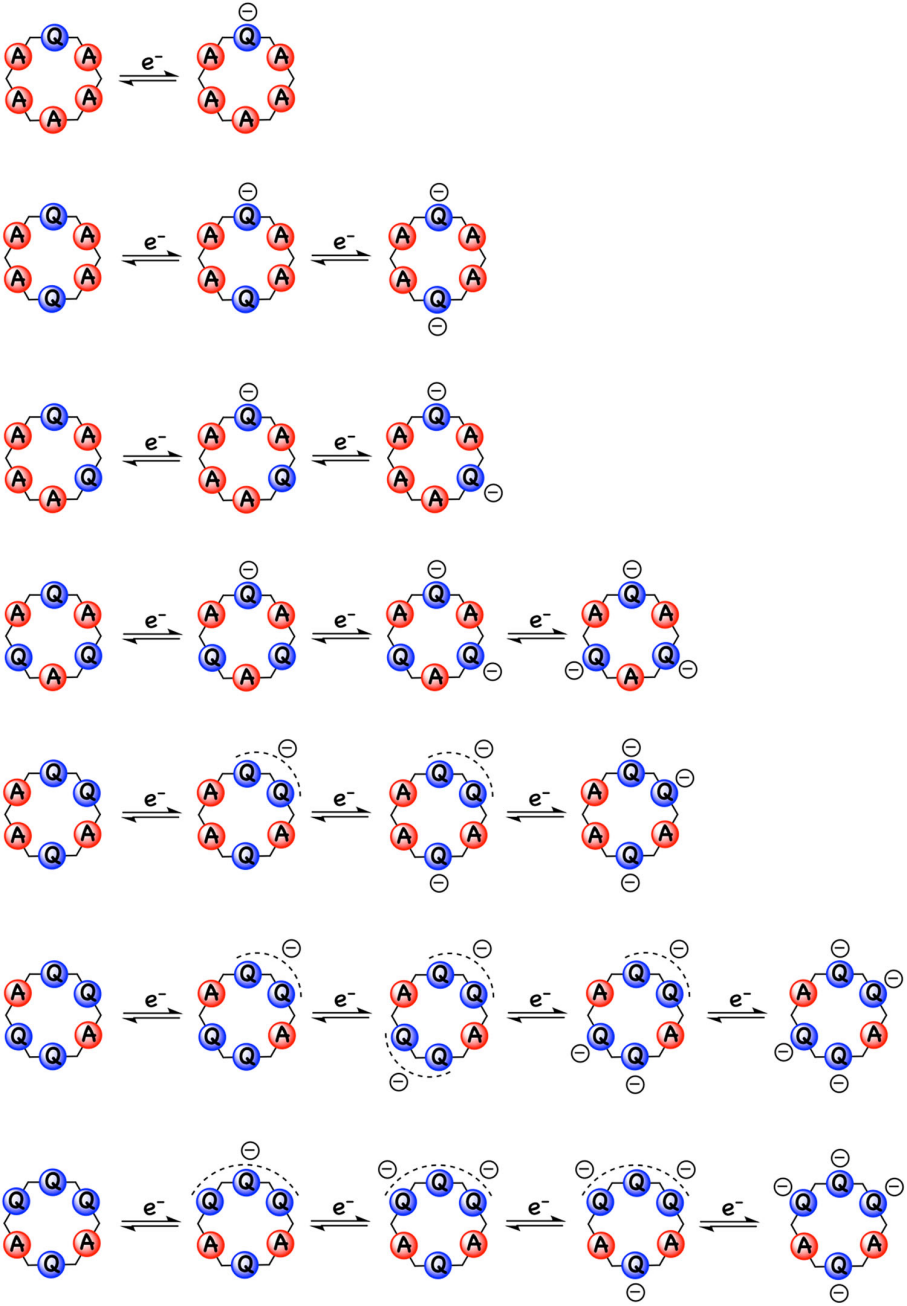
Individual reduction steps. Mechanism for the stepwise reduction of each of the surveyed quinone-containing pillar[6]arenes.

**Fig. 9:**
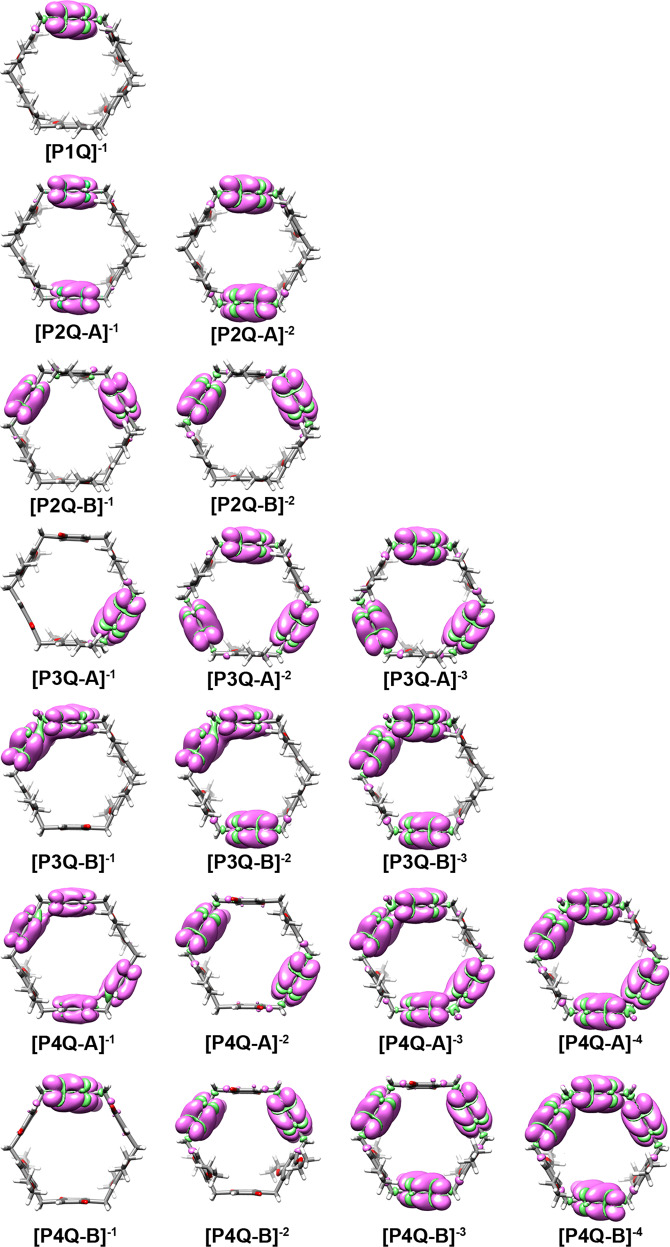
Electron distribution for each of the reduced states. Iso-density plots of the subtract of alpha (pink) and beta (green) spin densities for different reduced state of P1Q, P2Q-A, P2Q-B, P3Q-A, P3Q-B, P4Q-A, and P4Q-B compounds. The overlap between densities over adjacent quinone units confirms the presence of through-space communication between them.

Figure 8 shows the stepwise reduction mechanisms that we postulate for each of the macrocycles based on our electrochemical data and previous reports. To support our proposed mechanism, the electron distribution after each step was calculated by subtracting the spin densities of *β* electrons from those of *α* ones as shown in Fig. 9.

In addition to great consistency between the obtained computational results and the proposed stepwise reduction mechanisms, resonance sharing of one electron between two quinone groups is observed in some cases, emphasizing the proposed peripheral through-space communication between adjacent quinone residues.

In conclusion, we have shown that the oxidation of 1,4-diethoxypillar[6]arene with CAN yields macrocycles containing from one to four benzoquinone units, depending on the experimental conditions. We could not isolate macrocycles containing five or six quinone residues, although we detected, using mass spectrometric experiments, trace amounts of P5Q and P6Q in some of our reaction mixtures. In general terms, macrocycles with an accumulation of adjacent quinone units were disfavored and those with alternate quinones and aromatic residues were preferred. Thus, we could not isolate the C isomers (see Fig. 1) of P2Q, P3Q, and P4Q, and we only obtained one macrocycle (P4Q-B) with three adjacent quinone residues. We solved the crystal structures of P1Q, P2Q-A, and P3Q-A. (P1Q is a known compound, but we report here for the first time its X-ray crystal structure and electrochemical properties.) There are strong similarities between the structures of P1Q and P3Q-A, while the structure of P2Q-A is different. These differences seem to stem primarily from the presence of different solvent molecules in the crystal lattices, which arise from the different crystallization conditions. Finally, the cathodic electrochemical behavior of these macrocycles, generally resembles that of quinones. In compounds containing more than one quinone unit, the electronic communication between quinone residues is only substantial when they are directly connected to a bridging methylene carbon. In this sense, the electrochemical behavior of the pillar[*n*]quinone[6-*n*]arenes is relatively similar to that of pillar[*n*]quinone[5-*n*]arenes, at least for *n* = 1–4. We identified and discussed two types of electronic communication in these compounds: Peripheral, as quantified by computed ACID, and equatorial, based on the rotations of the quinone units. Finally, it is important to point out that the new compounds reported here can be easily functionalized, because the 1,4-benzoquinone (BQ) groups can be readily reduced to hydroquinones, to which other functional groups or molecular components can be appended via standard Williamson reactions. Furthermore, the host binding properties of these redox-active macrocycles are expected to be more interesting than those of oxidized pillar[5]arene derivatives, since the cavity sizes of the latter limit considerably the guest types that can be effectively bound. This matter will be the subject of future work.

## Methods

### Computational details

The Gaussian 09 program package (version E.01)^27^ was used to optimize all structures at two different functionals of M062X (global meta-hybrid) and *ω*B97X (range-separated hybrid) either in vacuum, for X-ray analysis purposes, or in the presence of solvent, which resembles the real experimental conditions, as a continuous and uniform dielectric medium, which is characterized by its dielectric constant *ε*. 6–31 + G(d,p) basis set possessing diffuse functions is suitable to treat anionic moieties with delocalized electrons. The solvent is introduced implicitly with the self-consistent reaction field (SCRF) theory using the integral equation formalism model (IEFPCM) which creates an embracing cavity by interlocking spheres around the solute^28^. To guarantee the accuracy and stability of all numerical computation processes, we employed an ultrafine integration grid (99590) without imposing any symmetry on structures. Additionally, the examination of frequency calculations of structures at their corresponding level of theory confirmed that all structures were located on a local minimum. The iso-value for presentation of the difference between alpha and beta densities is about 0.0004 electron/bohr. Anisotropy of the current (induced) density (ACID) calculations were performed at the level of M062X/6–31 + G(d,p) using ACID.2.0.0 program^22^.

### General experimental details

All NMR spectra were recorded on 500 MHz or 400 MHz spectrometers. Mass spectra were acquired on a MicroQ-TOF ESI mass spectrometer (Supplementary Figs. 40–46). Electrochemical experiments were carried out on a CH Instruments model 900 system, using a single-compartment cell, fitted with a glassy carbon working electrode, a platinum wire serving as auxiliary electrode and a Ag/AgCl/KCl (3.5 M) reference electrode. All potentials are reported against this reference electrode. Voltammetric data were obtained after thorough purging of the test solution with purified nitrogen gas.

### Synthesis

To synthesize per-ethoxypillar[6]arene (P6A) in large scale, we followed Cao’s procedure (See Supplementary Fig. 1 for NMR data)^19^. A solution of cerium(IV) ammonium nitrate (CAN) (1.03 mmoles, 2.2 equiv, dissolved in 10 mL water) was added dropwise into the solution of P6A (0.5 g, 4.68 × 10^−4^ mol) in 50 mL of CH_2_Cl_2_/THF (1:1) and the reaction mixture was stirred at room temperature for 20 h to obtain P1Q compound with a yield of 0.346 g (73.2%). For synthesis of P2Q compounds, 10 mL aqueous solution containing 4.4 equiv of CAN (2.06 mmol) was added dropwise into the solution of P6A (0.5 g, 4.68 × 10^−4^ mol) in 100 ml of CH_2_Cl_2_/THF (1:1) and stirred for 18 h to obtain P2Q-A and P2Q-B with yields of 0.276 g (62%) and 0.103 g (23.1%), respectively. P3Q-A and P3Q-B with yields of 0.272 g (65.1%) and 0.089 g (21.2%), respectively, were obtained by gently adding 15 mL aqueous solution of CAN (3.08 mmol, 6.6 equiv) into the stirring solution of P6A (0.5 g, 4.68 × 10^−4^ mol) in 150 mL of CH_2_Cl_2_/THF (1:1) solution and allowing the reaction to run for 18 h. For the synthesis of P4Q isomers, 20 mL aqueous solution containing 8.8 equiv of CAN (4.11 mmol) was added dropwise into the solution of P6A (0.5 g, 4.68 × 10^−4^ mol) in 200 mL of CH_2_Cl_2_/THF (1:1) and stirred for 18 h to obtain P4Q-A and P4Q-B compounds with yields of 0.162 g (41.3%) and 0.072 g (18.5%), respectively. As discussed in the literature^29^, it is of great importance to run the reactions under an inert argon atmosphere to prevent the oxidation of the reduced cerium. All reaction mixtures after completion were washed with water (3 × 100 mL) and concentrated under reduced pressure. The residue was subjected to chromatography (silica gel, CH_2_Cl_2_:hexane 10:1), to isolate the final compounds.

*P1Q*: ^1^H NMR (500 MHz, CDCl_3_) δ 6.77–6.59 (m, 10H), 6.43 (s, 2H), 3.95–3.72 (m, 28H), 3.57 (d, *J* = 1.4 Hz, 4H), 1.40–1.18 (m, 30H). ^13^C NMR: (101 MHz, CDCl_3_) δ 188.3, 150.6, 150.5, 150.4, 146.6, 133.6, 129.4, 128.3, 128.0, 127.3, 122.7, 115.6, 115.3, 115.2, 114.6, 64.2, 64.1, 64.0, 63.6, 31.0, 30.6, 30.4, 15.2, 15.1, 15.0. HRMS (ESI) *m/z* [P1Q•Na^+^] calcd for C_62_H_74_O_12_Na 1033.5078, found 1033.5066 (error: 1.16 ppm).

*P2Q-A*: ^1^H NMR (500 MHz, CDCl_3_) δ 6.68 (d, 8H), 6.38 (d, 4H), 3.93–3.78 (m, 20H), 3.59 (d, *J* = 1.5 Hz, 8H), 1.34 (t, 12H), 1.24 (t, 12H). ^13^C NMR: (101 MHz, CDCl_3_) δ 188.3, 150.7, 150.6, 146.9, 133.5, 128.9, 123.0, 115.2, 115.0, 64.2, 63.8, 31.3, 30.3, 15.1, 15.0. HRMS (ESI) *m/z* [P2Q-A•Na^+^] calcd for C_58_H_64_O_12_Na 975.4295, found 975.4330 (error: 3.59 ppm).

*P2Q-B*: ^1^H NMR (500 MHz, CDCl_3_) δ 6.72–6.67 (m, 6H), 6.62–6.55 (m, 4H), 6.38–6.37 (m, 2H), 3.97–3.74 (m, 20H), 3.58 (dd, *J* = 8.9, 1.3 Hz, 8H), 1.30 (ddt, *J* = 41.8, 30.2, 7.0 Hz, 24H). ^13^C NMR: (101 MHz, CDCl_3_) δ 188.2, 188.1, 150.7, 150.6, 150.5, 146.9, 146.1, 134.1, 133.6, 129.6, 127.7, 124.2, 122.9, 115.4, 115.3, 114.8, 114.6, 64.3, 64.1, 63.9, 63.7, 30.6, 30.5, 30.0, 15.1, 15.0, 14.9. HRMS (ESI) *m/z* [P2Q-B•Na^+^] calcd for C_58_H_64_O_12_Na 975.4295, found 975.4336 (error: 4.20 ppm).

*P3Q-A*: ^1^H NMR (500 MHz, CDCl_3_) δ 6.67 (s, 6H), 6.51 (d, *J* = 1.3 Hz, 6H), 3.90 (q, *J* = 7.0 Hz, 12H), 3.60 (d, *J* = 1.3 Hz, 12H), 1.32 (t, *J* = 6.9 Hz, 18H). ^13^C NMR: (101 MHz, CDCl_3_) δ 188.0, 150.6, 146.5, 134.0, 124.5, 114.7, 64.0, 30.0, 15.0. HRMS (ESI) *m/z* [P3Q-A•Na^+^] calcd for C_54_H_54_O_12_Na 917.3513, found 917.3545 (error: 3.49 ppm).

*P3Q-B*: ^1^H NMR (500 MHz, CDCl_3_) δ 6.74–6.62 (m, 6H), 6.60–6.55 (m, 3H), 6.51–6.27 (m, 3H), 3.97–3.81 (m, 14H), 3.63–3.57 (m, 8H), 3.44 (t, *J* = 1.3 Hz, 2H), 1.40–1.20 (m, 18H). ^13^C NMR: (101 MHz, CDCl_3_): ^13^C NMR (101 MHz, CDCl_3_) δ 188.3, 187.4, 187.2, 187.1, 150.8, 150.7, 150.5, 147.8, 147.1, 146.9, 146.1, 144.0, 143.9, 135.2, 134.9, 134.1, 133.7, 133.6, 133.3, 129.2, 129.1, 124.5, 123.8, 123.0, 122.6, 115.3, 115.1, 114.9, 64.3, 64.2, 64.0, 63.9, 63.8, 30.8, 30.5, 30.3, 30.1, 29.9, 29.2, 15.1, 15.0, 14.9. HRMS (ESI) *m/z* [P3Q-B•Na^+^] calcd for C_54_H_54_O_12_Na 917.3513, found 917.3547 (error: 3.71 ppm).

*P4Q-A*: ^1^H NMR (500 MHz, CDCl_3_) δ 6.71 (s, 4H), 6.59–6.55 (m, 4H), 6.49–6.45 (m, 4H), 3.94 (q, *J* = 7.0 Hz, 8H), 3.62 (d, *J* = 1.3 Hz, 8H), 3.45 (d, *J* = 1.4 Hz, 4H), 1.34 (t, *J* = 6.9 Hz, 12H). ^13^C NMR: (101 MHz, CDCl_3_) δ 187.2, 150.7, 147.1, 144.1, 135.1, 133.7, 124.2, 115.0, 64.0, 30.0, 29.2, 15.0. HRMS (ESI) *m/z* [P4Q-A•Na^+^] calcd for C_50_H_44_O_12_Na 859.2730, found 859.2742 (error: 1.40 ppm)

*P4Q-B*: ^1^H NMR (500 MHz, CDCl_3_) δ 6.71 (d, *J* = 1.7 Hz, 4H), 6.66 (d, *J* = 1.3 Hz, 2H), 6.53 (t, *J* = 1.3 Hz, 2H), 6.50 (dd, *J* = 6.0, 1.3 Hz, 4H), 3.93 (dq, *J* = 13.9, 6.9 Hz, 8H), 3.61 (dd, *J* = 3.7, 1.3 Hz, 8H), 3.46 (t, *J* = 1.3 Hz, 4H), 1.34 (dt, *J* = 11.3, 6.9 Hz, 12H). ^13^C NMR: (101 MHz, CDCl_3_) δ 188.2, 187.1, 187.0, 186.2, 150.8, 150.7, 147.4, 146.4, 144.7, 143.4, 135.6, 134.5, 133.9, 133.7, 124.5, 124.1, 114.9, 64.0, 63.9, 30.1, 29.7, 28.8, 15.0. HRMS (ESI) *m/z* [P4Q-B•Na^+^] calcd for C_50_H_44_O_12_Na 859.2730, found 859.2698 (error: 3.72 ppm).

### Crystallographic analysis

Crystals of P1Q and P3Q-A suitable for single-crystal X-ray diffraction analysis were grown by slow evaporation of a chloroform/dioxane solvent mixture at room temperature. Single crystals of P2Q-A suitable for diffraction analysis were grown by slow evaporation of a methylene chloride/benzene solvent mixture at 7 °C. The data crystals for P1Q and P2Q-A were mounted onto the end of a thin glass fiber using Paratone-N for data collection at 100 K under N_2_. The data crystal for P3Q-A were glued onto the end of a thin glass fiber for data collection at room temperature. X-ray intensity data were measured by using a Bruker SMART APEX2 CCD-based diffractometer using Mo K*α* radiation (λ = 0.71073 Å)^30^. The raw data frames were integrated with the SAINT+ program by using a narrow-frame integration algorithm^30^. Corrections for Lorentz and polarization effects were also applied with SAINT+. An empirical absorption correction based on the multiple measurement of equivalent reflections was applied using the program SADABS. All structures were solved by a combination of direct methods and difference Fourier syntheses, and refined by full-matrix least-squares on F^2^, by using the SHELXTL software package^31,32^. All non-hydrogen atoms were refined with anisotropic displacement parameters. Hydrogen atoms were placed in geometrically idealized positions and included as standard riding atoms during the least-squares refinements. Crystal data, data collection parameters, and results of the analyses are listed in Supplementary Table 6. The corresponding CIFs are in Supplementary Data files 1–3. Compound P1Q crystallized in the triclinic crystal system. The space group *P* $\bar 1$ was assumed and confirmed by the successful refinement of the structure. One molecule of chloroform and one and a half molecules of dioxane from the solvent of crystallization co-crystallized with the P1Q macrocycle. One molecule of dioxane resides in the center of the pillararene cavity, severely disordered, and was refined with isotropic thermal parameters. There is no disorder present in the non-solvent part of the molecule. Compound P2Q-A crystallized in the triclinic crystal system. The space group *P* $\bar 1$ was assumed and confirmed by the successful refinement of the structure. Three molecules of benzene from the solvent of crystallization co-crystallized with the P2Q-A macrocycle. With *Z* = 1, the complex is crystallographically centrosymmetric. Compound P3Q-A crystallized in the trigonal crystal system. The space groups *R*3 *c* and *R*$\bar 3$ *c* were consistent with the systematic absences in the intensity data. The structure could only be solved in the former space group. P3Q-A has crystallographic 3-fold symmetry. One and a half molecules of chloroform from the crystallization solvent co-crystallized with the P2Q-A macrocycle. The chloroform molecule inside the pillararene cavity is disordered over two positions and was refined with 50% site occupancy.

## Supplementary information

Supplementary Information

Description of Additional Supplementary Files

Supplementary Data 1

Supplementary Data 2

Supplementary Data 3

Peer Review Files

## Data Availability

Most of the datasets generated or analyzed during this study are included in its published article (and its supplementary information files). The X-ray crystal structures of P1Q, P2Q-A, and P3Q-A have been deposited to the CCDC database (deposition numbers 1994722, 1994723, and 1994724, respectively) and are available as Supplementary Data 1–3. Any other datasets are available from the corresponding author on reasonable request.
