# Supplementary material for: Visualization and quantitation of electronic communication pathways in a series of redox-active pillar[6]arene-based macrocycles
Source: Commun Chem. 2020 Aug 13;3:117. doi: 10.1038/s42004-020-00363-4 (PMC9814560; doi:10.1038/s42004-020-00363-4)
Supplement: Supplementary file 1 — Supplementary Information [file 42004_2020_363_MOESM1_ESM.pdf]

SUPPLEMENTARY INFORMATION

Visualization and Quantitation of Electronic Communication Pathways in a Series of Redox-Active Pillar[6]arene-Based Macrocycles

Mehdi Rashvand Avei, Sedigheh Etezadi, Burjor Captain and Angel E. Kaifer*

Department of Chemistry, University of Miami, Coral Gables, FL 33124, U.S.A.

akaifer@miami.edu

TABLE OF CONTENTS

	Page
^{1}HNMR spectra…………………………………………………………	S2-9
^{13}CNMR Spectra………………………………………………………...	S10-17
X-ray Crystal Structures and structural analysis…..……………………………	S18-27
Schematic representation of oxidized derivatives of P'5A…………………..	S28
UV-vis spectra of P'5A, 1,4-DMB, 1,4-DEB and XQ compounds …………	S28
First derivative of SWVs and semi-integral of CVs……………..…………	S29-32
Mass spectra and their simulations………………………………………..	S33-36
Additional data tables……………………………………………………..	S37-38
Table of the number of aromatic-quinone proximities……..……………..	S38
HOMO and LUMO energies of derivatives of P'5A and P6A………………	S39
Crystal data, data collection parameters, and results of the analyses……..	S40
Supplementary References……………………………………………………	S41

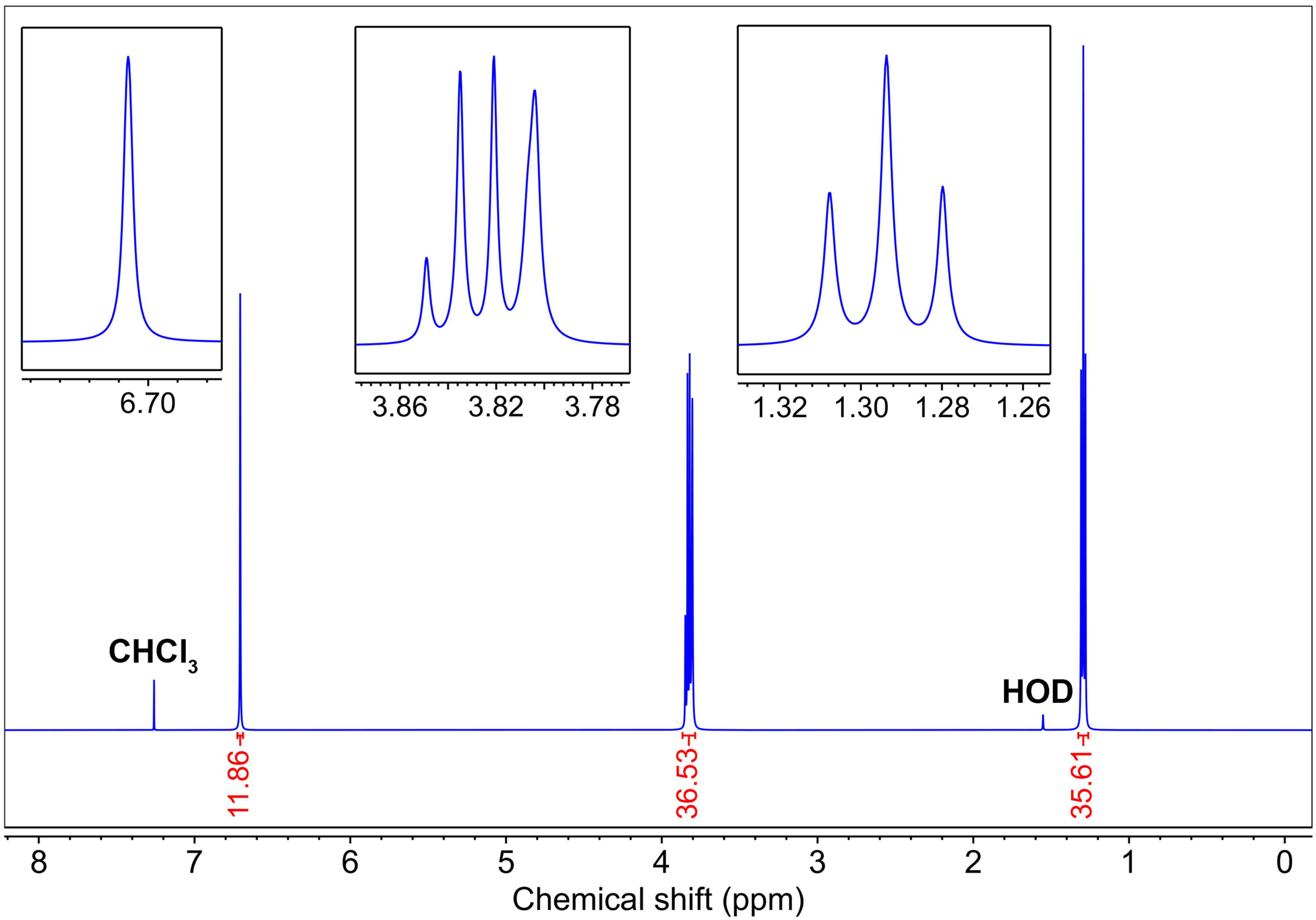

Supplementary Figure 1. ^{1}HNMR spectrum of P6A at 500 MHz in $CDCl_3$ at 25 ℃

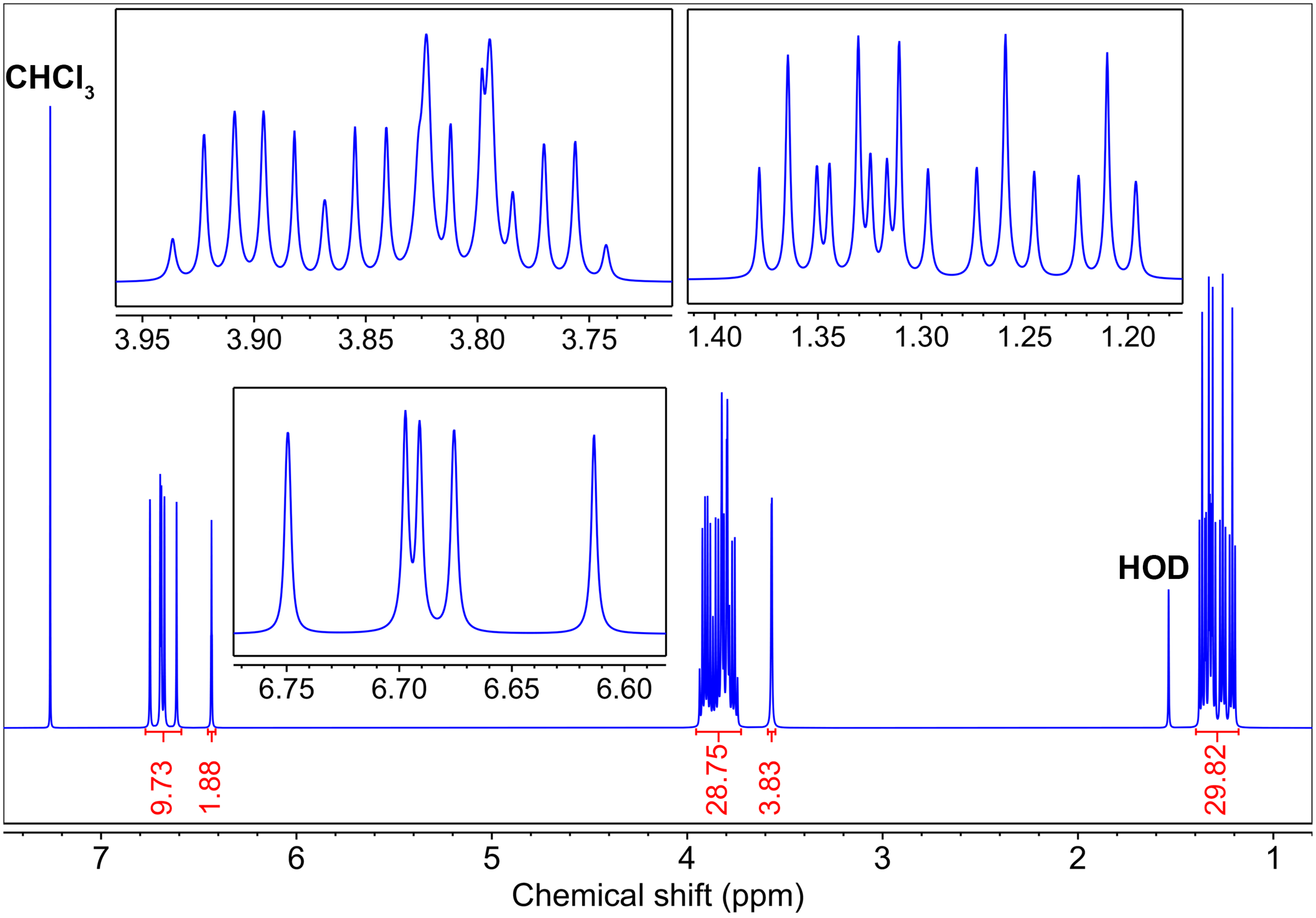

Supplementary Figure 2. ^{1}HNMR spectrum of P1Q at 500 MHz in $CDCl_3$ at 25 ℃

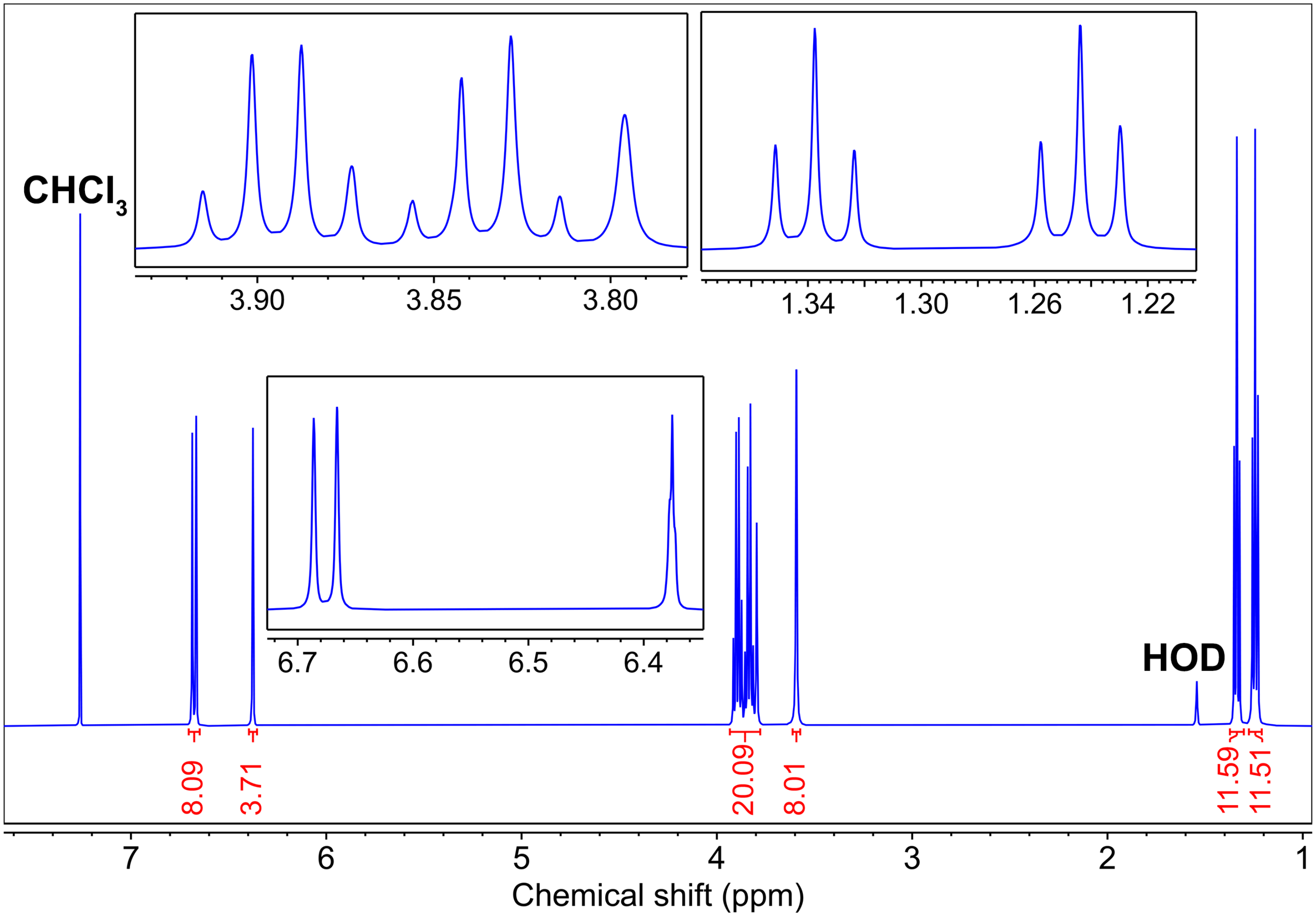

Supplementary Figure 3. ^{1}HNMR spectrum of P2Q-A at 500 MHz in $CDCl_3$ at 25 ℃

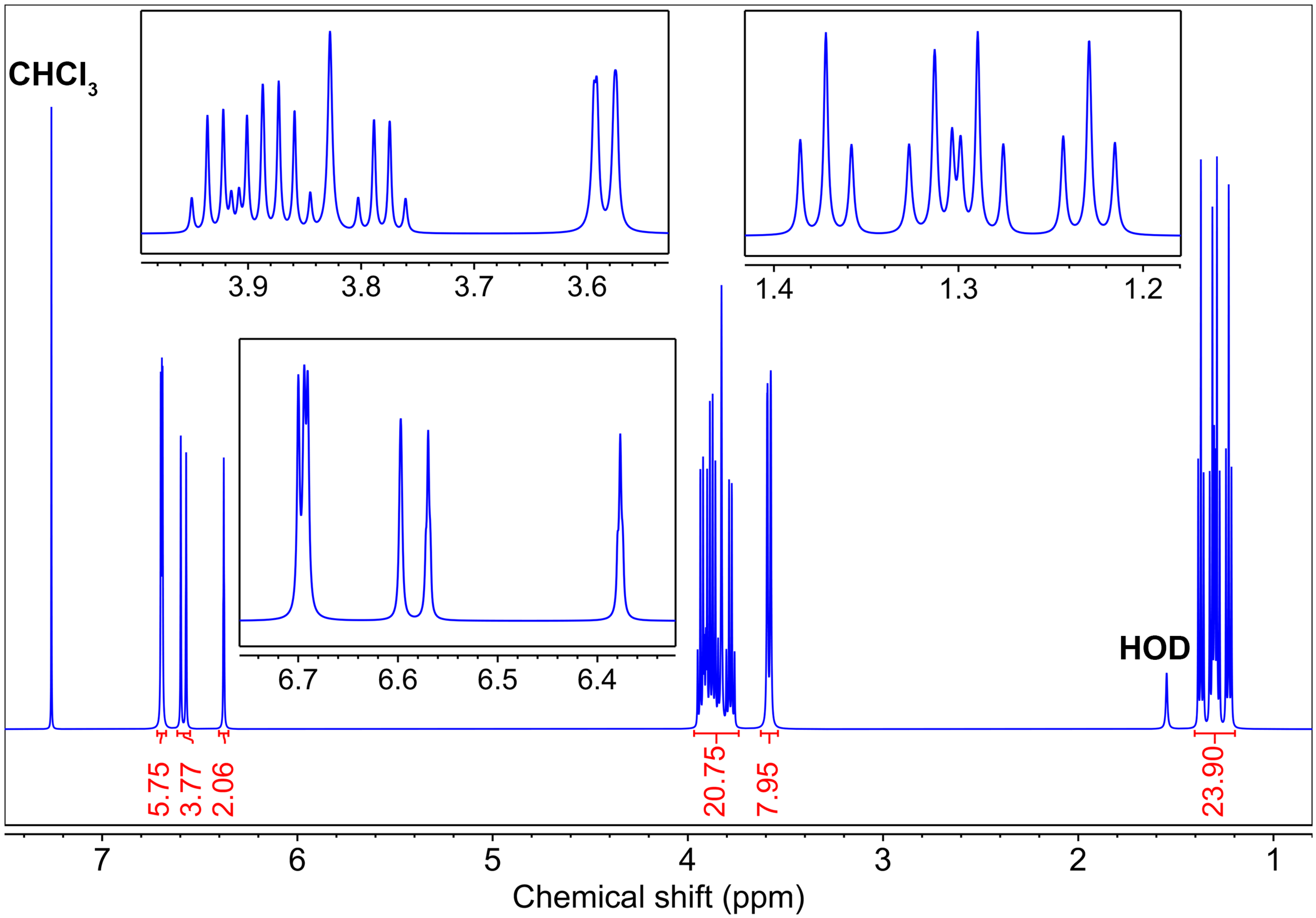

Supplementary Figure 4. ^{1}HNMR spectrum of P2Q-B at 500 MHz in $CDCl_3$ at 25 ℃

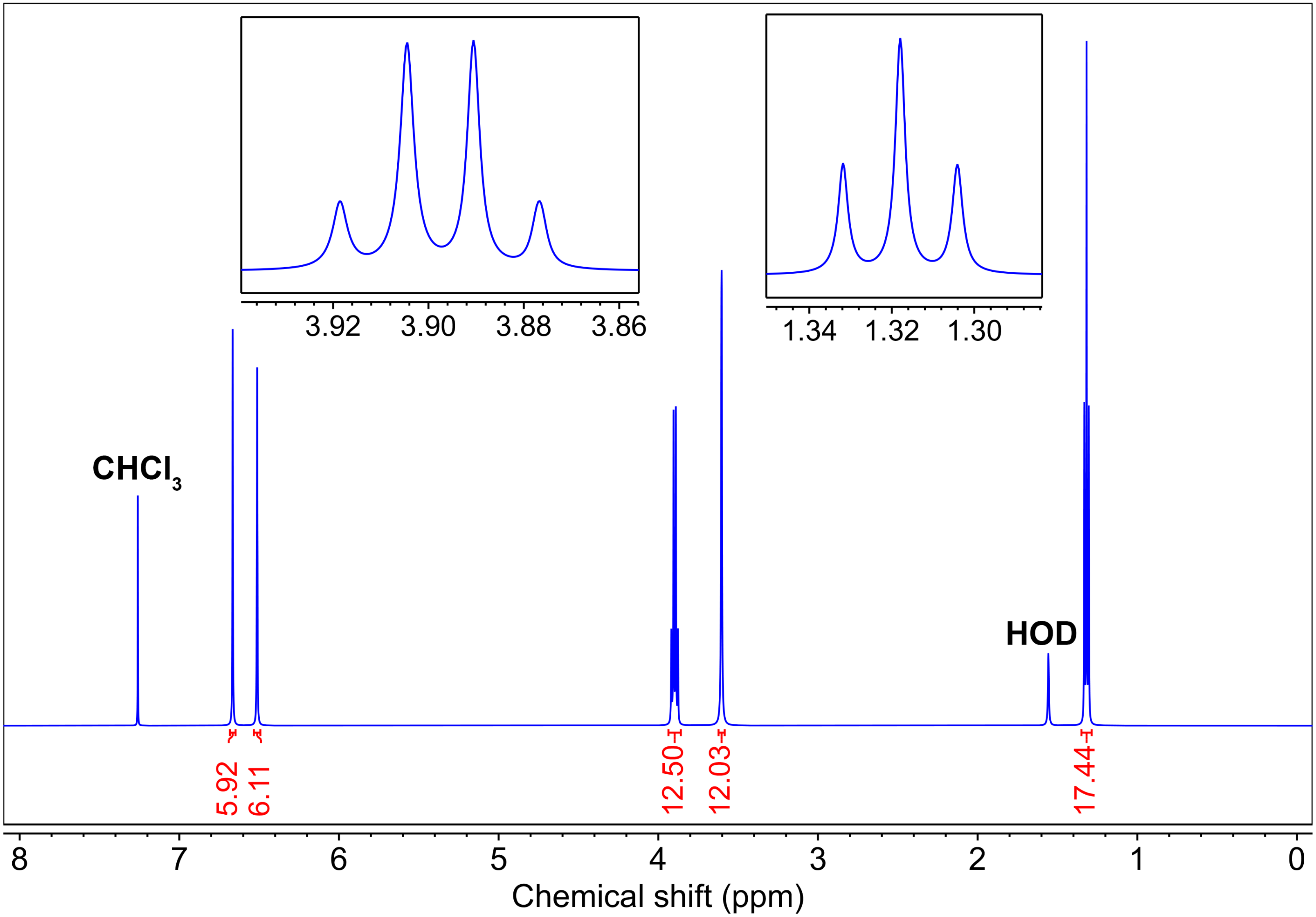

Supplementary Figure 5. ^{1}HNMR spectrum of P3Q-A at 500 MHz in $CDCl_3$ at 25 ℃

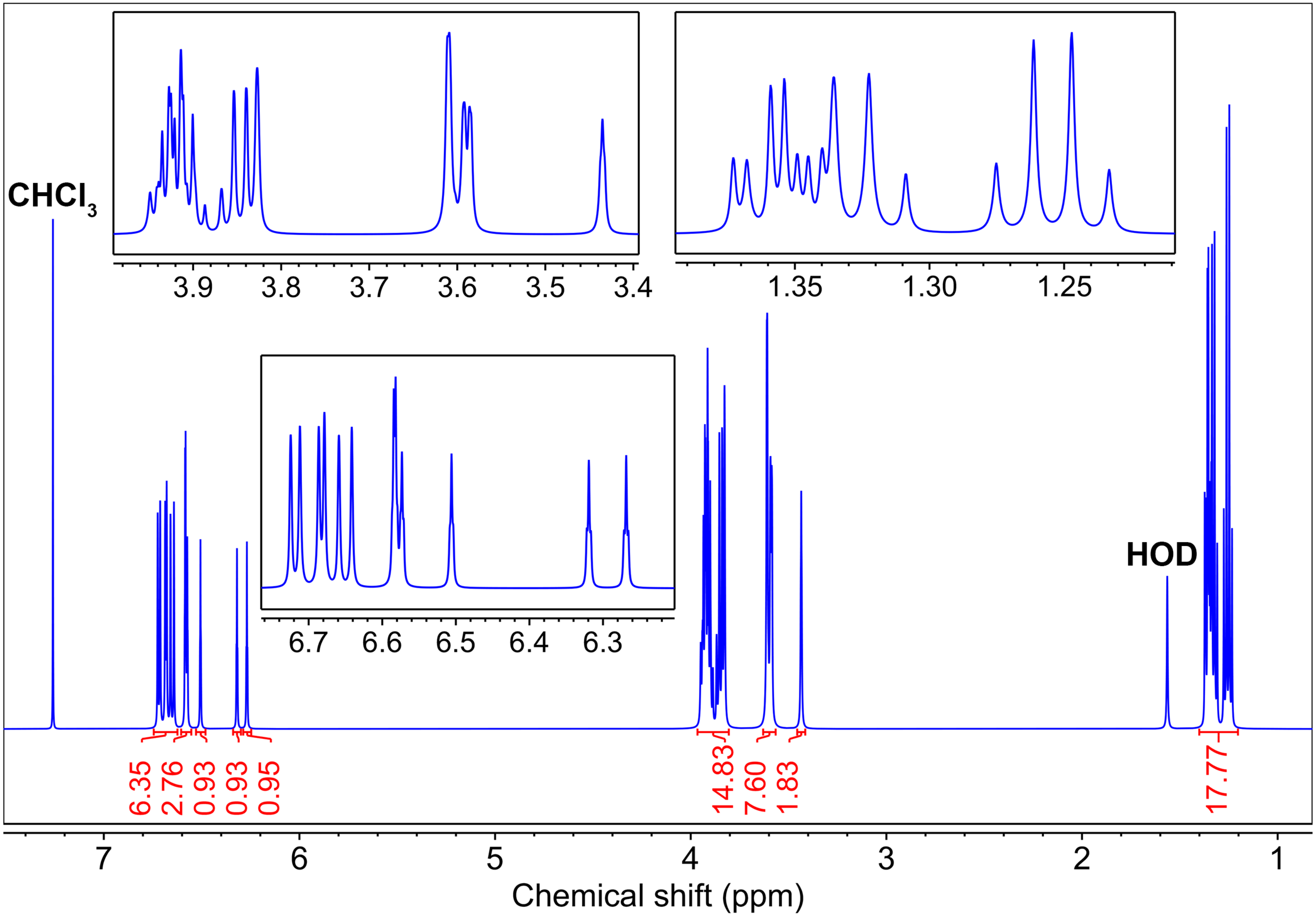

Supplementary Figure 6. ^{1}HNMR spectrum of P3Q-B at 500 MHz in $CDCl_3$ at 25 ℃

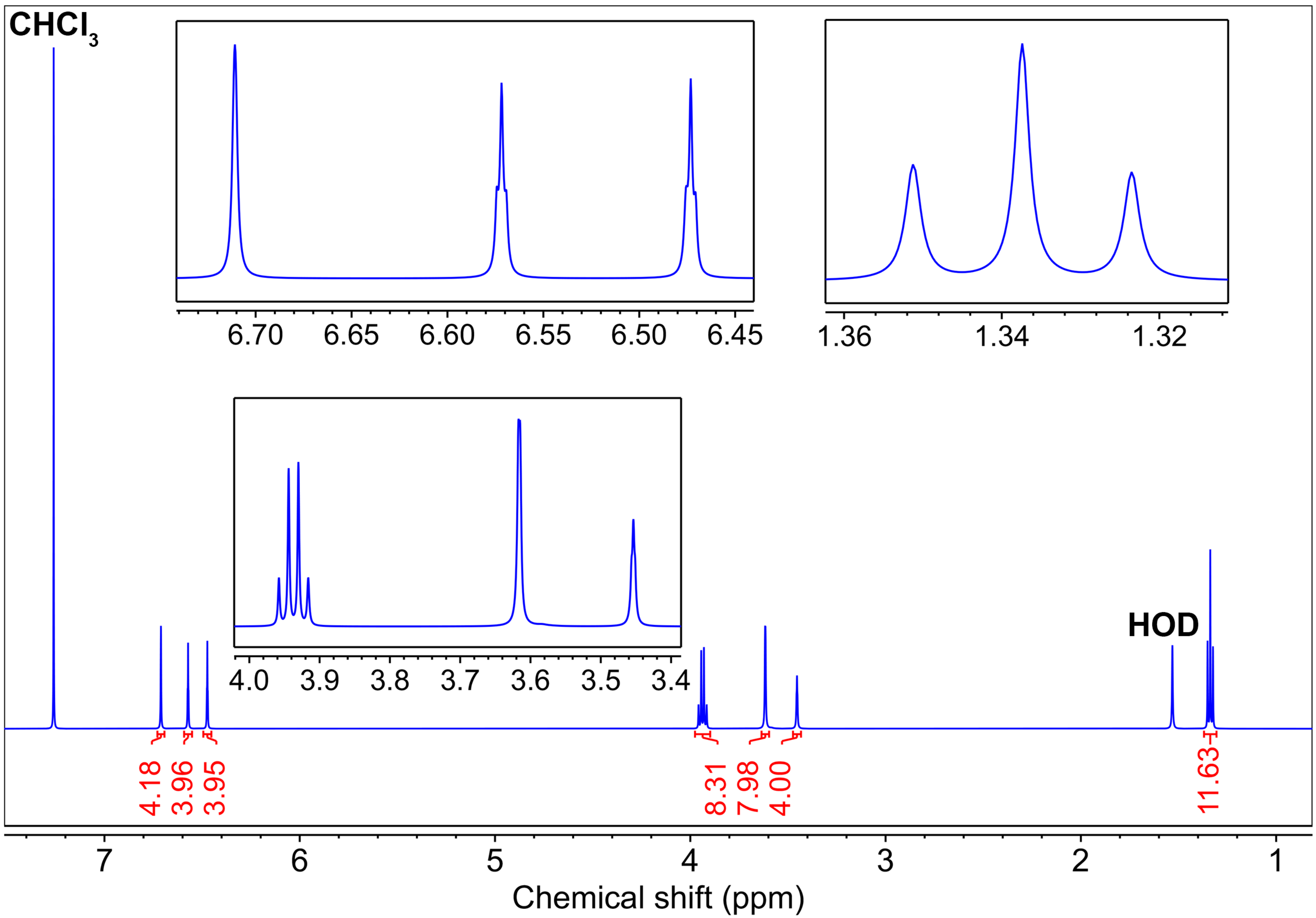

Supplementary Figure 7. ^{1}HNMR spectrum of P4Q-A at 500 MHz in $CDCl_3$ at 25 ℃

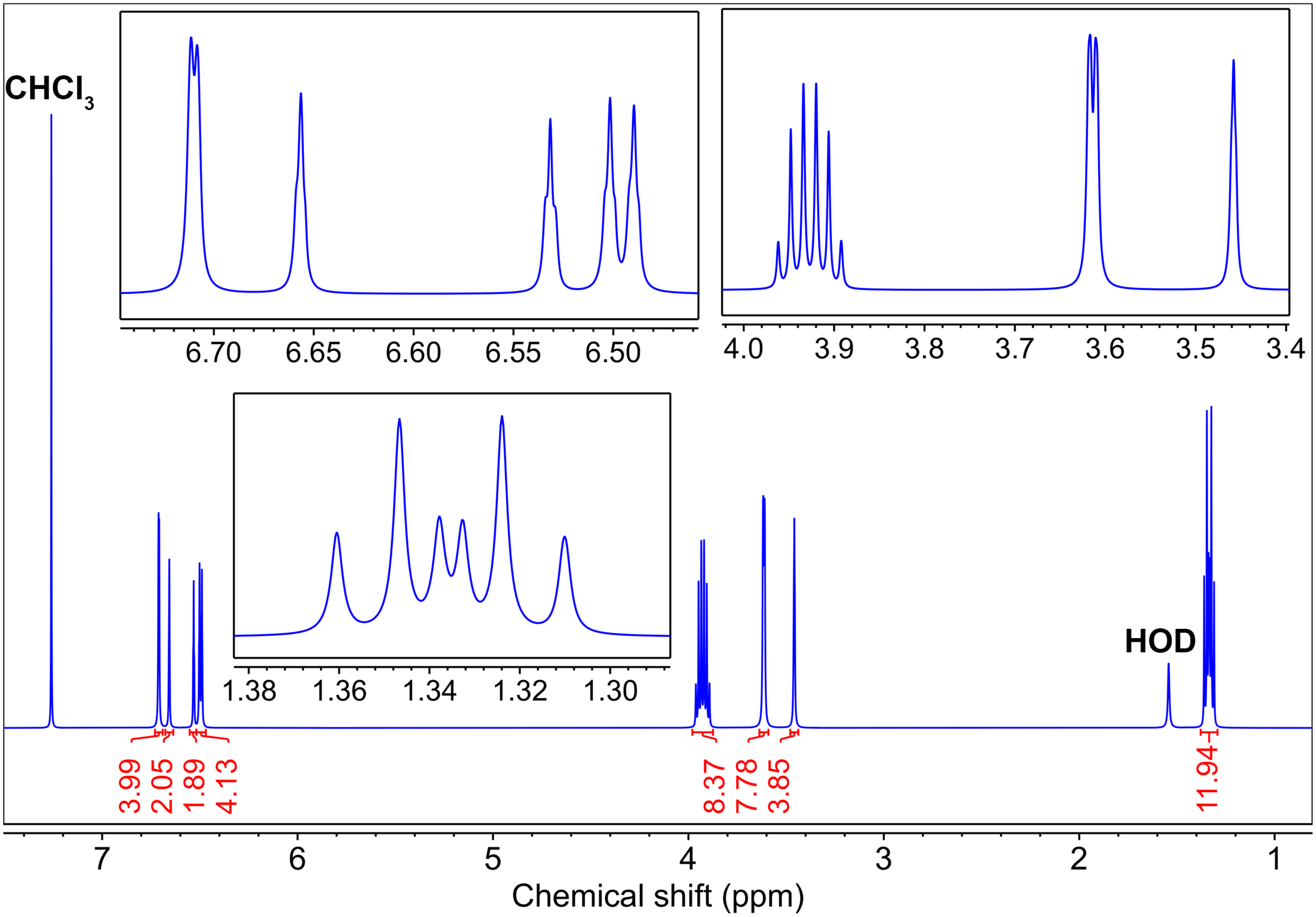

Supplementary Figure 8. ^{1}HNMR spectrum of P4Q-B at 500 MHz in $CDCl_3$ at 25 ℃

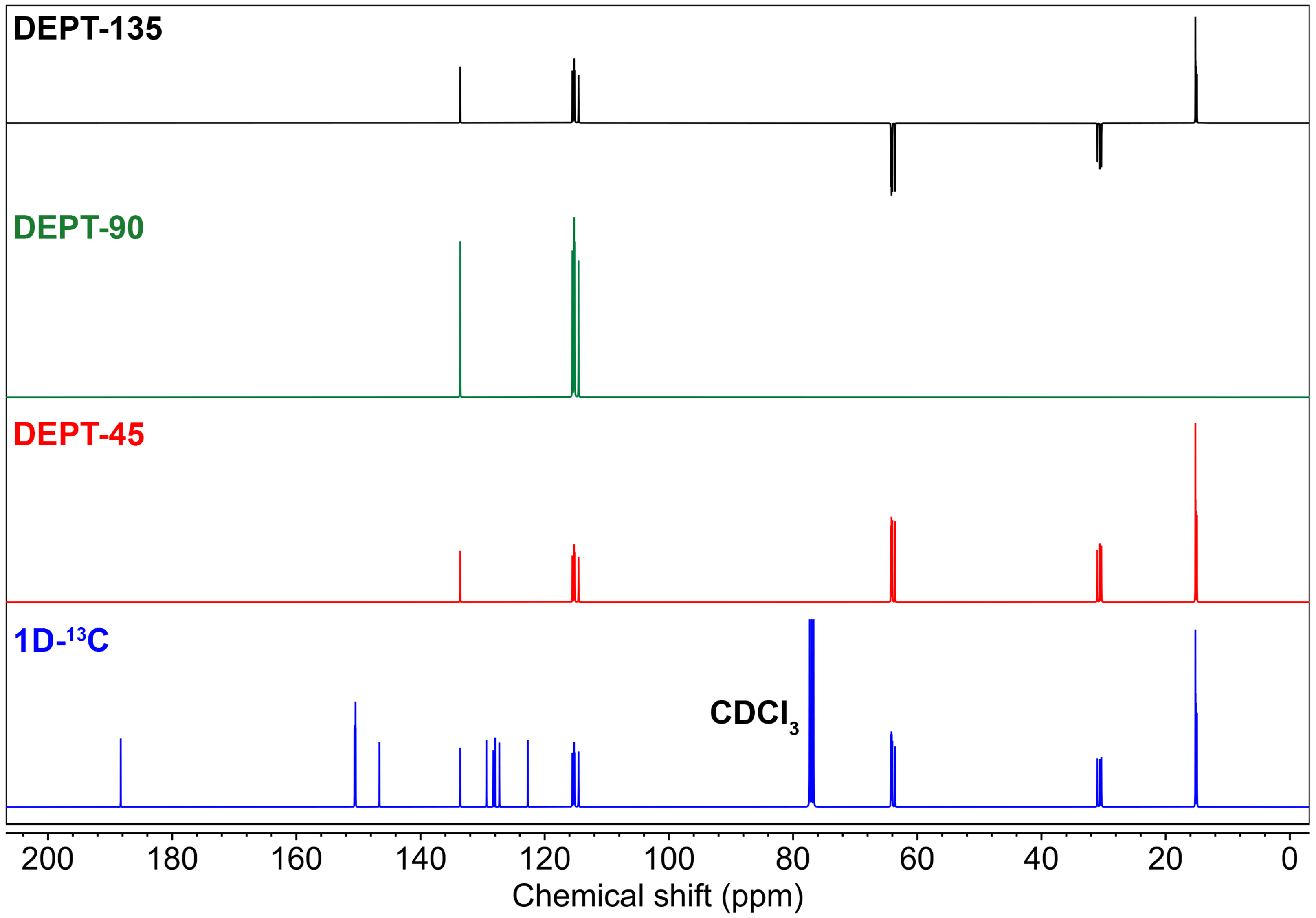

Supplementary Figure 9. 1D-^{13}CNMR and DEPT spectra of P1Q at 100 MHz in $CDCl_3$ at 25 ℃

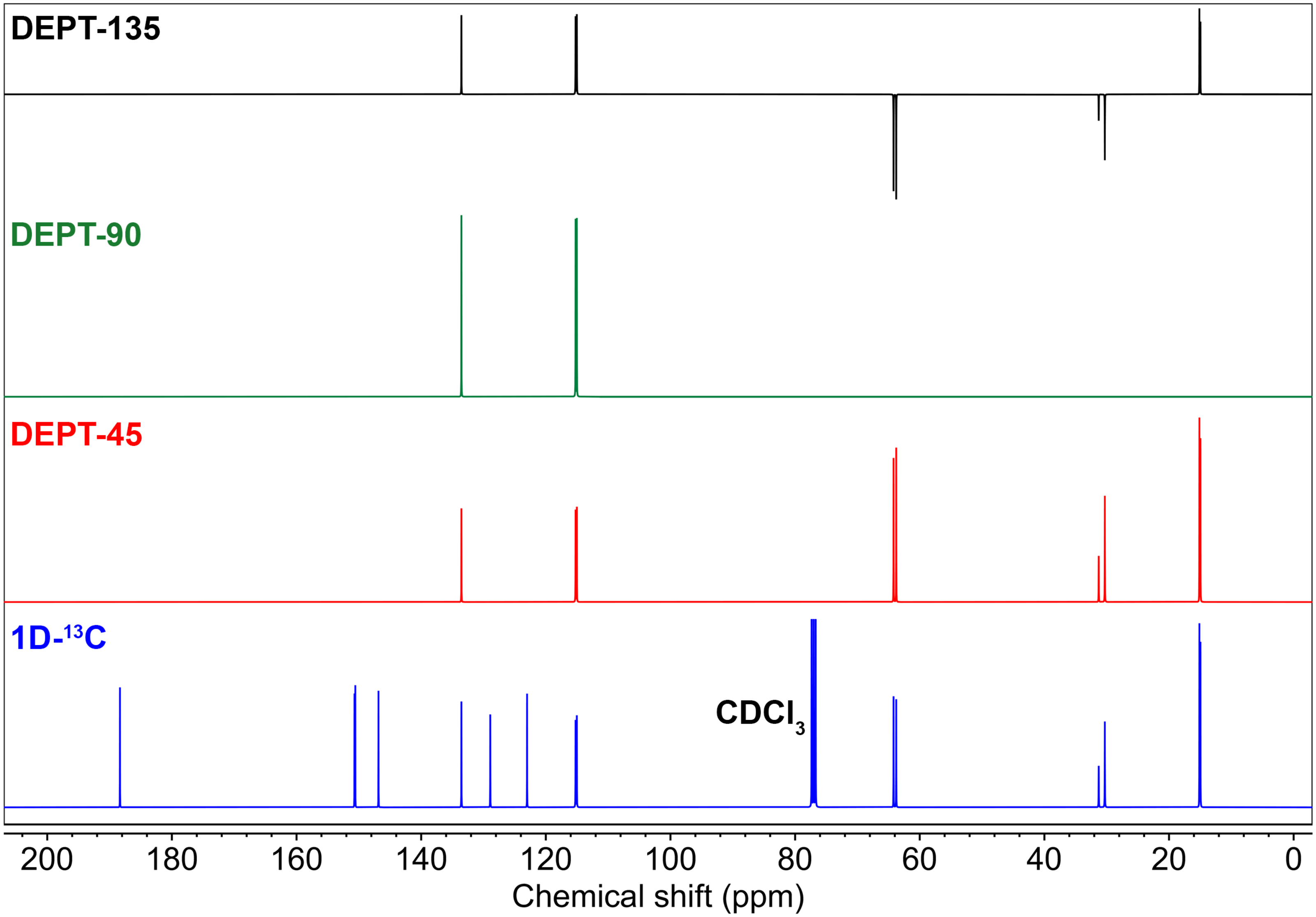

Supplementary Figure 10. 1D-^{13}CNMR and DEPT spectra of P2Q-A at 100 MHz in $CDCl_3$ at 25 ℃

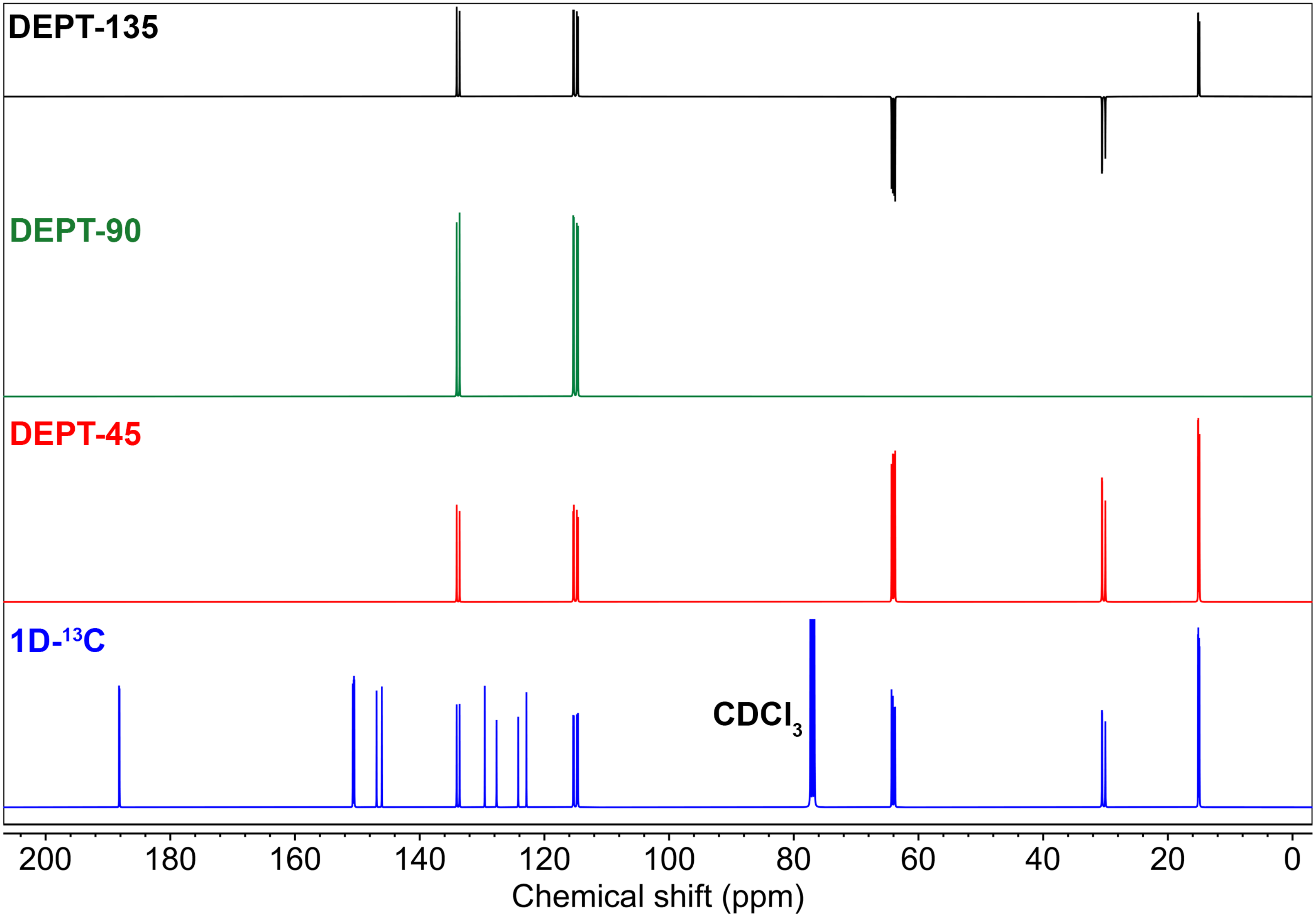

Supplementary Figure 11. 1D-^{13}CNMR and DEPT spectra of P2Q-B at 100 MHz in $CDCl_3$ at 25 ℃

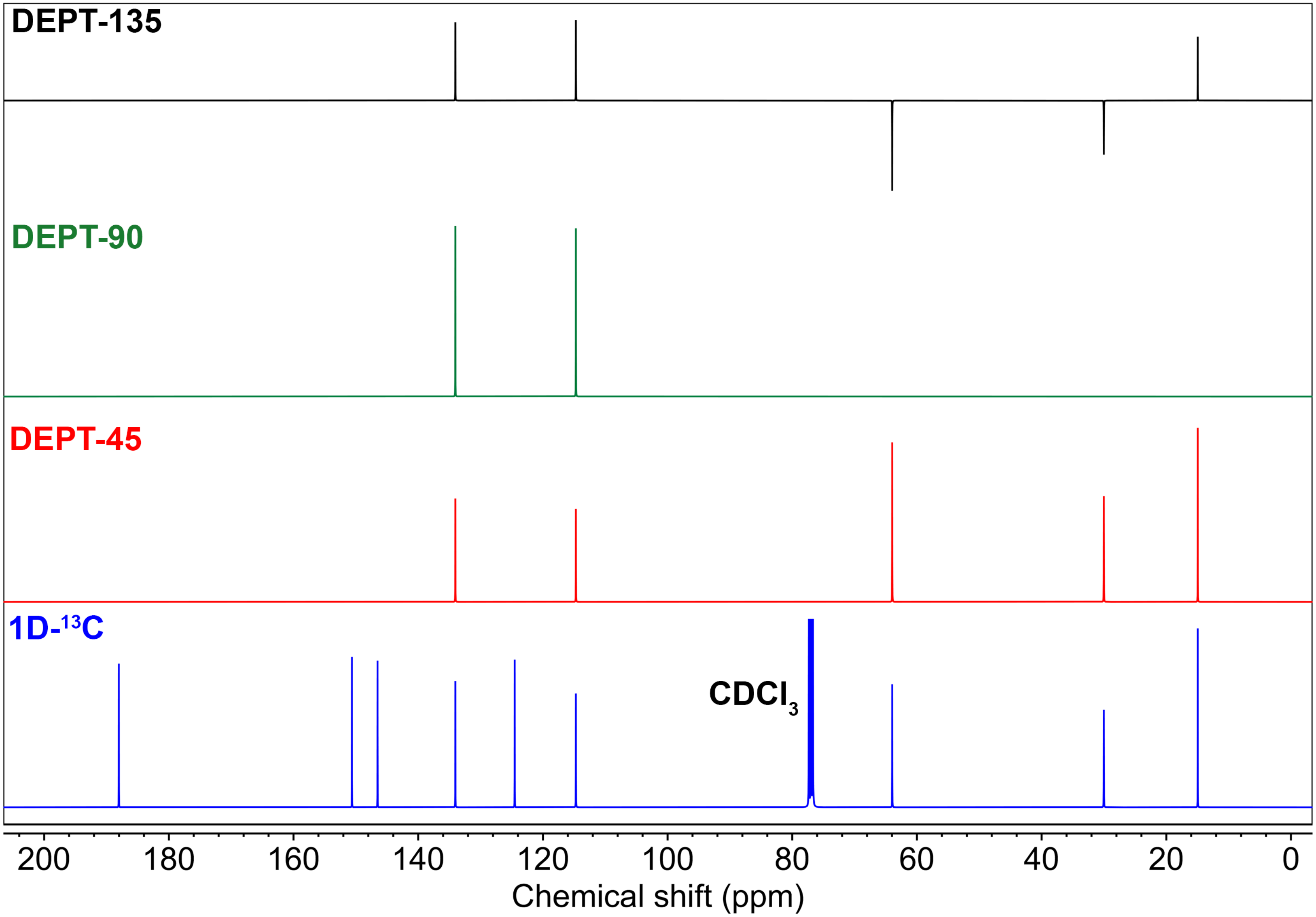

Supplementary Figure 12. 1D-^{13}CNMR and DEPT spectra of P3Q-A at 100 MHz in $CDCl_3$ at 25 ℃

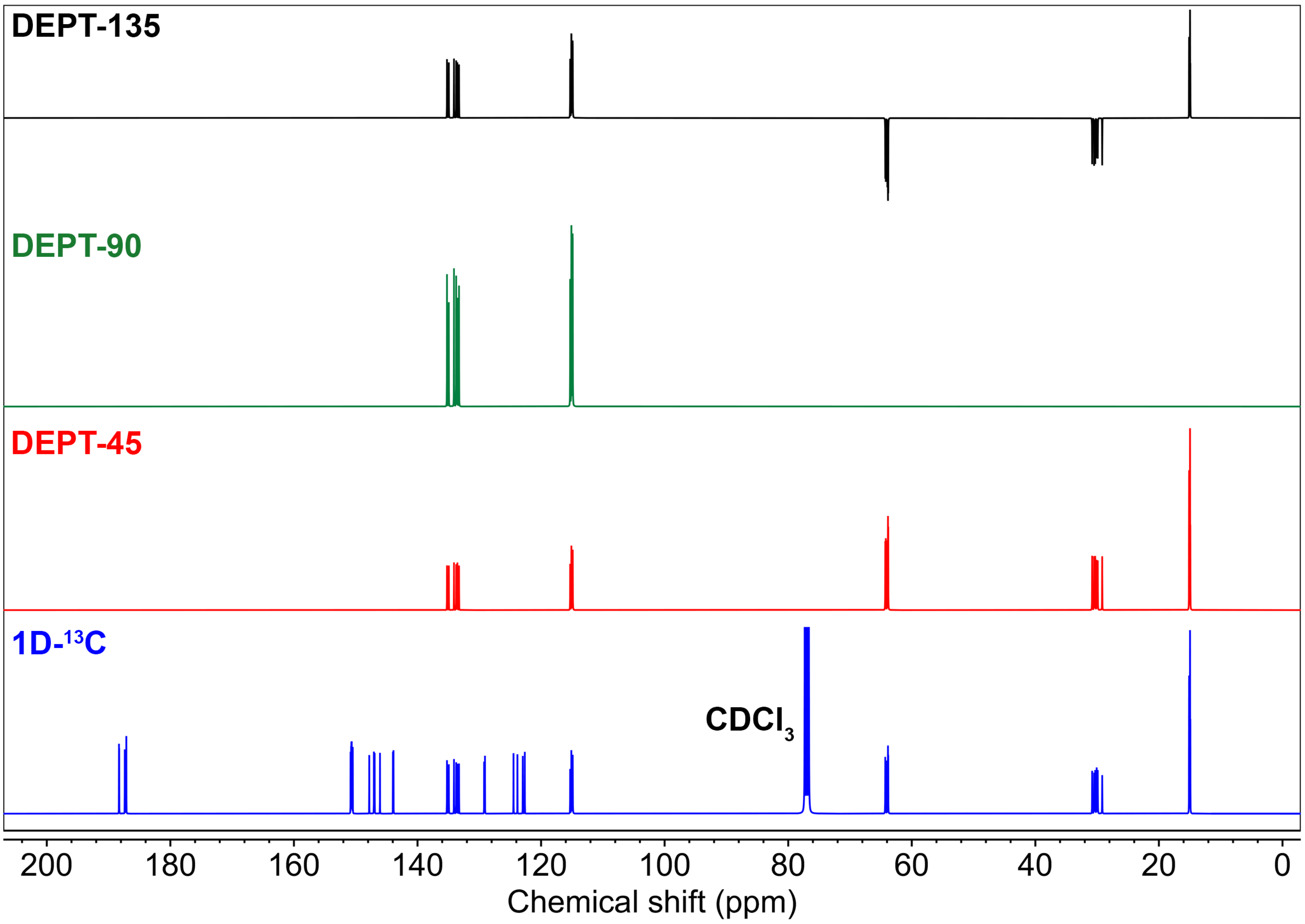

Supplementary Figure 13. 1D-^{13}CNMR and DEPT spectra of P3Q-B at 100 MHz in $CDCl_3$ at 25 ℃

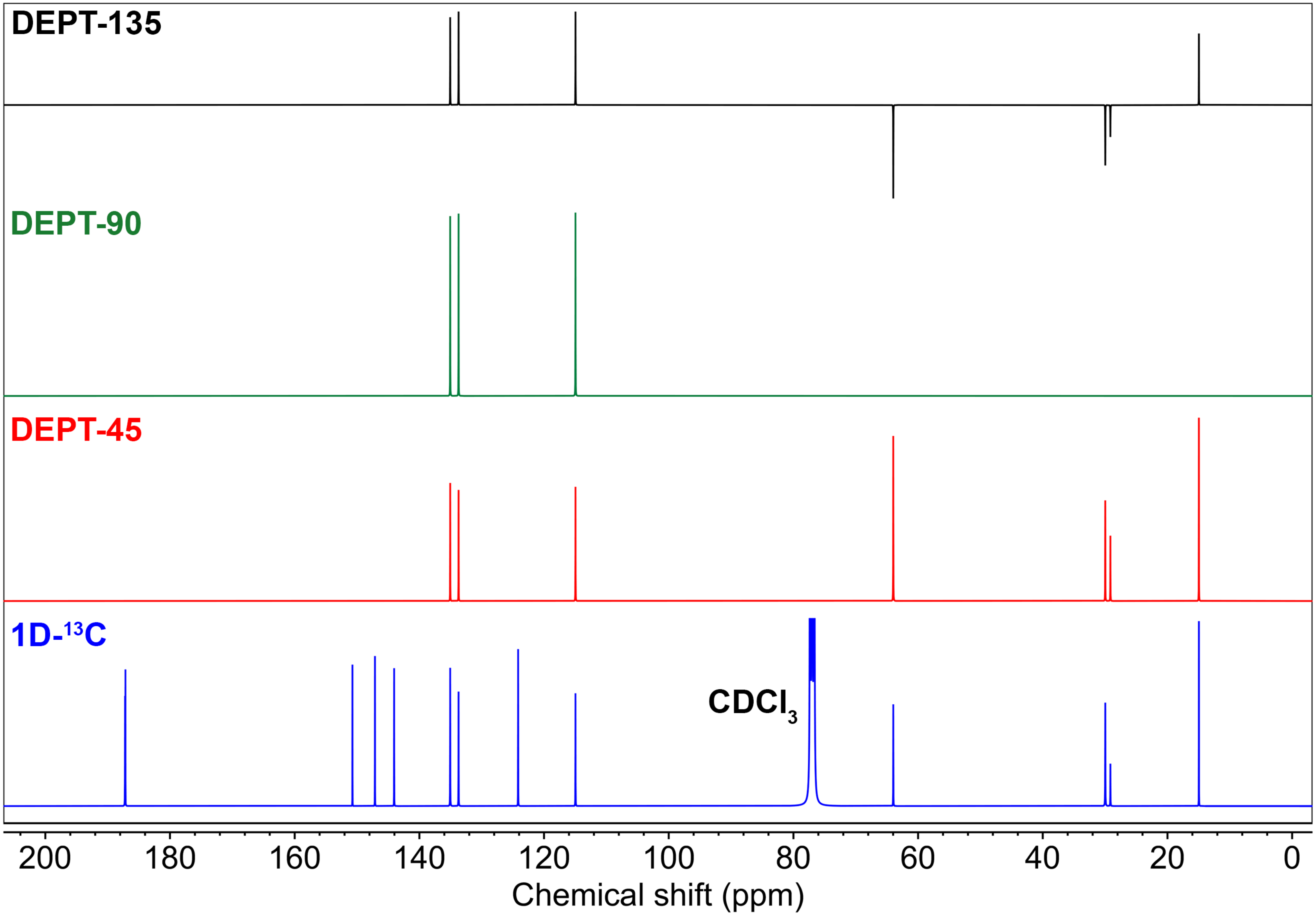

Supplementary Figure 14. 1D-^{13}CNMR and DEPT spectra of P4Q-A at 100 MHz in $CDCl_3$ at 25 ℃

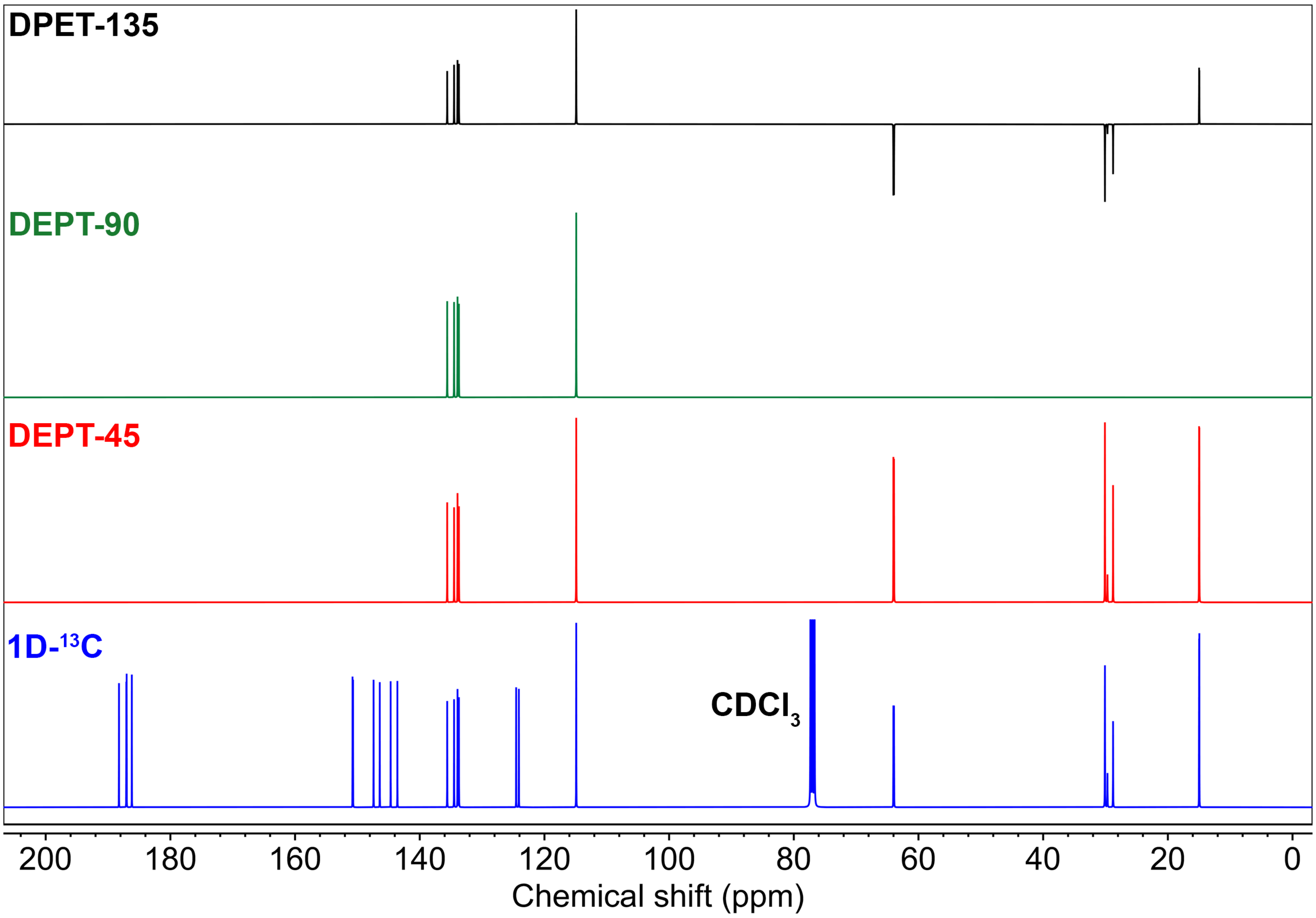

Supplementary Figure 15. 1D-^{13}CNMR and DEPT spectra of P4Q-B at 100 MHz in $CDCl_3$ at 25 ℃

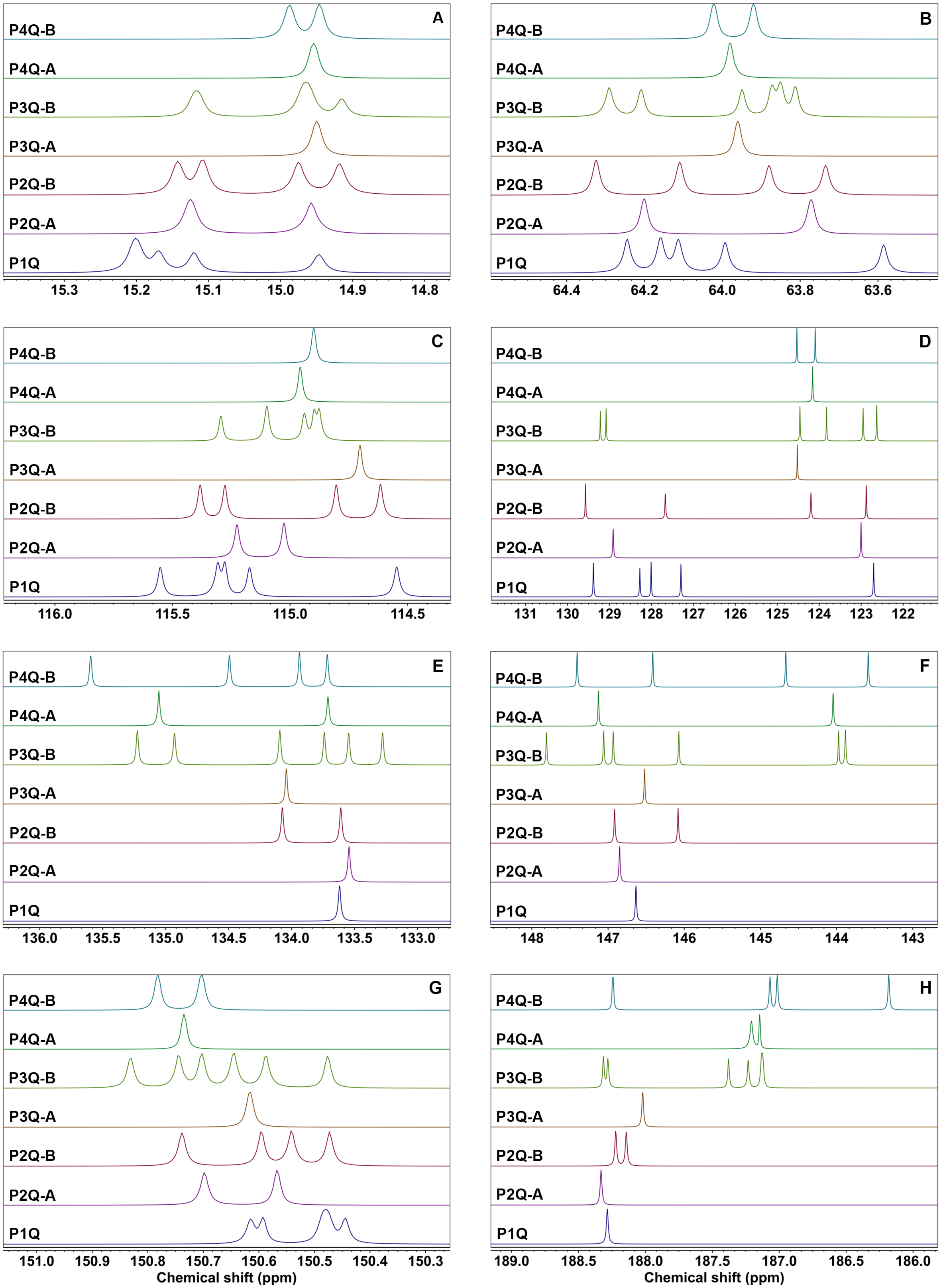

Supplementary Figure 16. Magnified ^{13}C NMR spectra showing carbon signals in various regions (A) -O-CH$_2$-**C**H$_3$, (B) -O-**C**H$_2$-CH$_3$, (C) **C**$_{Ar}$H, (D) **C**$_{Ar}$-CH$_2$-, (E) **C**$_Q$H, (F) **C**$_{Ar}$-CH$_2$-, (G) **C**$_{Ar}$-OEt and (H) **C**$_Q$=O.

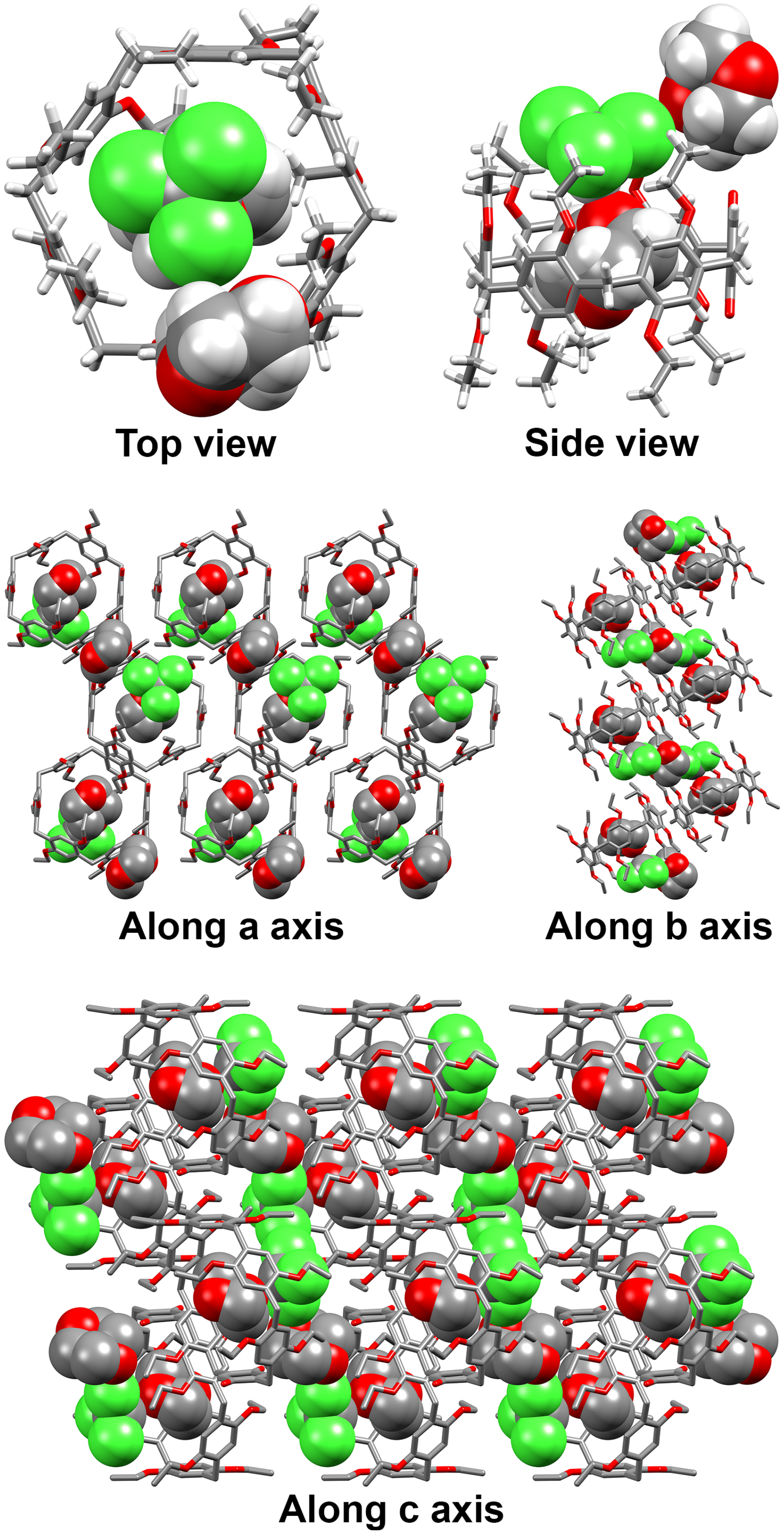

Supplementary Figure 17. Single X-ray structure of P1Q. C gray, O red, Cl green and H white.

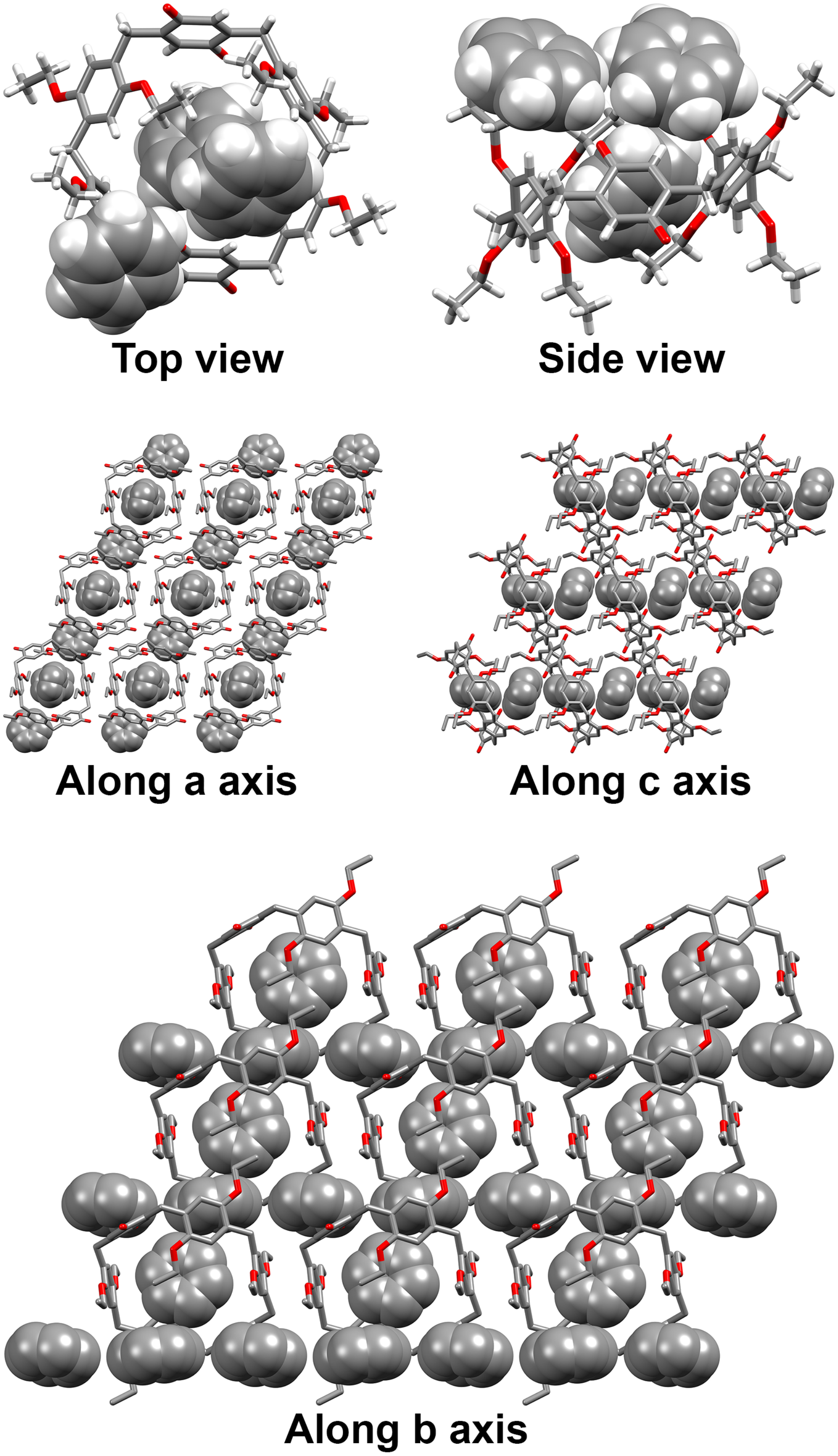

Supplementary Figure 18. Single X-ray structure of P2Q-A. C gray, O red and H white.

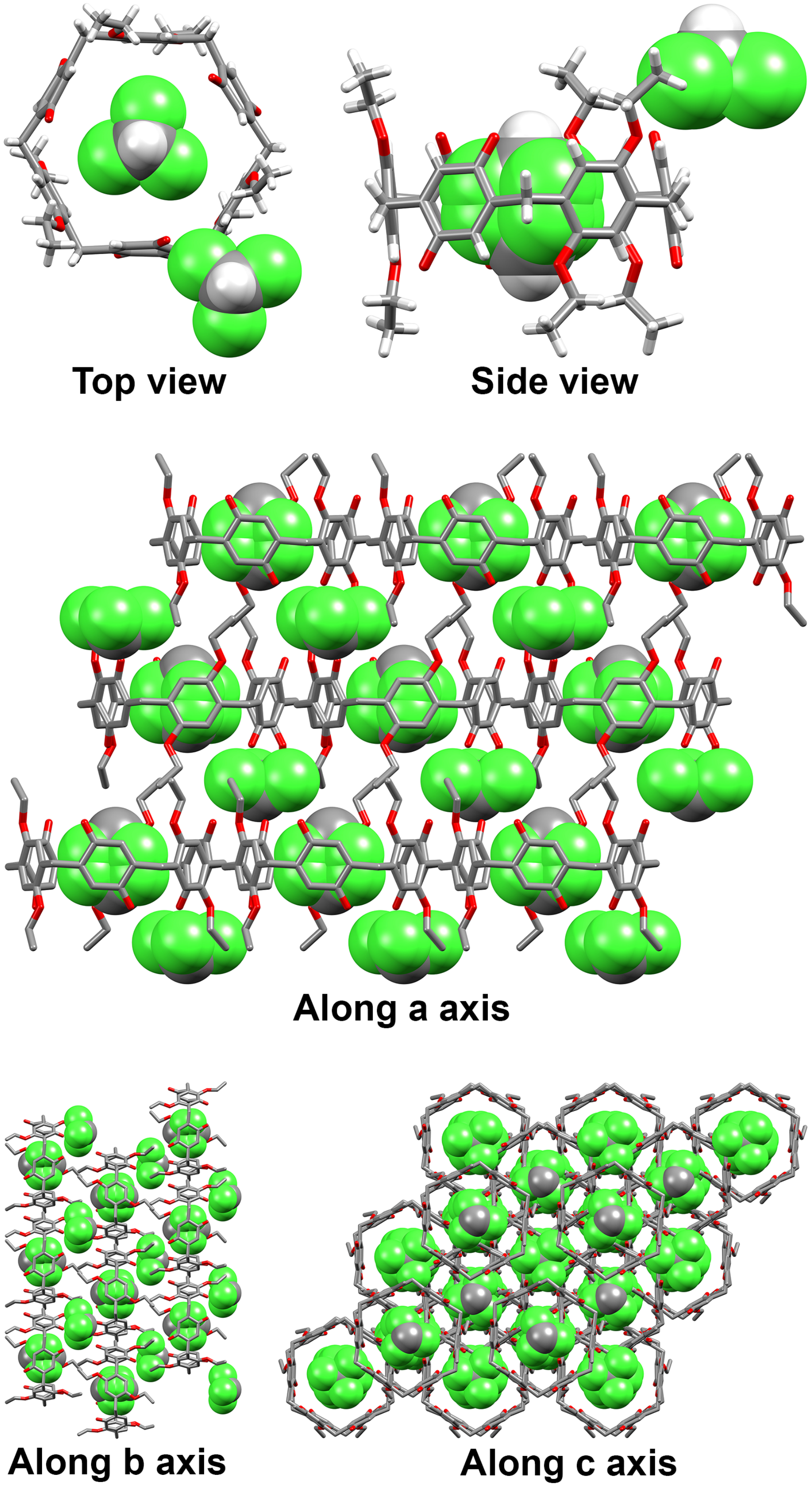

Supplementary Figure 19. Single X-ray structure of P3Q-A. C gray, O red, Cl green and H white.

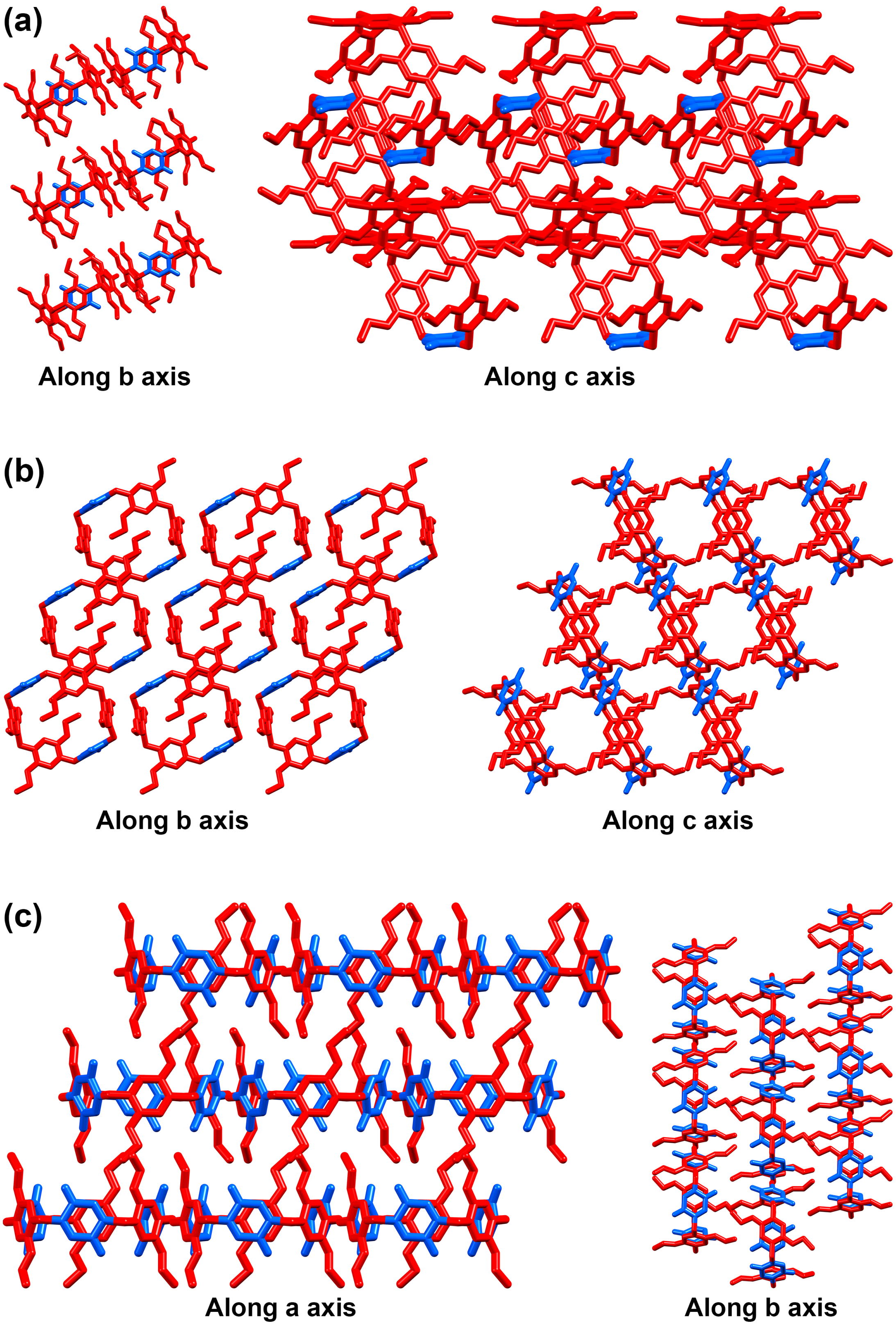

Supplementary Figure 20. Different perspectives of packing of crystal structures of (a) P1Q, (b) P2Q-A and (c) P3Q-A. Hydrogen atoms are eliminated for clarity.

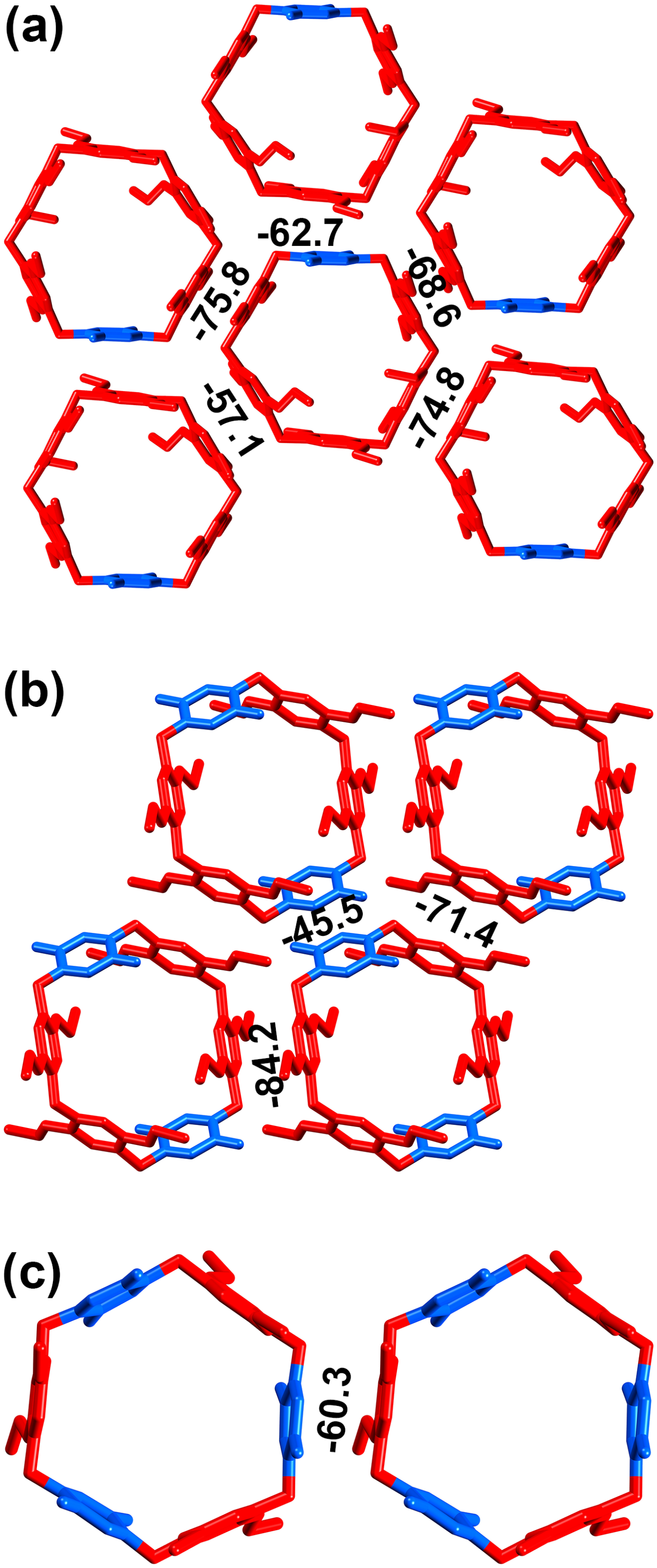

Supplementary Figure 21. Intermolecular potential (KJ/mol) between macrocycles (a) P1Q, (b) P2Q-A and (c) P3Q-A in their solid crystalline state.

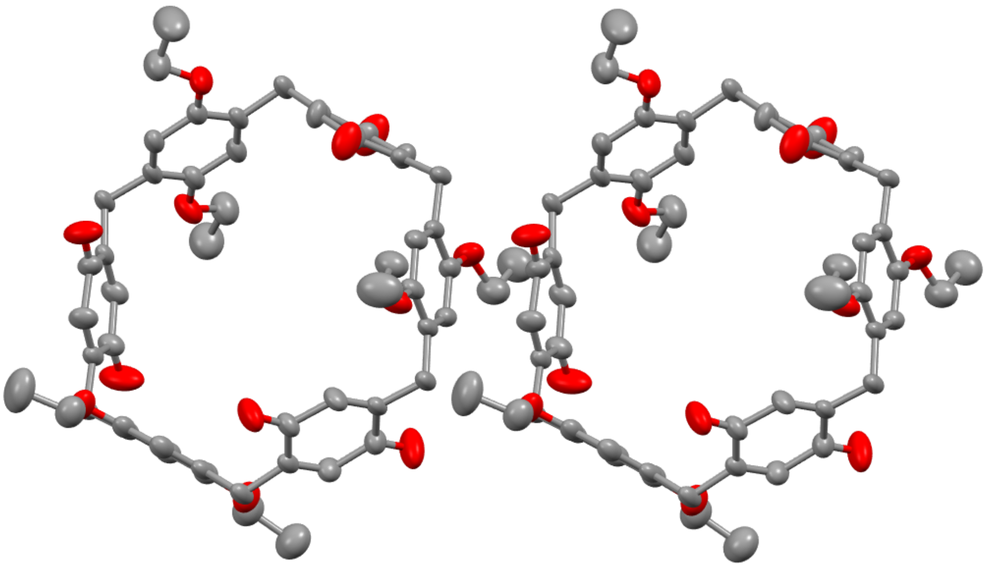

Supplementary Figure 22. Charge transfer interaction between two P3Q-A macrocycles in the solid state. The distance between the centroids of the "contacting" aromatic and quinone rings was measured as 3.78 Å.

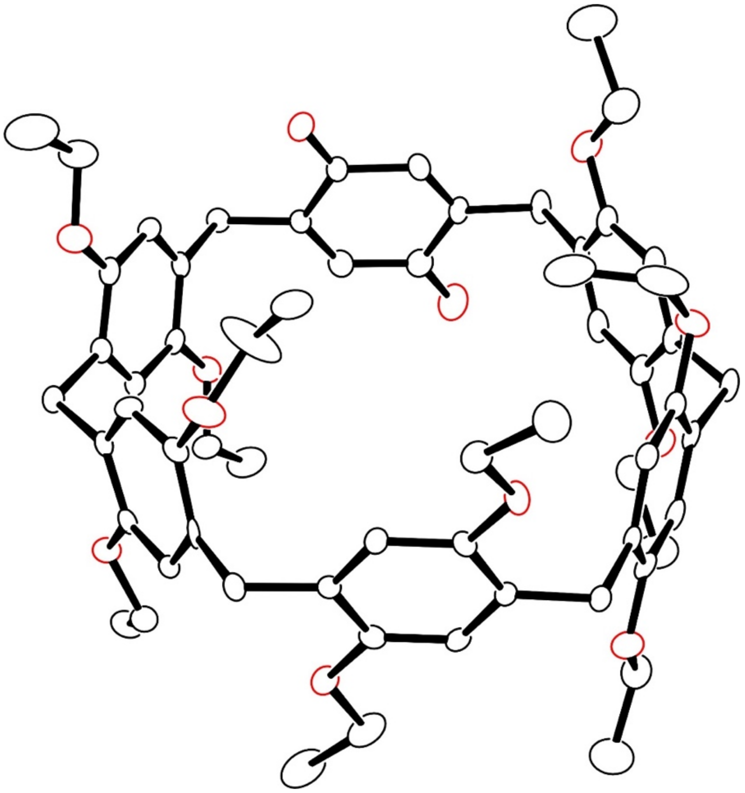

Supplementary Figure 23. An ORTEP showing the molecular structure of P1Q at 50% thermal ellipsoid probability.

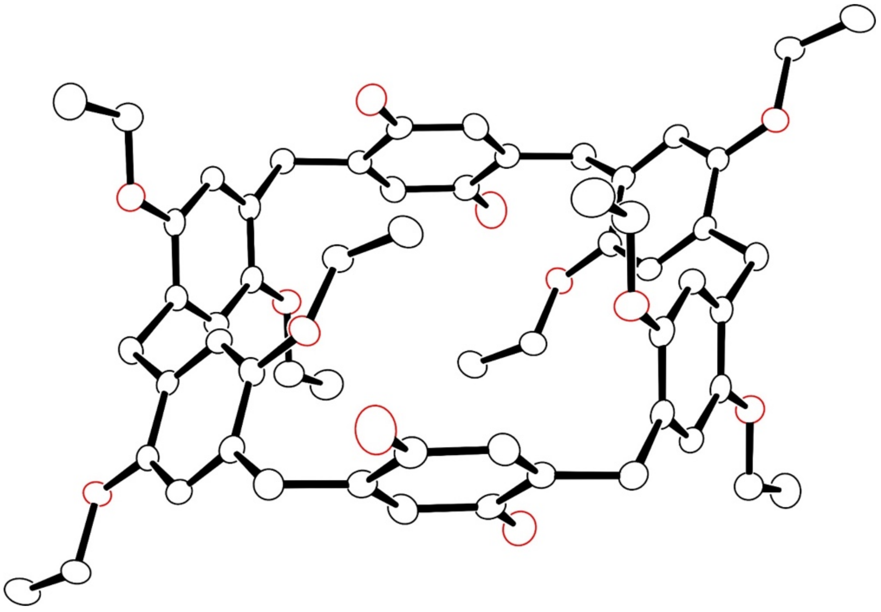

Supplementary Figure 24. An ORTEP showing the molecular structure of P2Q-A at 50% thermal ellipsoid probability.

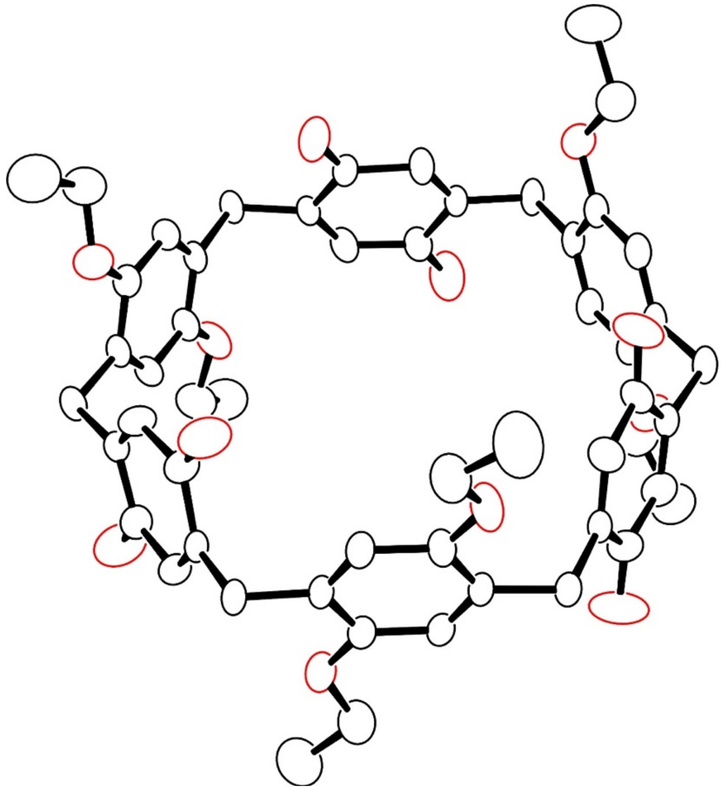

Supplementary Figure 25. An ORTEP showing the molecular structure of P3Q-A at 40% thermal ellipsoid probability.

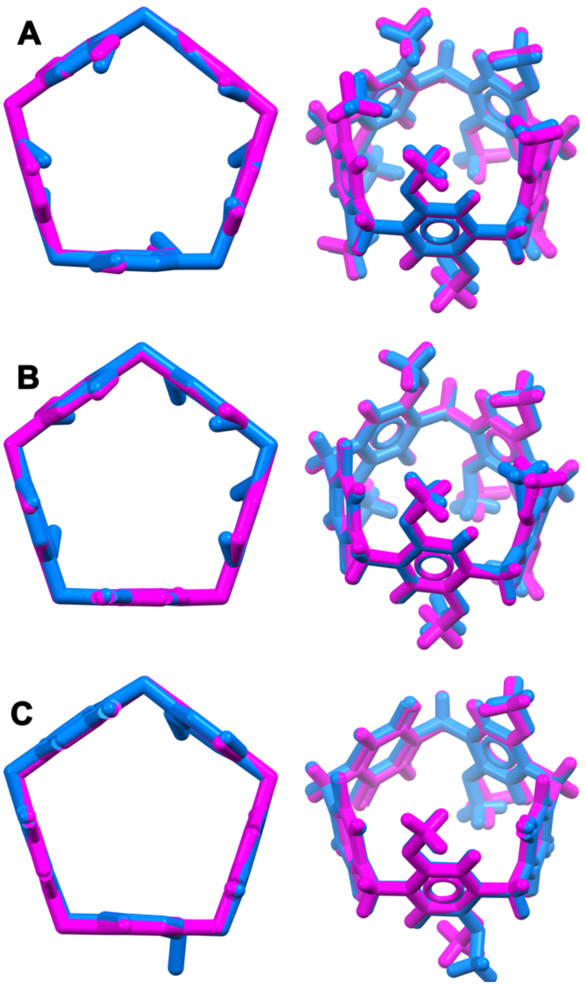

Supplementary Figure 26. Superimposed optimized structures (pink) [M062X/6-31G+(d,p)] of P’5A, P’1Q and P’3Q over their corresponding X-ray structures (blue). Left and right panels show different perspectives for clarity.

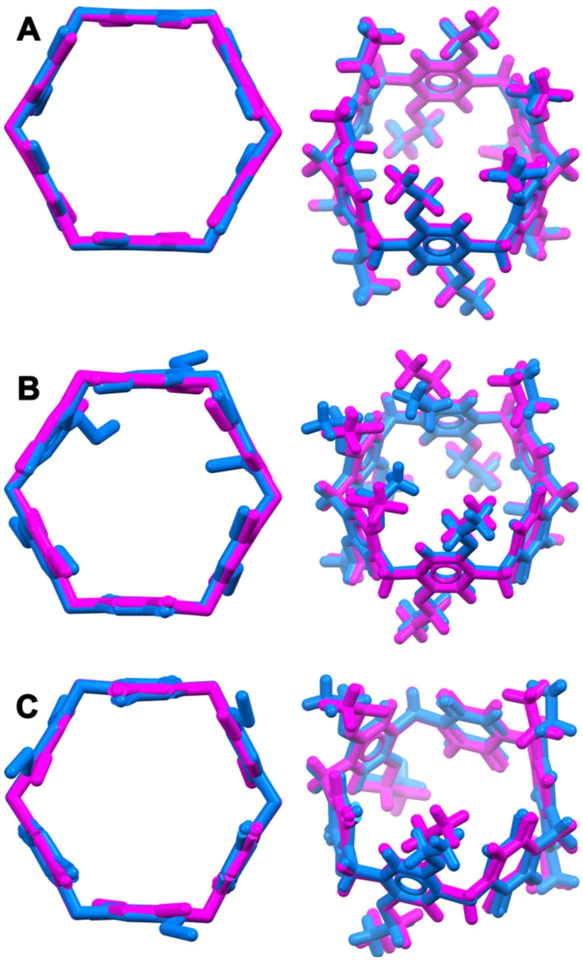

Supplementary Figure 27. Superimposed optimized structures (pink) [M062X/6-31G+(d,p)] of P6A, P1Q and P3Q-A over their corresponding X-ray structures (blue). Left and right panels show different perspectives for clarity.

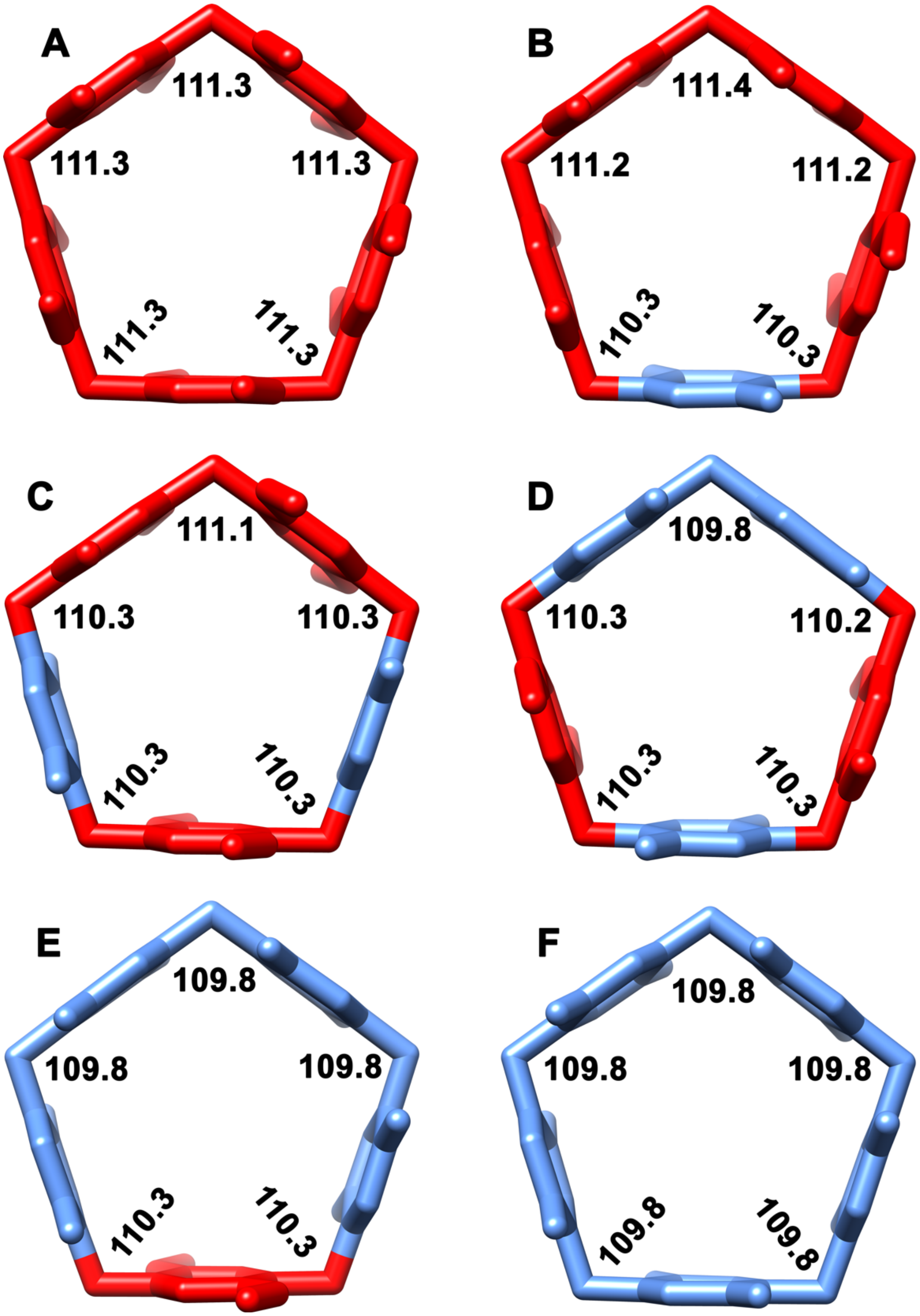

Supplementary Figure 28. Interior angles of (A) P'5A, (B) P'1Q, (C) P'2Q, (D) P'3Q, (E) P'4Q, (F) P'5Q compounds optimized at the M062X/6-31G+(d,P) level in the presence of solvent (CH_2Cl_2).

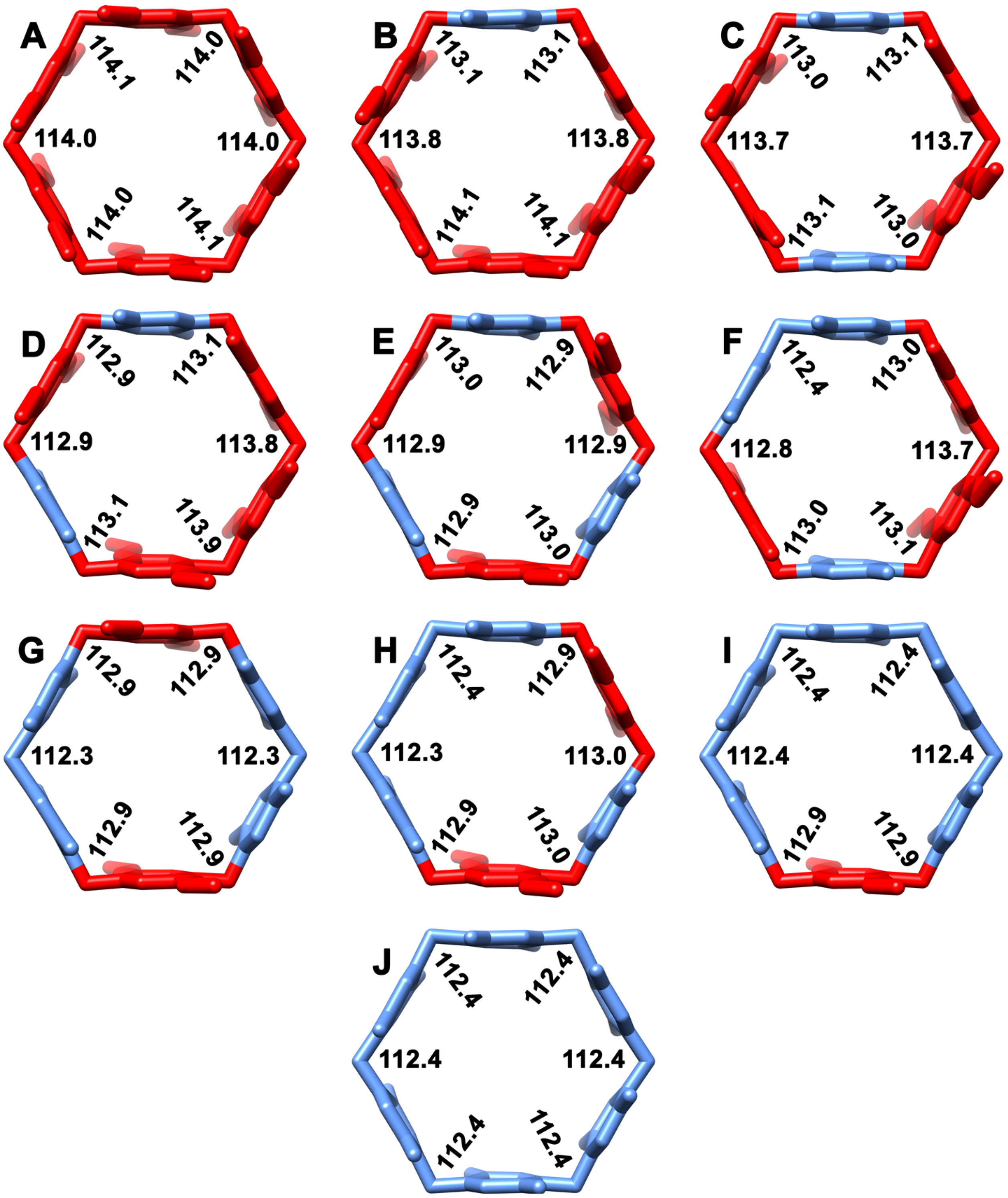

Supplementary Figure 29. Interior angles of (A) P6A, (B) P1Q, (C) P2Q-A, (D) P2Q-B, (E) P3Q-A, (F) P3Q-B, (G) P4Q-A, (H) P4Q-B, (I) P5Q, (J) P6Q compounds optimized at M062X/6-31G+(d,P) level in the presence of solvent (CH_2Cl_2).

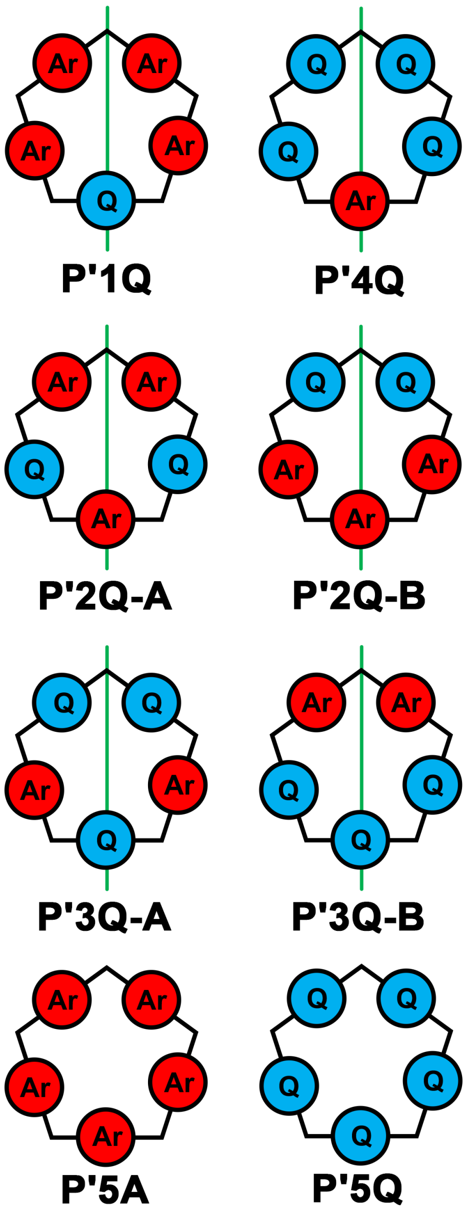

Supplementary Figure 30. Schematic representation of oxidized derivatives of per-methylatedpillar[5]arene (P'5A). The red circles labeled 'Ar' symbolize 1,4-dimethoxybenzene units and the blue circles labeled 'Q' represent 1,4-benzoquinone units. The green lines represent C2 axes that are useful to determine the number of ^{13}C or ^{1}H NMR resonances for each macrocycle.

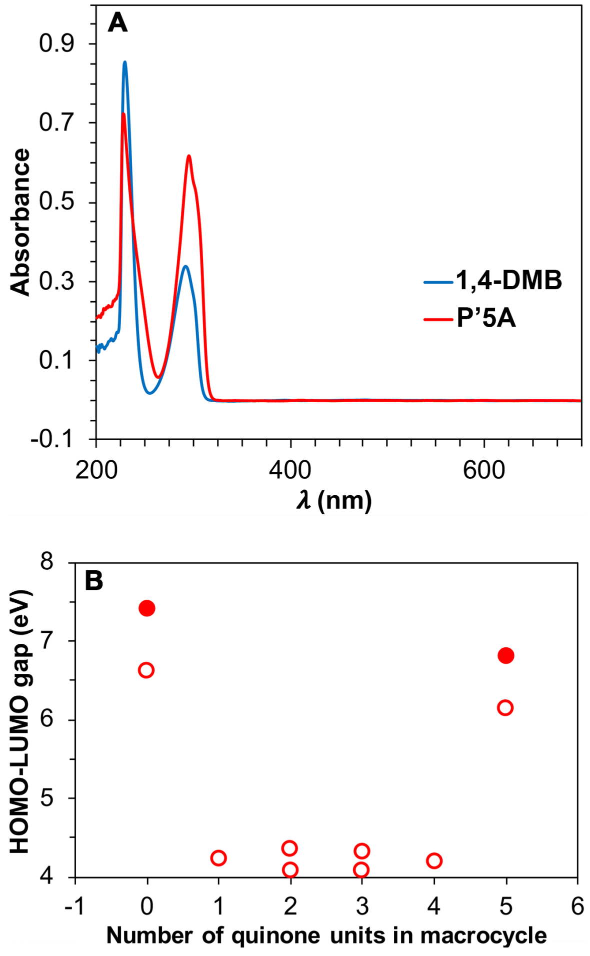

Supplementary Figure 31. (A) UV-vis spectra of P'5A and 1,4-dimethoxybenzene (1,4-DMB) in CH_2Cl_2 solution (the concentration of macrocycle solutions is about 25 μM and the concentration of 1,4-DMB monomer solution is about 0.1 mM). (C) Plot of the value of HOMO-LUMO gap of P'5A, 1,4-DMB and also the oxidized derivatives of P'5A versus the number of quinone units present in the compounds (Calculated at M062X/6-31G+(d,p) level including CH_2Cl_2 as an implicit solvent).

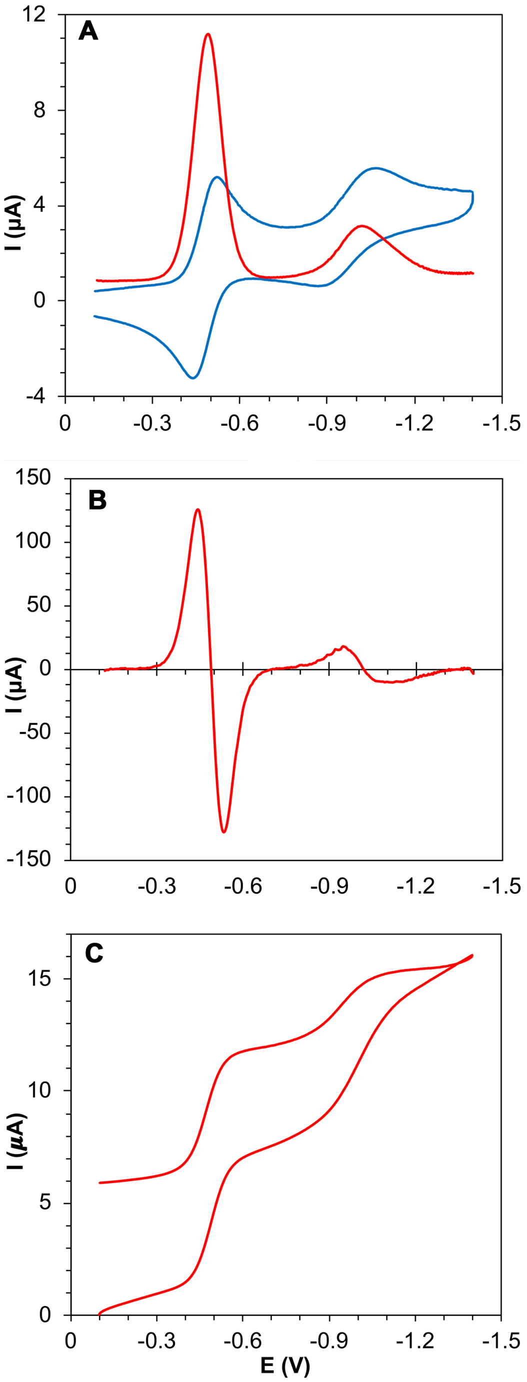

Supplementary Figure 32. (A) CV (blue) and SWV (red) of 0.25 mM **P1Q** using GC working electrode (0.07 cm^2) in 0.1 M DCM solution of TBAF at 100 mV/s and 60 mV/s, respectively. (B) First derivative of the SWV and (C) Semi-integral of CV in panel A.

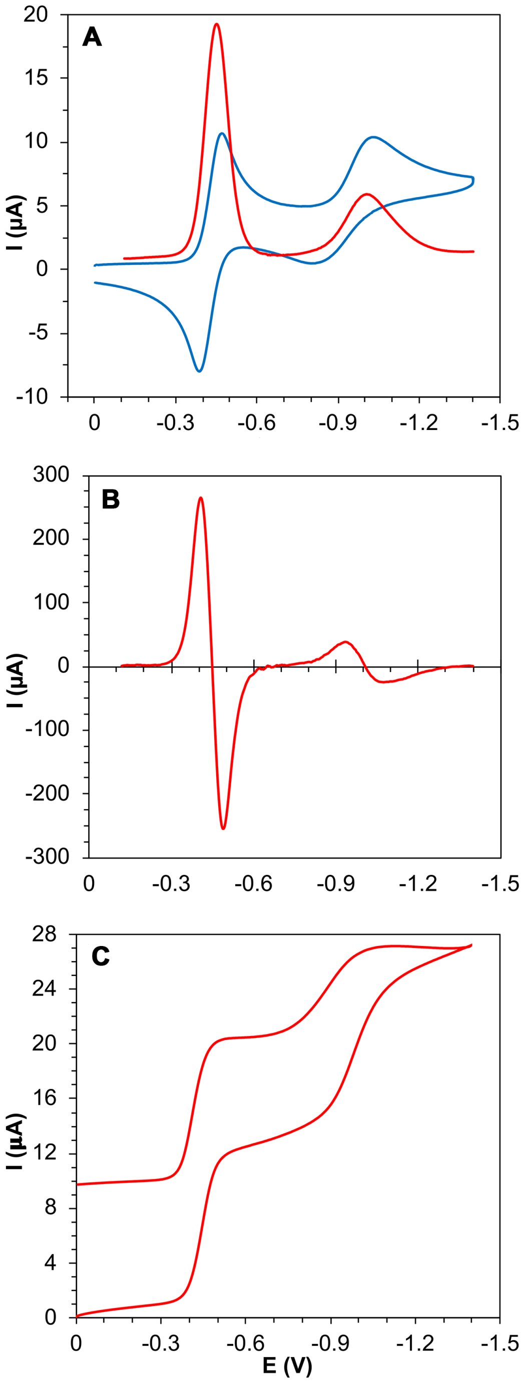

Supplementary Figure 33. (A) CV (blue) and SWV (red) of 0.25 mM **P2Q-A** using GC working electrode (0.07 cm^2) in 0.1 M DCM solution of TBAF at 100 mV/s and 60 mV/s, respectively. (B) First derivative of the SWV and (C) Semi-integral of CV in panel A.

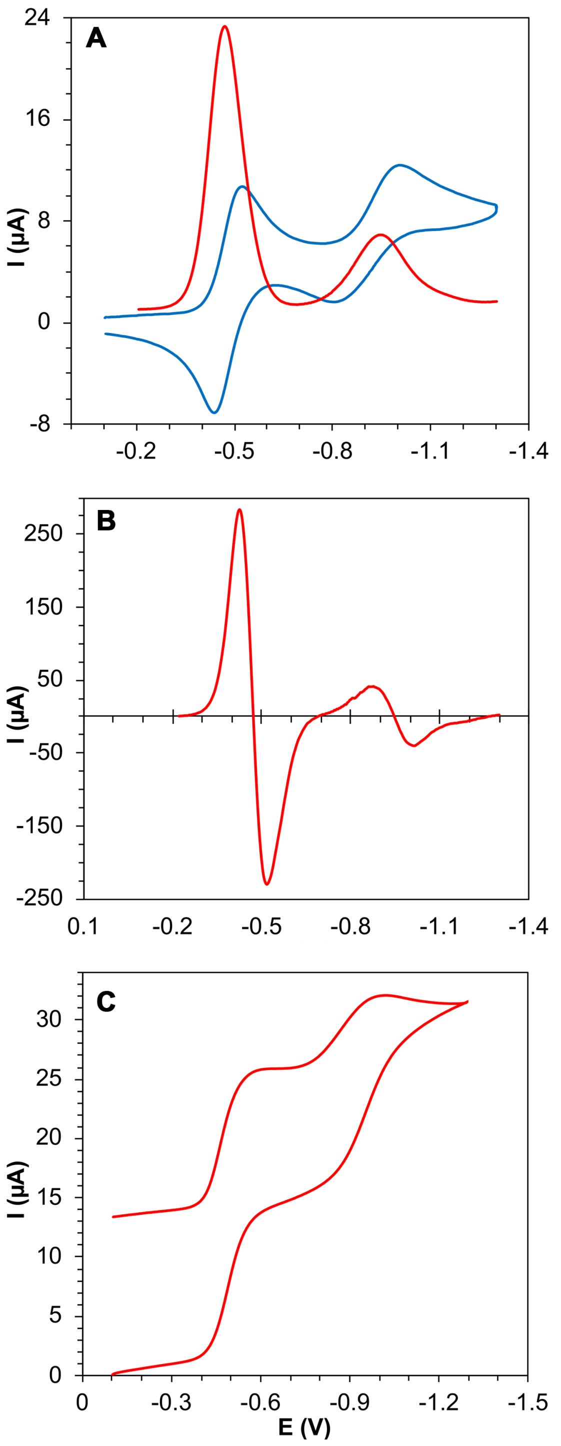

Supplementary Figure 34. (A) CV (blue) and SWV (red) of 0.25 mM **P2Q-B** using GC working electrode (0.07 cm^2) in 0.1 M DCM solution of TBAF at 100 mV/s and 60 mV/s, respectively. (B) First derivative of the SWV and (C) Semi-integral of CV in panel A.

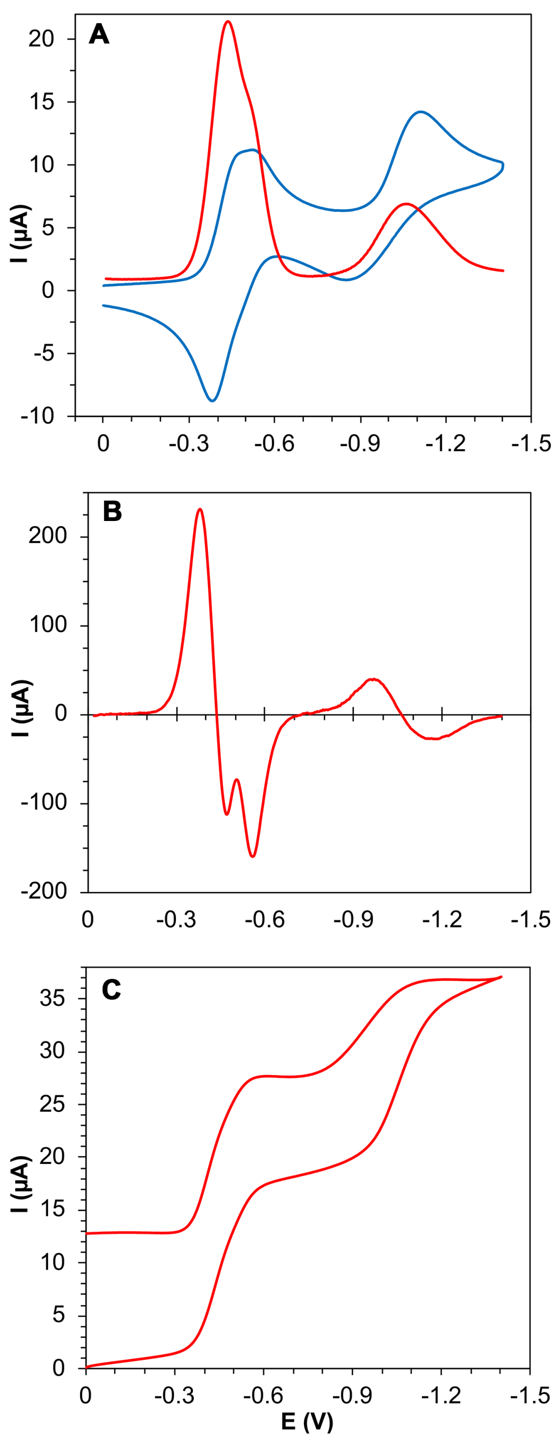

Supplementary Figure 35. (A) CV (blue) and SWV (red) of 0.25 mM **P3Q-A** using GC working electrode (0.07 cm^2) in 0.1 M DCM solution of TBAF at 100 mV/s and 60 mV/s, respectively. (B) First derivative of the SWV and (C) Semi-integral of CV in panel A.

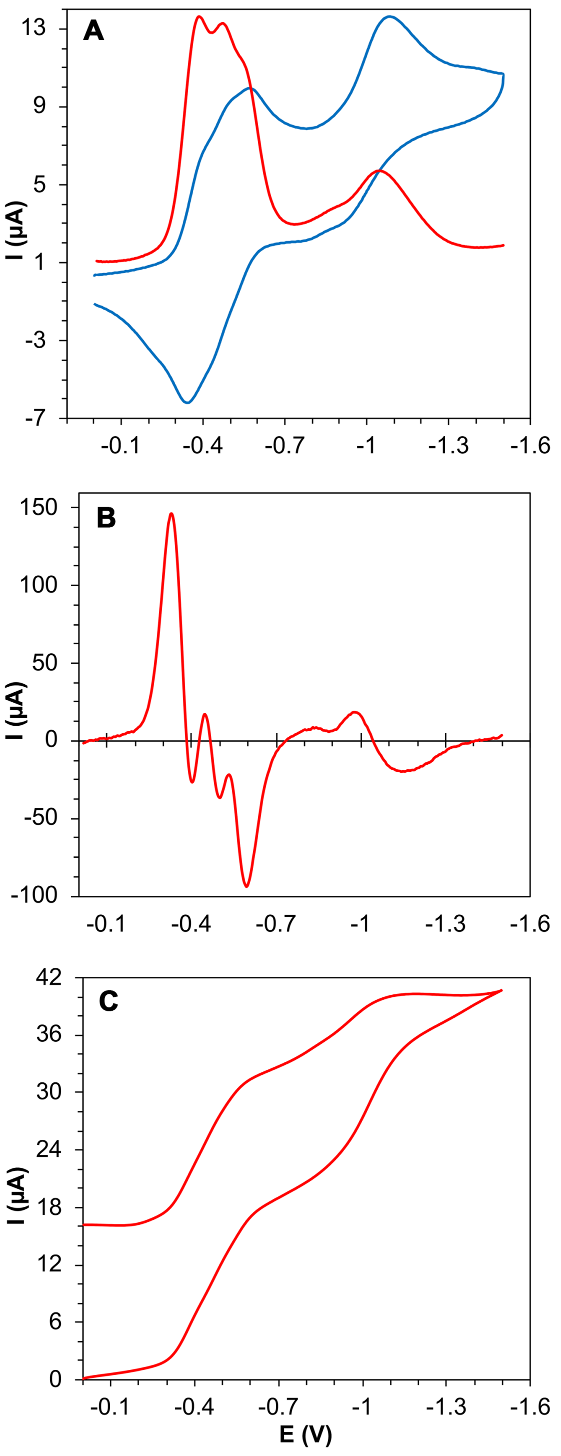

Supplementary Figure 36. (A) CV (blue) and SWV (red) of 0.25 mM **P3Q-B** using GC working electrode (0.07 cm^2) in 0.1 M DCM solution of TBAF at 100 mV/s and 60 mV/s, respectively. (B) First derivative of the SWV and (C) Semi-integral of CV in panel A.

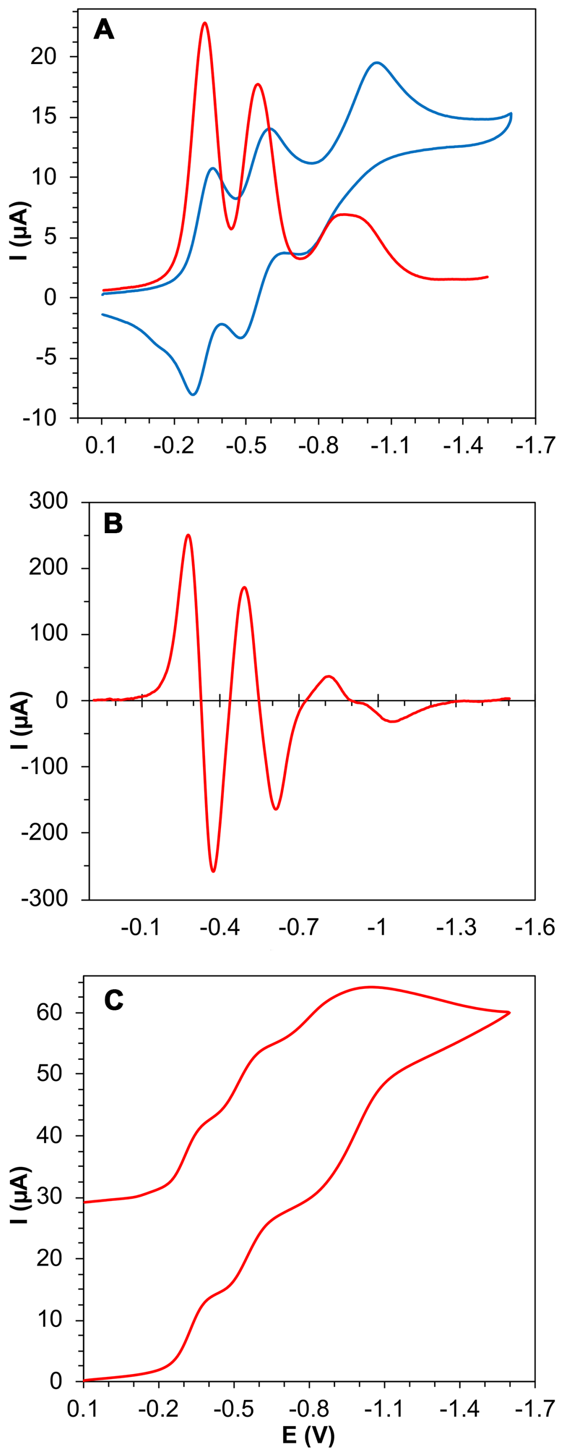

Supplementary Figure 37. (A) CV (blue) and SWV (red) of 0.25 mM **P4Q-A** using GC working electrode (0.07 cm^2) in 0.1 M DCM solution of TBAF at 100 mV/s and 60 mV/s, respectively. (B) First derivative of the SWV and (C) Semi-integral of CV in panel A.

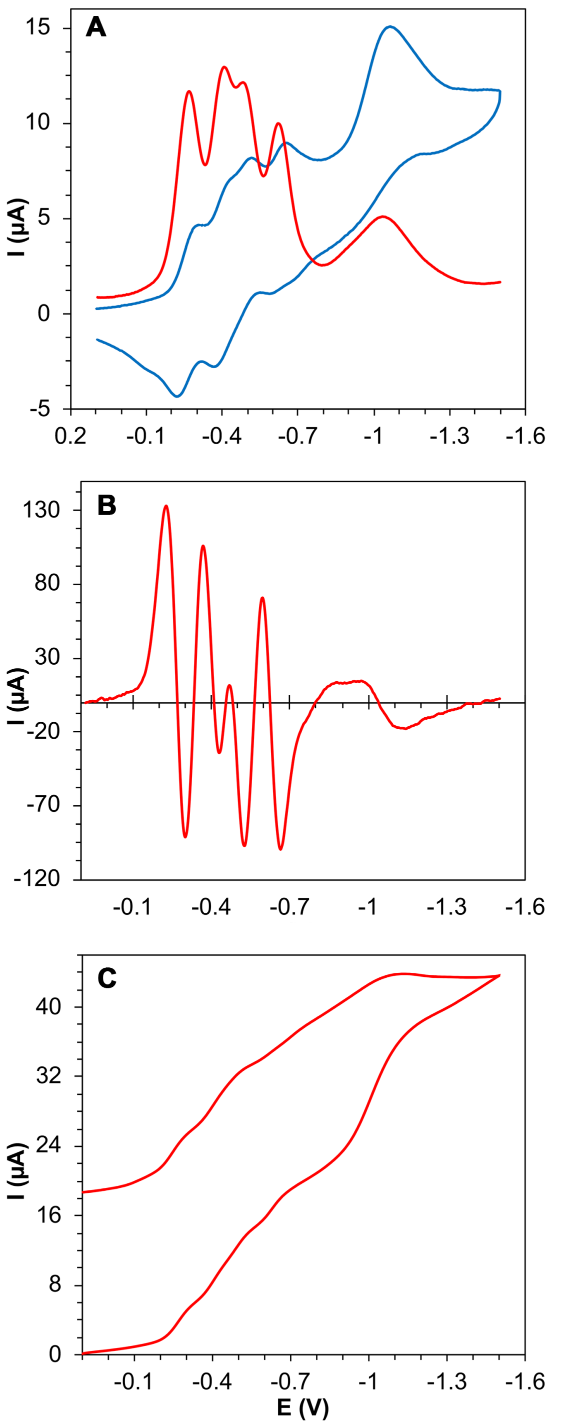

Supplementary Figure 38. (A) CV (blue) and SWV (red) of 0.25 mM **P4Q-B** using GC working electrode (0.07 cm^2) in 0.1 M DCM solution of TBAF at 100 mV/s and 60 mV/s, respectively. (B) First derivative of the SWV and (C) Semi-integral of CV in panel A.

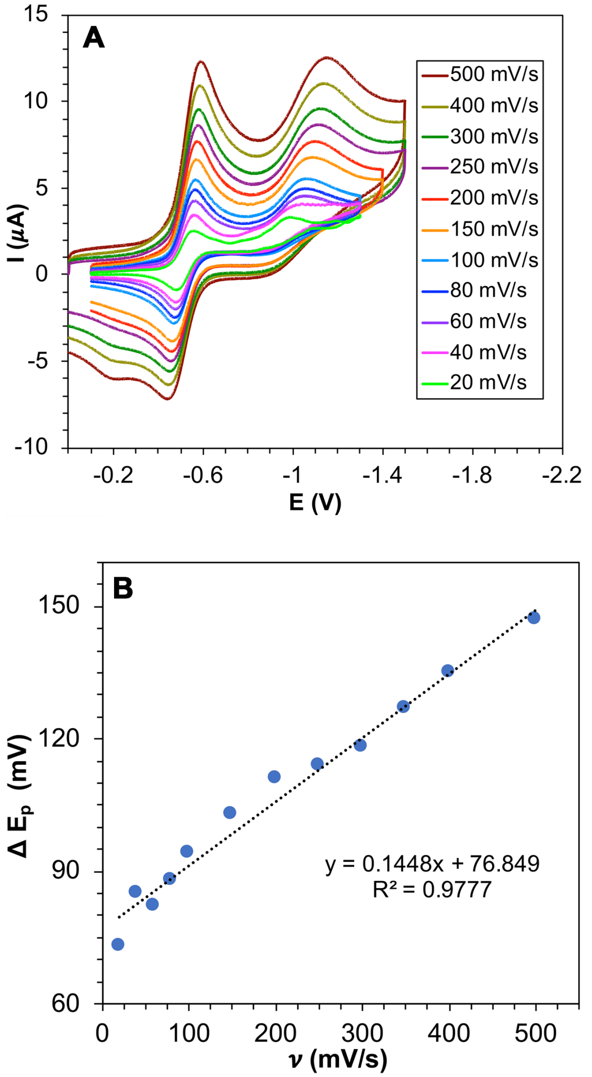

Supplementary Figure 39. (A) Cyclic volatmmograms of 0.25 mM P1Q using GC working electrode (0.07 cm^2) in 0.1 M CH_2Cl_2 solution of TBAP at different scan rates. (B) Plot of the first redox peak potential of CVs in panel A versus their corresponding scan rates

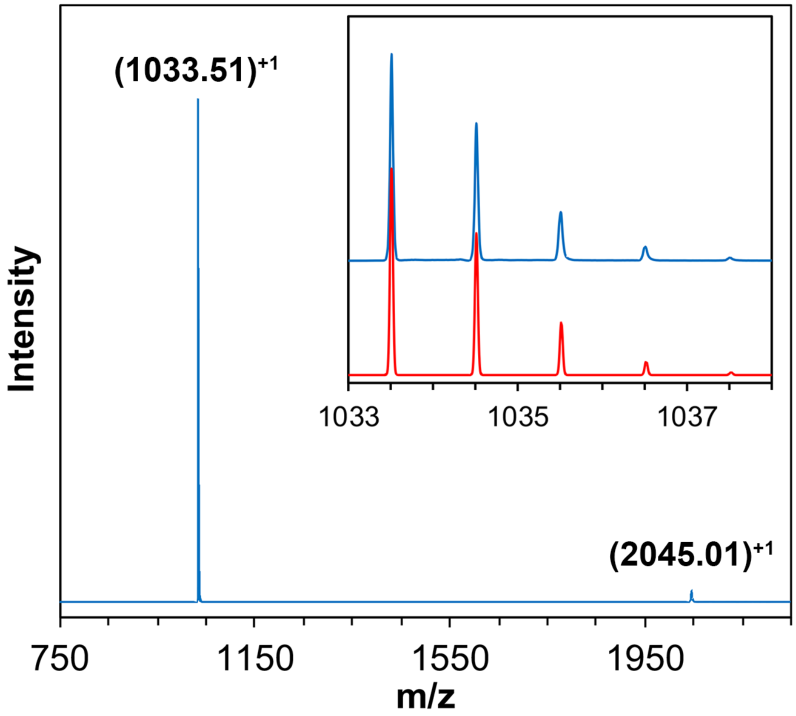

Supplementary Figure 40. ESI mass spectrum of P1Q. The main peak at m/z 1033.51 corresponds to $M+Na^{+}$.

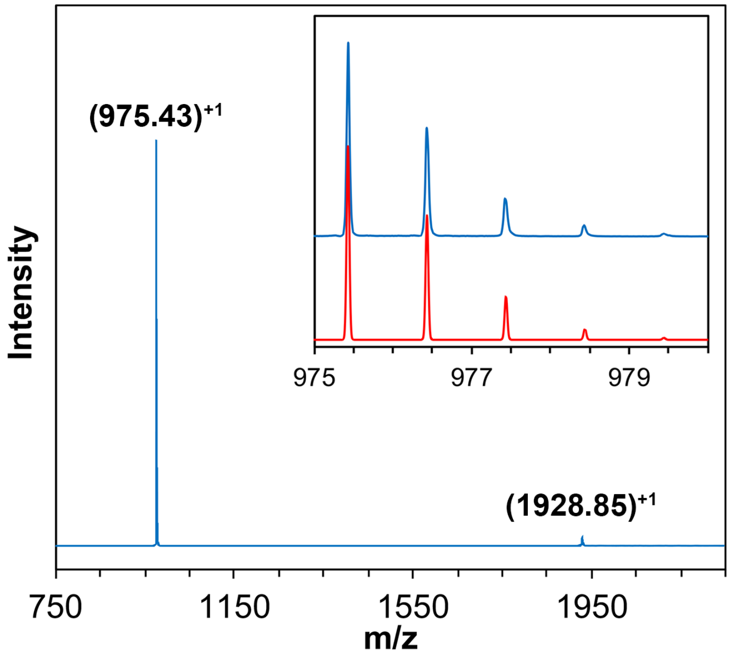

Supplementary Figure 41. ESI mass spectrum of P2Q-A. The main peak at m/z 975.43 corresponds to $M+Na^{+}$.

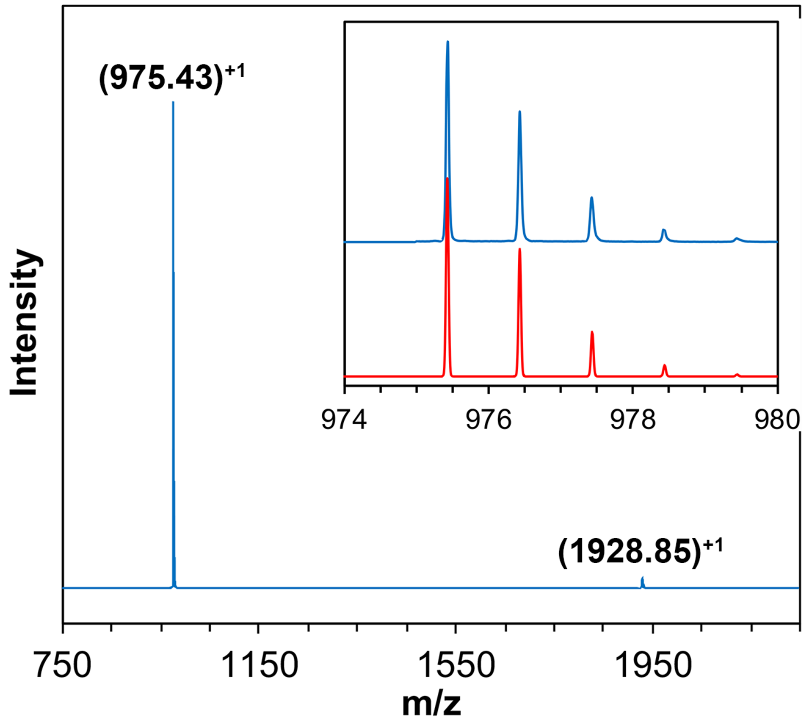

Supplementary Figure 42. ESI mass spectrum of P2Q-B. The main peak at m/z 975.43 corresponds to M+Na^{+}.

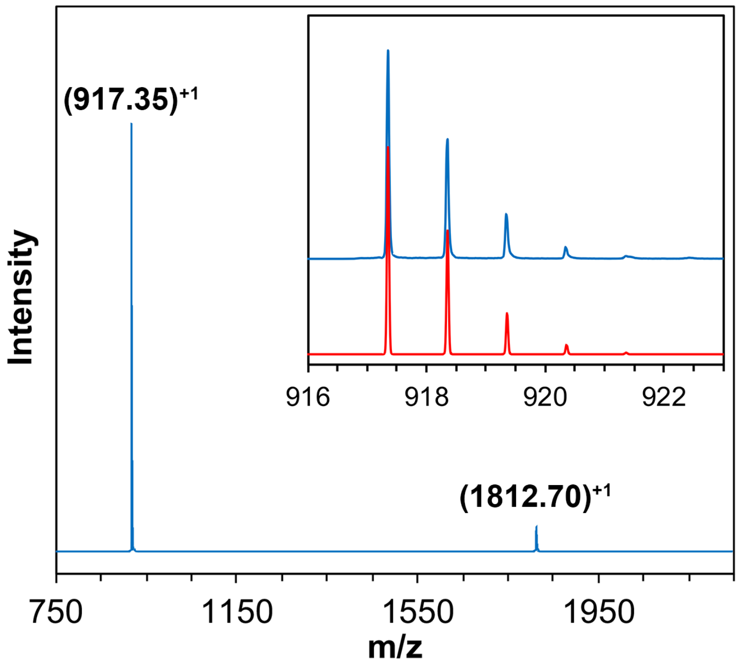

Supplementary Figure 43. ESI mass spectrum of P3Q-A. The main peak at m/z 917.35 corresponds to M+Na^{+}.

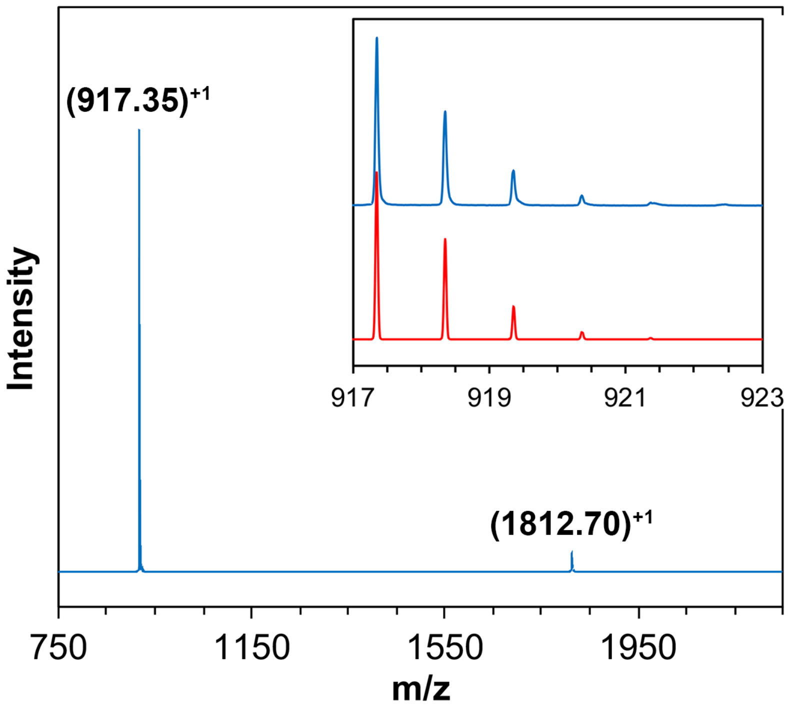

Supplementary Figure 44. ESI mass spectrum of P3Q-B. The main peak at m/z 917.35 corresponds to $M+Na^+$.

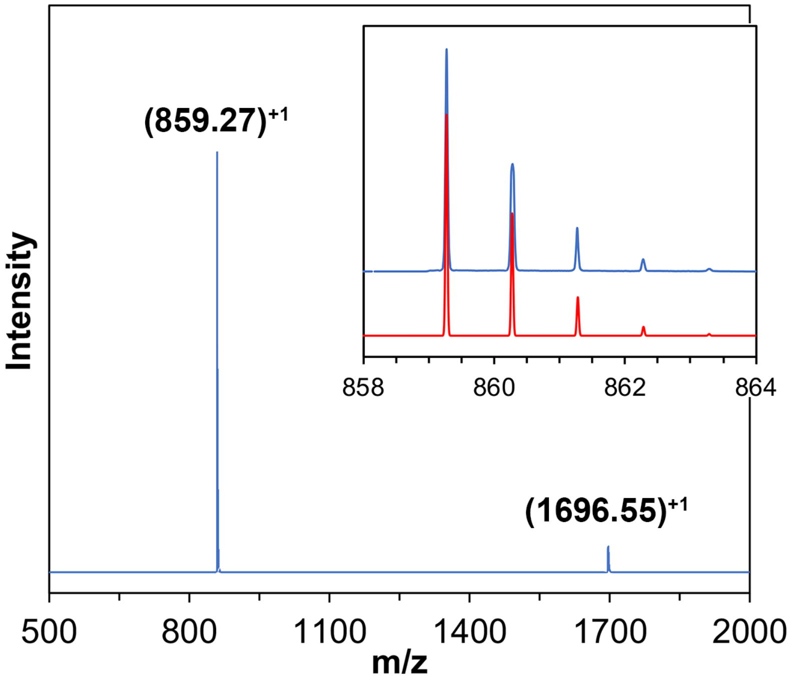

Supplementary Figure 45. ESI mass spectrum of P4Q-A. The main peak at m/z 859.27 corresponds to $M+Na^+$.

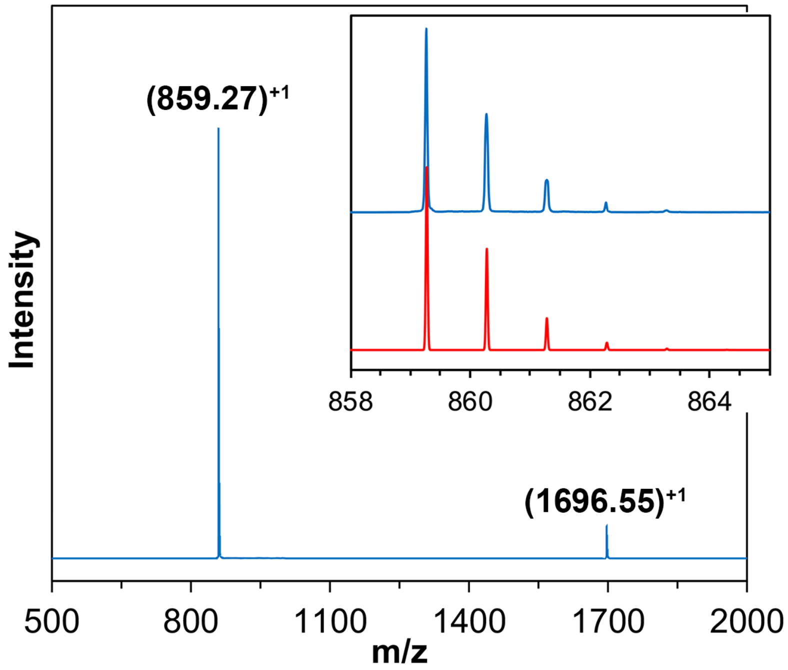

Supplementary Figure 46. ESI mass spectrum of P4Q-B. The main peak at m/z 859.27 corresponds to M+Na$^+$.

Supplementary Table 1. The root mean square deviation (RMSD) and the maximum deviation (D_{max}) in Å obtained from overlaying of the optimized structures of pillararene compounds in Figures 3 & 4 onto their X-ray structures.

Compound	RMSD (Å)	D_{max} (Å)	Ref.
Supplementary Figure 26			
P5A	0.3311	1.8608	1
P1Q	0.1724	0.5222	2
P3Q	0.1848	0.6028	3
Supplementary Figure 27			
P6A	0.1528	0.4196	4
P1Q	0.5328	2.6140	This work
P3Q-A	0.3935	1.2140	This work

Supplementary Table 2. CIV values (in electron/bohr) for oxidized derivatives of P'5A and P6A compounds.

Compound	Critical Iso-surface Value (CIV)	
	Through-bond	Through-space
1,4-DimethoxybenzenePillar(5)arene		
P'5A	0.033	0.025
P'1Q	0.033	0.025
P'2Q	0.033	0.025
P'3Q	0.033	0.026
P'4Q	0.032	0.026
P'5Q	0.033	0.025
1,4-DiethoxybenzenePillar(6)arene		
P6A	0.033	0.019 — 0.028
P1Q	0.034	0.021 — 0.027
P2Q-A	0.033	0.022 — 0.029
P2Q-B	0.033	0.020 — 0.029
P3Q-A	0.033	0.023 — 0.029
P3Q-B	0.031	0.020 — 0.029
P4Q-A	0.034	0.022 — 0.028
P4Q-B	0.034	0.024 — 0.026

Supplementary Table 3. UV-vis absorption data of 1,4-diethoxybenzene, 1,4-dimethoxybenzene, and oxidized derivatives of pillar[6]arene, and pillar[5]arene.

Compound	λ_{max} (nm)
1,4-Diethoxybenzene (1,4-DEB)	292
P6A	294
P1Q	294
P2Q-A	294
P2Q-B	292
P3Q-A	292
P3Q-B	292
P4Q-A	292
P4Q-B	292
1,4-dimethoxybenzene (1,4-DMB)	292
P'5A	295

Supplementary Table 4. The number of aromatic-quinone proximities and the structural parameters of the studied macrocycles.

Compound	Sum of interior angles (in degree)	Angle strain (in degree)	The number of aromatic-quinone proximities
P'5A	556.5	-16.5	0
P'1Q	554.4	-14.4	2
P'2Q	552.3	-12.3	4
P'3Q	550.9	-10.9	4
P'4Q	550	-10	2
P'5Q	549	-9	0
P6A	684.2	35.8	0
P1Q	682	38	2
P2Q-A	679.6	40.4	4
P2Q-B	679.7	40.3	4
P3Q-A	677.6	42.4	6
P3Q-B	678	42	4
P4Q-A	676.2	43.8	4
P4Q-B	676.5	43.5	4
P5Q	675.4	44.6	2
P6Q	674.4	45.6	0

Supplementary Table 5. The energies (eV) of HOMO, LUMO and LUMO-HOMO gap for different derivatives of per-methylated-pillar[5]arene and per-ethylated-pillar(6)arene.

Compound	HOMO (eV)	LUMO (eV)	LUMO-HOMO gap (eV)
1,4-DimethoxyPillar(5)arene			
1,4-dimethoxybenzene	-7.10	0.31	7.41
P’5A	-6.64	-0.03	6.62
P’1Q	-6.73	-2.49	4.24
P’2Q-A	-6.89	-2.54	4.35
P’2Q-B	-6.80	-2.71	4.09
P’3Q-A	-7.06	-2.74	4.32
P’3Q-B	-6.91	-2.83	4.08
P’4Q	-7.09	-2.90	4.20
P’5Q	-9.08	-2.94	6.14
p-Benzoquinone	-9.52	-2.72	6.80
1,4DiethoxyPillar(6)arene			
1,4-diethoxybenzene	-7.96	0.29	8.24
P6A	-6.65	0.02	6.66
P1Q	-6.70	-2.48	4.22
P2Q-A	-6.85	-2.50	4.35
P2Q-B	-6.78	-2.51	4.26
P2Q-C	-6.73	-2.68	4.05
P3Q-A	-7.01	-2.54	4.48
P3Q-B	-6.88	-2.70	4.17
P3Q-C	-6.79	-2.79	4.00
P4Q-A	-7.05	-2.72	4.33
P4Q-B	-7.03	-2.81	4.22
P4Q-C	-6.89	-2.86	4.03
P5Q	-7.06	-2.90	4.17
P6Q	-9.05	-2.94	6.11
P-Benzoquinone	-9.52	-2.72	6.80

Supplementary Table 6. Crystallographic Data for Compounds P1Q, P2Q-A and P3Q-A.

	P1Q	P2Q-A	P3Q-A
Empirical formula	$C_{62}H_{74}O_{12}$ •$CHCl_3$ •1.5 $C_4H_8O_2$	$C_{62}H_{74}O_{12}$ •3 C_6H_6	$C_{54}H_{54}O_{12}$ • 1.5 $CHCl_3$
Formula weight	1262.73	1187.41	1074.02
Crystal system	Triclinic	Triclinic	Trigonal
Lattice parameters			
a (Å)	13.2765(7)	10.6523(14)	13.6522(5)
b (Å)	13.6027(7)	13.3466(18)	13.6522(5)
c (Å)	19.7254(11)	13.5912(18)	53.130(2)
α (deg)	82.139(1)	109.800(2)	90
β (deg)	84.547(1)	100.880(2)	90
γ (deg)	77.580(1)	111.292(2)	120
V (Å^3)	3438.3(3)	1583.2(4)	8575.8(7)
Space group	$P\bar{1}$ (# 2)	$P\bar{1}$ (# 2)	*R* 3*c* (# 161)
Z value	2	1	6
ρ_{calc} (g / cm^3)	1.220	1.245	1.248
μ (Mo Kα) (mm^{-1})	0.196	0.083	0.288
Temperature (K)	100	100	294
$2\Theta_{max}$ (°)	50.00	50.00	56.00
No. Obs. (I > 2σ(I))	8168	4053	4195
No. Parameters	764	401	237
Goodness of fit	1.014	1.016	1.164
Max. shift in cycle	0.001	0.000	0.001
Residuals*:R1; wR2	0.1086; 0.2963	0.0502; 0.1256	0.0543; 0.1571
Absorption Correction, Max/min	Multi-scan 0.7457/0.6787	Multi-scan 0.7457/0.5988	Multi-scan 0.7461/0.6811
Largest peak in Final Diff. Map (e$^-$ / Å^3)	2.065	0.768	0.860

*$R1 = \Sigma_{hkl}(||F_{obs}| - |F_{calc}||)/\Sigma_{hkl}|F_{obs}|$; $wR2 = [\Sigma_{hkl}w(|F_{obs}| - |F_{calc}|)^2/\Sigma_{hkl}wF_{obs}^2]^{1/2}$, $w = 1/\sigma^2(F_{obs})$; $GOF = [\Sigma_{hkl}w(|F_{obs}| - |F_{calc}|)^2/(n_{data} - n_{vari})]^{1/2}$.

SUPPLEMENTARY REFERENCES

1. Ogoshi, T.; Kanai, S.; Fujinami, S.; Yamagishi, T.-a.; Nakamoto, Y., para-Bridged Symmetrical Pillar[5]arenes: Their Lewis Acid Catalyzed Synthesis and Host–Guest Property. *J. Am. Chem. Soc.* **2008**, *130* (15), 5022-5023.

2. Han, C.; Zhang, Z.; Yu, G.; Huang, F., Syntheses of a Pillar[4]arene[1]quinone and a Difunctionalized Pillar[5]arene by Partial Oxidation. *Chem. Commun.* **2012**, *48* (79), 9876-9878.

3. Pan, M.; Xue, M., A Pillar[2]arene[3]hydroquinone which Can Self-Assemble to Form a Molecular Zipper in the Solid State. *RSC Advances* **2013**, *3* (43), 20287-20290.

4. Jie, K.; Zhou, Y.; Li, E.; Li, Z.; Zhao, R.; Huang, F., Reversible Iodine Capture by Nonporous Pillar[6]arene Crystals. *J. Am. Chem. Soc.* **2017**, *139* (43), 15320-15323.
