# Supplementary material for: Visualization and quantitation of electronic communication pathways in a series of redox-active pillar[6]arene-based macrocycles
Source: Commun Chem. 2020 Aug 13;3:117. doi: 10.1038/s42004-020-00363-4 (PMC9814560; doi:10.1038/s42004-020-00363-4)
Supplement: Supplementary file 2 — Description of Additional Supplementary Files [file 42004_2020_363_MOESM2_ESM.pdf]

Description of Supplementary Data

Supplementary Data 1. Crystallographic information file for P1Q.

Supplementary Data 2. Crystallographic information file for P2Q-A.

Supplementary Data 3. Crystallographic information file for P3Q-A.
