# Supplementary material for: Visualization and quantitation of electronic communication pathways in a series of redox-active pillar[6]arene-based macrocycles
Source: Commun Chem. 2020 Aug 13;3:117. doi: 10.1038/s42004-020-00363-4 (PMC9814560; doi:10.1038/s42004-020-00363-4)
Supplement: Supplementary file 6 — Peer Review Files [file 42004_2020_363_MOESM6_ESM.pdf]

Reviewers' comments:

Reviewer #1 (Remarks to the Author):

In this manuscript, Kaifer and coworkers report the synthesis, structural characterization, and electrochemical behavior of a series of partially oxidized pillar[6]arenes, obtained by the stepwise oxidation of 1,4-diethoxypillar[6]arene with cerium(IV)ammonium nitrate. Interesting distinctions can be drawn between these pillar[6]arene derivatives and their counterparts in the pillar[5]arenes, that is, the actual geometries of these macrocycles are better described as Non-Euclidean hyperbolic hexagons and elliptic pentagons, respectively. Besides, rotations of the benzoquinone units around the macrocyclic belt provided a mechanism for direct electronic communication among neighboring quinone units. Computational study to determine anisotropic induced current density (ACID) helps to visualize and quantify the through-space and through-bond communication pathways along the macrocyclic belt in these derivatives. Another very important point is that the electrochemical behavior of these pillar[6]arene derivatives were also well investigated by various characterization methods, such as convoluted cyclic voltammograms and simulated square wave voltammograms, which make the viewpoint of electronic communication in these compounds more intuitive and concrete. Overall, this work is novel, interesting, and solid. I strongly recommend its publication in Communications Chemistry after minor revisions stated below:

1. Introduction part, first sentence: pillar[n]arenes were first reported by Ogoshi and coworkers in 2008, 12 years ago. Therefore, the authors should use a more accurate expression instead of "10-15 years".
2. Introduction part, second sentence: pillararene chemistry has been developing very fast especially during the past several years. The authors only cited a few old reviews mostly published in 2012. In order to give the readers a better picture, more related recent reviews should be added to support this claim in the introduction part, e.g., Chem 2018, 4, 2029-2053; Theranostics 2019, 9, 3075-3093; Chem. Soc. Rev. 2018, 47, 5491-5528; Acc. Chem. Res. 2018, 51, 2064-2072..
3. Single crystal structure of the benzoquinones of five-membered ring of pillararene was reported in the literature. Selective oxidation of six-membered ring of pillararene and the separation of the complicated oxidized compounds are hard to achieve, not mention the difficulty of obtaining each single crystal structure. The authors tackled this difficult problem, which will be a good contribution to the field. Very impressive. However, the authors claimed "In general terms, our results indicate that the accumulation of adjacent benzoquinone units in the macrocycle is not favored." But there is one literature report on the successful synthesis and single-crystal structure of the benzoquinones of six-membered ring of pillararene derivative (leaning pillar[6]arene) with the accumulation of two pairs of adjacent benzoquinone units, where the authors used a different strategy for high yield synthesis (see LP6Q in Angew. Chem. Int. Ed. 2018, 57, 9853-9858). This reference should be cited in this claim as a typical excluded example to back this up.
4. Page 7, the sentence of "we must conclude that the…prevent full signal averaging" is unclear, which should be clarified.

Reviewer #2 (Remarks to the Author):

In this manuscript A. E. Kaifer et al. reported the syntheses, characterizations, and electrochemical behaviors of partially oxidized pillarquinones derived from 1,4-diethoxypillar[6]arene. This is a very nice follow-up work of their previous report on the corresponding pillar[5]arene systems (J. Org. Chem. 2017, 82, 8590; J. Am. Chem. Soc 2015, 137, 9788). The synthesis part, in particular separation and characterization, were taken care by the authors with impeccable quality control. Dealing with partially oxidized pillar[6]arenes is not a trivial task, as there are many more products and their constitutional isomers. After that, the intramolecular electronic communications

of this series of redox active macrocycles compounds were elucidated through both experiments and theoretical calculations. The DFT calculation and ACID study, as well as electronic absorption experiments described interesting peripheral communication, supporting the strong conjugation throughout the macrocycles. The electrochemical investigation was also quite comprehensive and gives valuable information on the intramolecular electron communication.

The technical "degree of difficulty" demonstrated is definitely high, but this paper reads more like a sequel to the JOC mentioned above. This one is more accomplished, Neither the experimental design nor the approach goes beyond the scope of their previous work. Personally, I am slightly doubtful if this manuscript fits the criteria of Communications Chemistry, especially the novelty part, but I understand this can be a very subjective measure. I would still recommend this paper for publication for the strong expertise and solid results demonstrated.

Here are some suggestions to further improve the manuscript:

Page 6 & 7, the assignment of P2Q-A concluded from the NMR spectra looks correct, even though the number of methyl signals is larger than expected. Did the authors try to record NMR data at elevated temperatures to confirm that this is indeed a result of hindered rotation? It might be interesting to take a look into the rotational energy barriers of these pillar[6]arene derivatives. The inversion barrier of 1,4-diethoxypillar[5]arene is only around 11 kcal/mol (Ogoshi et al. J. Org. Chem. 2010, 75, 3268). It seems a bit counterintuitive that a similar pillar[6]arene-derivative would have a higher barrier, since the larger cavity size should impose less steric hinderance for the rotational movement. As far as the reviewer is concerned, no inversion barrier of pillar[6]arene systems has been reported yet so far. A series of dynamic NMR experiments will offer valuable insight, but this is of course beyond the scope of the current work.

Page 7, "Figure 3. (A) 1H NMR (500 MHz) and (B) 13C NMR (100 MHz) spectra". Were 1H and 13C NMR spectra obtained in different NMR spectrometers? No information regarding NMR instrumentation can be found in the Supplementary Information.

Page 10, "both of these crystals (P1QA and P3QA) show a strong red-orange color, which is consistent with the presence of charge transfer complexes in their lattices." However, the authors did not comment on the color of P2QA crystal. As shown in Figure 7c/d, all partially oxidized pillar[6]arene derivatives show similar red-orange color in solution as a result of intramolecular interactions. Is there any significant color difference between P2QA and P1QA/P3QA crystals (or P2QA in solution and in the solid state)? Could the authors provide more evidence for the intermolecular charge transfer in the solid state?

Page 17, Figure 7a and 7b are basically identical data. Maybe simply merge these two figures?

Page 18, compound "XQ" is not defined. Does this refer to p-xyloquinone? Or is this actually benzoquinone (BQ) defined on page 6?

Page 32, Synthesis. Could the authors provide more details regarding the purification processes of each these compounds? The separation of constitutional isomers sometimes can be very difficult. This information will help other chemists to replicate their synthesis, promoting further applications of these partially oxidized pillar[6]arenes.

Page 33, "P2Q-B: 1H NMR (500 MHz,)". NMR solvent information is missing.

Reviewer #3 (Remarks to the Author):

In this very nice manuscript, the authors reported the synthesis, structural characterization and

electrochemical behavior of a novel series of macrocyclic hosts prepared by the stepwise oxidation of 1,4-diethoxypillar[6]arene with cerium(IV)ammonium nitrate, in which two, three or four 1,4-diethoxybenzene units were replaced by benzoquinone residues. A careful scrutiny of structural characteristics of the new macrocycles indicated that the actual geometries of the macrocycles were better described as Non-Euclidean hyperbolic hexagons. Rotations of the benzoquinone units around the macrocyclic belt provided a mechanism for direct electronic communication among neighboring quinone units. Using anisotropic current induced density theory (ACID), along with other density functional theory (DFT) computations, the authors identified distinct mechanisms for the electronic communication among quinone units within this family of compounds. Furthermore, convoluted cyclic voltammograms and simulated square wave voltammograms, as well as UV-vis spectra, of the new macrocycles afforded insights into the various electronic communication pathways existing in these compounds. I believe that this manuscript, with great scientific value and novelty, should be of great interest to readers of Communications Chemistry. Therefore, it is recommended to be published in Communications Chemistry after following very minor revisions:
1) In Huang’s article (Chemical Communications 48, 9876-9878, doi:10.1039/c2cc35498e (2012)), the yield for P’1Q was 30%. In Ogoshi’s article (J. Org. Chem. 77, 11146-11152, doi:10.1021/jo302283n (2012)), the yield for P1Q was only 13%. However, in this manuscript, the yield of P1Q reached 73.2%. There was no obvious difference in the synthesis process of the three articles. Please show more details to explain the reason for the high yield of P1Q.
2) In Figure7, the meaning of XQ was not clear, please explain the meaning of XQ.
3) Two important papers related to Pillar[4]arene[1]quinone should be cited: ACS Appl. Mater. Interfaces 10, 23147-23153, doi: 10.1021/acsami.8b063969 (2018); J. Am. Chem. Soc. 2018, 140, 15070-15079, doi:10.1021/jacs.8b10192 (2018).

Response to Specific Reviewers' Comments (our responses are in blue)

RE: Ms. No. COMMSCHEM-20-0152-T

Reviewer #1

1. Introduction part, first sentence: pillar[n]arenes were first reported by Ogoshi and coworkers in 2008, 12 years ago. Therefore, the authors should use a more accurate expression instead of "10-15 years".

R: We have made this change.

2. Introduction part, second sentence: pillararene chemistry has been developing very fast especially during the past several years. The authors only cited a few old reviews mostly published in 2012. In order to give the readers a better picture, more related recent reviews should be added to support this claim in the introduction part, e.g., Chem 2018, 4, 2029-2053; Theranostics 2019, 9, 3075-3093; Chem. Soc. Rev. 2018, 47, 5491-5528; Acc. Chem. Res. 2018, 51, 2064-2072..

R: We have added these four references and thank the reviewer for the suggestion.

3. Single crystal structure of the benzoquinones of five-membered ring of pillararene was reported in the literature. Selective oxidation of six-membered ring of pillararene and the separation of the complicated oxidized compounds are hard to achieve, not mention the difficulty of obtaining each single crystal structure. The authors tackled this difficult problem, which will be a good contribution to the field. Very impressive. However, the authors claimed "In general terms, our results indicate that the accumulation of adjacent benzoquinone units in the macrocycle is not favored." But there is one literature report on the successful synthesis and single-crystal structure of the benzoquinones of six-membered ring of pillararene derivative (leaning pillar[6]arene) with the accumulation of two pairs of adjacent benzoquinone units, where the authors used a different strategy for high yield synthesis (see LP6Q in Angew. Chem. Int. Ed. 2018, 57, 9853-9858). This reference should be cited in this claim as a typical excluded example to back this up.

R: We have added this reference. The compound reported there is similar to one of our new compounds, P4Q-A, although its preparation method is different. We thank the reviewer for this suggestion.

4. Page 7, the sentence of "we must conclude that the…prevent full signal averaging" is unclear, which should be clarified.

R: See response to comment #1 from reviewer #2.

Reviewer #2

1. Page 6 & 7, the assignment of P2Q-A concluded from the NMR spectra looks correct, even though the number of methyl signals is larger than expected. Did the authors try to record NMR data at elevated temperatures to confirm that this is indeed a result of hindered rotation? It might be interesting to take a look into the rotational energy barriers of these pillar[6]arene derivatives. The inversion barrier of 1,4-diethoxypillar[5]arene is only around 11 kcal/mol (Ogoshi et al. J. Org. Chem. 2010, 75, 3268). It seems a bit counterintuitive that a similar pillar[6]arene-derivative would have a higher barrier, since the larger cavity size should impose less steric hinderance for the rotational movement. As far as the reviewer is concerned, no

inversion barrier of pillar[6]arene systems has been reported yet so far. A series of dynamic NMR experiments will offer valuable insight, but this is of course beyond the scope of the current work.

R: This is an excellent point, which led us to record again the H-1 NMR spectrum for compound P2Q-A to discover that the "complexity" of the previous spectrum was the result of a shimming problem. We have now corrected this mistake and report the simpler spectrum in the supplementary information. We have also confirmed that the remaining NMR spectra reported in our manuscript are free from shimming issues. Of course, we have also removed the sentence in the manuscript in which we referred to the spectral complexity and its possible origins. We thank the reviewer for bringing this issue to the forefront, which led us to a careful reexamination of this NMR spectrum.

2. Page 7, "Figure 3. (A) 1H NMR (500 MHz) and (B) 13C NMR (100 MHz) spectra". Were 1H and 13C NMR spectra obtained in different NMR spectrometers? No information regarding NMR instrumentation can be found in the Supplementary Information.

R: We used 500 MHz and 400 MHz Bruker NMR spectrometers to record our spectra. Of course, the C-13 NMR (100 MHz) spectra were recorded in the 400 MHz instrument. We added more information on NMR instrumentation to the methods section of the manuscript.

3. Page 10, "both of these crystals (P1QA and P3QA) show a strong red-orange color, which is consistent with the presence of charge transfer complexes in their lattices." However, the authors did not comment on the color of P2QA crystal. As shown in Figure 7c/d, all partially oxidized pillar[6]arene derivatives show similar red-orange color in solution as a result of intramolecular interactions. Is there any significant color difference between P2QA and P1QA/P3QA crystals (or P2QA in solution and in the solid state)? Could the authors provide more evidence for the intermolecular charge transfer in the solid state?

R: Compound P2Q-A is also orange-red in the solid state (see Figure below). We have made some small changes to the text at the bottom of page 10 and top of page 11 to make this clear. It is not easy to differentiate the intermolecular and intramolecular charge transfer components in the solid state, but we would anticipate that the intermolecular components would be strongly attenuated at low to moderate concentrations in solution.

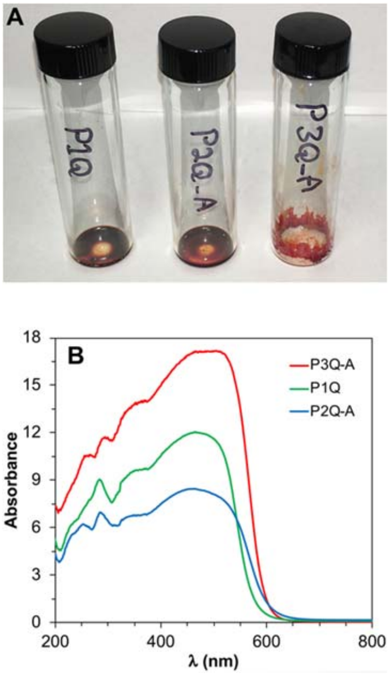

Figure 1. (A) Solid state colors and (B) diffuse reflectance UV-Vis spectra of P1Q, P2Q-A and P3Q-A compounds.

4. Page 17, Figure 7a and 7b are basically identical data. Maybe simply merge these two figures?
R: Figure 7B (Figure 5B in the revised version) is the expanded area corresponding to the quinone region, providing better resolution.
5. Page 18, compound "XQ" is not defined. Does this refer to p-xyloquinone? Or is this actually benzoquinone (BQ) defined on page 6?
R: It is *p*-xyloquinone. XQ is now defined in the text of the manuscript and the figure caption.
6. Page 32, Synthesis. Could the authors provide more details regarding the purification processes of each these compounds? The separation of constitutional isomers sometimes can be very difficult. This information will help other chemists to replicate their synthesis, promoting further applications of these partially oxidized pillar[6]arenes.
R: We report the chromatographic conditions for each of the separations required to isolate all new compounds. We have considered this issue carefully and believe that our experimental synthetic details are thoroughly described.
7. Page 33, "P2Q-B: 1H NMR (500 MHz,)". NMR solvent information is missing.
R: We added the solvent, which is deuterated chloroform.

Reviewer #3
1. In Huang's article (Chemical Communications 48, 9876-9878, doi:10.1039/c2cc35498e (2012)), the yield for P'1Q was 30%. In Ogoshi's article (J. Org. Chem. 77, 11146-11152, doi:10.1021/jo302283n (2012)), the yield for P1Q was only 13%. However, in this manuscript, the yield of P1Q reached 73.2%. There was no obvious difference in the synthesis process of the three articles. Please show more details to explain the reason for the high yield of P1Q.

R: We believe that the use of a mixed solvent (1:1 THF:CH_2Cl_2) for the reaction is important, as it provides a compromise between the water miscibility of THF for the dissolution of the oxidizing agent and higher solubility of the pillar[6]arene and the quinone-containing products in dichloromethane, resulting in a homogeneous reaction mixture and achieving high yield isolation of the products, as reported in our previous paper (*JOC* **2017**, *82*, 8590) and also by Stoikov (*New J. Chem*. **2015**, *39*, 9215).

2. In Figure 7, the meaning of XQ was not clear, please explain the meaning of XQ.

R: See response to comment #5 from reviewer #2.

3. Two important papers related to Pillar[4]arene[1]quinone should be cited: ACS Appl. Mater. Interfaces 10, 23147-23153, doi: 10.1021/acsami.8b063969 (2018); J. Am. Chem. Soc. 2018, 140, 15070-15079, doi:10.1021/jacs.8b10192 (2018).

R: We can certainly cite these two papers, but are a bit hesitant since they are concerned with pillar[5]arene compounds containing a single quinone.

Overall, we thank the reviewers for all these comments, which helped us improve the quality and clarity of the manuscript.

REVIEWERS' COMMENTS:

Reviewer #2 (Remarks to the Author):

Editor's note: this reviewer provided no comments to the author.

Reviewer #3 (Remarks to the Author):

Accept
